# A mass spectrometry-based approach for the identification of Kpnβ1 binding partners in cancer cells

**DOI:** 10.1038/s41598-022-24194-6

**Published:** 2022-11-23

**Authors:** Michael O. Okpara, Clemens Hermann, Pauline J. van der Watt, Shaun Garnett, Jonathan M. Blackburn, Virna D. Leaner

**Affiliations:** 1 Division of Medical Biochemistry and Structural Biology, University of Cape Town, Cape Town, South Africa; 2 Division of Chemical and Systems Biology, Department of Integrative Biomedical Sciences, Faculty of Health Sciences, University of Cape Town, Cape Town, South Africa; 3 Institute of Infectious Diseases and Molecular Medicine, University of Cape Town, Cape Town, South Africa; 4 SAMRC Gynaecology Cancer Research Centre, University of Cape Town, Cape Town, South Africa

**Keywords:** Cancer, Proteomics

## Abstract

Karyopherin beta 1 (Kpnβ1) is the principal nuclear importer of cargo proteins and plays a role in many cellular processes. Its expression is upregulated in cancer and essential for cancer cell viability, thus the identification of its binding partners might help in the discovery of anti-cancer therapeutic targets and cancer biomarkers. Herein, we applied immunoprecipitation coupled to mass spectrometry (IP-MS) to identify Kpnβ1 binding partners in normal and cancer cells. IP-MS identified 100 potential Kpnβ1 binding partners in non-cancer hTERT-RPE1, 179 in HeLa cervical cancer, 147 in WHCO5 oesophageal cancer and 176 in KYSE30 oesophageal cancer cells, including expected and novel interaction partners. 38 binding proteins were identified in all cell lines, with the majority involved in RNA metabolism. 18 binding proteins were unique to the cancer cells, with many involved in protein translation. Western blot analysis validated the interaction of known and novel binding partners with Kpnβ1 and revealed enriched interactions between Kpnβ1 and select proteins in cancer cells, including proteins involved in cancer development, such as Kpnα2, Ran, CRM1, CCAR1 and FUBP1. Together, this study shows that Kpnβ1 interacts with numerous proteins, and its enhanced interaction with certain proteins in cancer cells likely contributes to the cancer state.

## Introduction

Karyopherin beta 1 (Kpnβ1) is an important member of the superfamily of nuclear transport proteins responsible for shuttling cargoes into the nucleus, through the nuclear pore complex (NPC). It is a 97 kDa protein with a flexible super-helical structure composed of 19 tandem HEAT repeat units. It carries out its nuclear import function by binding to the NPC and cargo proteins at its central and C-terminals (HEAT repeats 4–19) and binds RanGTP at its N-terminus (HEAT repeats 1–8)^1–3^. In the classical nuclear transport process, Kpnβ1 commonly uses an adaptor protein, of the Karyopherin alpha (Kpnα) protein family, to facilitate protein import. The Kpnα protein recognizes cargo proteins containing a classical nuclear localization signal (cNLS)^4^. The cargo-bound Kpnα subsequently binds Kpnβ1, which transports the trimeric complex through the NPC. Once inside the nucleus Ran-GTP binds, and the cargo is released. Not all cargoes, however, contain a cNLS and many non-classical NLS motifs exist that do not require an adaptor protein like Kpnα for nuclear import. These proteins may bind Kpnβ1 directly or use another karyopherin beta family member for nuclear import^4^.

Apart from its principal function as the major nuclear importer of cargo proteins, Kpnβ1 has been implicated in other important cellular functions including the negative regulation of spindle assembly during mitosis^5–8^, regulation of the actin cytoskeleton^9^, endoplasmic reticulum-associated degradation of misfolded proteins^10^, protein chaperoning^6^, the permeability of NPCs^11^, RNA binding/processing^12^ and restructuring of the nuclear envelope and NPCs^13,14^. The various roles which Kpnβ1 plays suggest that numerous proteins might be interacting with it as binding partners.

Kpnβ1 expression has been reported to be increased in cancer. We previously found its expression to be increased in cervical cancer^15^, and other studies have recently identified increased Kpnβ1 expression in ovarian cancer^16,17^, glioma^18^, prostate cancer^19^, non-small cell lung cancer^20^ and breast cancer^21^. We previously found that a tight balance of Kpnβ1 expression is required in cancer cells, as perturbation of this balance via overexpression or inhibition results in negative cellular effects^22^, reinforcing that Kpnβ1 function is critical to cancer cell biology. Furthermore, we have identified a novel small molecule that can inhibit Kpnβ1 function and propose that targeting Kpnβ1 could have potential as an anti-cancer strategy^23^.

Recently, two studies have investigated Kpnβ1 binding partners, in an effort to subcategorize cargo proteins according to their Karyopherin β import receptor^24^, and to identify proteins that bind Kpnβ1 during mitosis^25^. To our knowledge, however, no studies have identified and compared the binding partners of Kpnβ1 in normal and cancer cells. Knowledge of the proteins that associate with Kpnβ1 in normal and cancer cells might assist in understanding the role of deregulated expression of Kpnβ1 in cancer. Furthermore, the binding partners of Kpnβ1 which are enriched in cancer cells can be investigated further as potential anti-cancer therapeutic targets or biomarkers. In this study, co-immunoprecipitation (IP) of Kpnβ1 and its binding partners was carried out using normal, cervical cancer and oesophageal cancer cell extracts, followed by high-throughput mass spectrometry (MS). IP coupled to MS is a powerful and sensitive technique for discovering and identifying binding partners of a target protein with the ability to identify hundreds of binding partners at once in a single sample^26–29^. The co-immunoprecipitants of Kpnβ1 were then compared in the normal and cancer cell extracts to identify potential anti-cancer therapeutic targets or biomarkers.

## Results

### Immunoprecipitation of Kpnβ1 in normal and cancer cell lines

**Figure 1:**
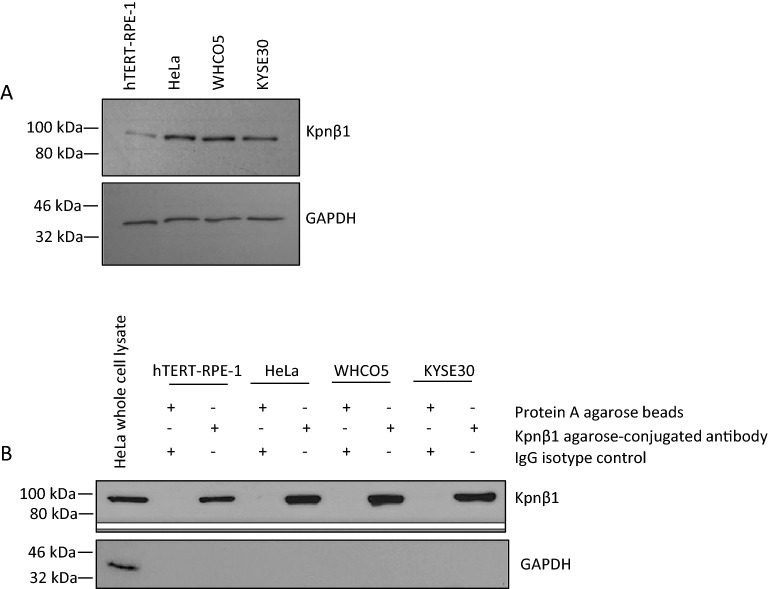
Immunoprecipitation of Kpnβ1. (**A**) Endogenous Kpnβ1 protein levels in non-cancer hTERT-RPE-1 cells, HeLa cervical cancer cells and WHCO5 and KYSE30 oesophageal cancer cells. GAPDH was used to control for protein loading. (**B**) Kpnβ1 was successfully immunoprecipitated using 50 µg of a Kpnβ1 agarose-conjugated antibody from hTERT-RPE-1, HeLa, WHCO5 and KYSE30 cell extracts. Kpnβ1 was not pulled down in lysates incubated with a non-specific IgG isotope control and protein A agarose beads. GAPDH was not pulled down with Kpnβ1, as expected.

In order to identify Kpnβ1 binding partners in normal and cancer cells, co-immunoprecipitation (co-IP) assays were performed. Incubation of 50 µg anti-Kpnβ1 agarose-conjugated antibody incubated with 500 µg HeLa whole cell lysate was found to be sufficient to pull down high levels of Kpnβ1 protein (more than that obtained using 25 µg of the agarose-conjugated antibody) (Supplementary fig. S1). Co-immunoprecipitation experiments were thus performed using optimized experimental conditions, and Kpnβ1 pull-down was determined by Western blot analysis in hTERT-RPE-1 non-cancer cells, HeLa cervical cancer and WHCO5 and KYSE30 oesophageal cancer cells. Results showed that Kpnβ1 was immunoprecipitated from both normal and cancer cell extracts, with the relative amounts of Kpnβ1 immunoprecipitated largely matching relative endogenous levels (Fig. 1A, B). Kpnβ1 was not detected in extracts in which the IgG isotype control was used instead of the Kpnβ1-specific antibody, confirming that the interaction was specific.

### Co-immunoprecipitation of Kpnβ1 and high-throughput identification of binding partners by IP-MS

Having established that the co-IP protocol was effective in pulling down Kpnβ1, immunoprecipitation coupled to mass spectrometry (IP-MS) was next performed in order to identify the binding partners of Kpnβ1 in cancer and non-cancer cells. Total protein was isolated from cell lines in biological triplicates and Kpnβ1 immunoprecipitated under optimised conditions, digested into peptides and measured on a Q Exactive mass spectrometer to identify Kpnβ1 binding partners. To determine the level of non-specific binding, an IgG isotype control was used to generate negative control samples in triplicate. All mass spectrometry data was processed using MaxQuant^30^. Proteins with q-values greater than 0.01 were excluded, and other data cleaning steps were performed. Correlation analyses were performed using log2-transformed intensity values and revealed high Pearson correlation coefficients amongst Kpnβ1 pull-down replicates of the same cell line (> 0.93) (Supplementary fig. S2A), but lower correlation coefficients within replicates for the IgG control samples, as expected for non-specifically interacting proteins (Supplementary fig. S2B). Proteins that were identified in all isotype control triplicate samples for each cell line are shown in supplementary tables S6–S7 (where 69 proteins were identified in hTERT-RPE-1 isotype controls, 37 in HeLa, 69 in WHCO5 and 90 in KYSE30).

To obtain an extended list of Kpnβ1 binding partners in each cell line, any protein identification that was observed in the isotype control samples was subtracted from the dataset. Furthermore, proteins were only retained if they were identified in all three replicate samples per cell line. Analysis of the protein groups identified in each cell line revealed that there were more Kpnβ1 binding partners in the cancer cell extracts compared to the normal cell extracts: 100 proteins were identified as potential binding partners of Kpnβ1 in normal hTERT-RPE1 cell extracts, while 179 proteins were pulled down with Kpnβ1 in HeLa cells, and 147 and 176 proteins were pulled down with Kpnβ1 in WHCO5 and KYSE30 oesophageal cancer cells, respectively (Supplementary fig. S3, numbers shown in the Venn diagrams include Kpnβ1). The full lists of proteins identified as potential binding partners of Kpnβ1 in hTERT-RPE1, HeLa, WHCO5 and KYSE30 cells extracts are shown in supplementary tables S1-4. These results indicate an approximately 1.5 to 1.8-fold larger interactome of Kpnβ1 in the cancer cell lines than in the non-cancer cell line.

Examination of the proteins identified to bind Kpnβ1 revealed many expected Kpnβ1 interaction partners, including nuclear transport proteins such as Ran and Kpnα2. Interestingly, no other Kpnα family member was pulled down with Kpnβ1, suggesting that Kpnα2 is its preferred adaptor protein, at least under the cellular conditions used in this study. Proteins known to regulate Ran function, including Ran-binding proteins (RanBP2, RanBP9) and Ran GTPase-activating protein 1 (RanGAP1) were identified as Kpnβ1 interactors in at least one cell line. Nuclear pore complex proteins were also identified, as expected, including Nup93, Nup214, Nup62, Nup98 and Nup188. However, other expected binding partners were not identified, such as Importin 7, with which Kpnβ1 heterodimerises to import basic cargoes like histone H1^6^. The omission of proteins expected to bind Kpnβ1 might be due to the stringent filtering criteria used in the study or the chosen experimental conditions, rather than these proteins not binding Kpnβ1. Importantly, IP-MS only identifies relatively stable complexes, rather than transient complexes, as no cross-linking was performed prior to pull-down.

The lists of Kpnβ1 binding partners identified in each cell line were imported into the STRING database (www.string-db.org)^31^ for further investigation. STRING-db predicts the molecular interactions amongst proteins and revealed an extended network of protein–protein interactions in each cell line, with Kpnβ1 a central node in each network. There were significantly more interactions than expected, with a protein–protein interaction (PPI) enrichment p-value of < 1.0e−16 in each cell line (Supplementary fig. S4-7).

To evaluate the functional significance of the proteins identified in each cell line, these proteins were subjected to gene ontology analysis using the PANTHER database (www.pantherdb.org)^32^. The top 10 most significantly enriched pathways/compartments in each gene ontology category were identified. Results showed that the most significantly enriched biological process, cellular component and molecular function in each cell line were gene expression, ribonucleoprotein complex, and RNA binding, respectively (Supplementary fig. S4-7). The identification of gene expression as the most significantly enriched biological process coincides with findings described by Kimura et al. (2017), who examined Kpnβ1 binding proteins in HeLa cells and compared proteins that bind Kpnβ1 to those that interact with other human import receptors^24^. Kimura et al.^24^ reported that many of the Kpnβ1-specific binding proteins they identified are related to the initial stages of gene expression, including chromatin regulation and transcriptional regulation. Many of these cargo proteins were also identified in our study, including the SWI/SNF-related regulator of chromatin, SMARCE1, the SWI/SNF complex subunit, SMARCC2, TATA-binding protein-associated factor, TAF15, and mediator of RNA polymerase II transcription subunit 15, MED15. The binding of these proteins to Kpnβ1 reinforces that Kpnβ1 function is critical to central processes governing cell biology. Kimura et al. (2017) did not use an immunoproteomics approach, but rather used an in vitro reconstituted nuclear transport system and SILAC technology followed by mass spectrometry^24^. Despite very different experimental approaches, our dataset had significant overlap (nearly 20%) with that obtained by Kimura et al. (2017), where 31 of the 179 proteins identified in our study as Kpnβ1 binding partners in HeLa cells were common to their identified list of Kpnβ1 binding proteins.

Importantly, our list of proteins also showed considerable overlap with Kpnβ1 binding partners identified by Di Francesco et al.^12^, who examined the interactome of Kpnβ1 in mitotic HeLa cells (Kpnβ1 is known to play an integral role in mitosis). This highlights the fact that the cell populations examined in our study comprised mitotic as well as interphase cells, as would be expected of asynchronously growing cells. In line with their study, we identified various tubulin proteins (TUBG1, TUBA4A, TUBB6) as Kpnβ1 interaction partners; this fits with the major role of Kpnβ1 in microtubule organisation during mitosis. Like their study we also identified BUB3, a member of the mitotic checkpoint complex, as a Kpnβ1 interactor. Kpnβ1’s interaction with BUB3 plays an important role in preventing premature anaphase^33^. Additionally, Clathrin, identified in their study as a novel Kpnβ1 binding partner that plays a role in regulating spindle functions during mitosis, was amongst the list of proteins identified in our study to be interacting with Kpnβ1, in normal and cancer cells (CLTA and CLTB)^12^.

As well as identifying Kpnβ1 binding proteins common to those identified in previous studies, a comparison analysis was also performed with various interactome databases, including BioGRID (thebiogrid.org), BioPlex (bioplex.hms.harvard.edu) and STRING-db, where Venn diagrams were drawn to identify common proteins. A comparison with the dataset in BioGRID revealed 42 common proteins (10% of the proteins listed as Kpnβ1-interacting proteins in BioGRID were in our dataset) (Supplementary fig. S8A). BioGRID compiles data from numerous publications using a wide array of methods to identify interaction partners and various cell models, hence this percentage overlap was expected. A comparison with the BioPlex interactome revealed an overlap of 9 proteins, yet only 40 are listed, hence 29% of the proteins were identified as common to our dataset (Supplementary fig. S8B). Finally, a comparison with STRING-db identified an overlap of 70 proteins (65%) when experimental and datasources were selected as interaction sources in STRING-db (Supplementary fig. S8C), and 89% overlap when experimental only was selected (Supplementary fig. S8D). This high percentage of overlap provides confidence in our data generated.

Finally, while the proteins identified in our study as Kpnβ1-binding proteins were determined using a presence/absence approach, a more quantitative analysis was also undertaken, where proteins identified in the IP-MS experiment were processed using SAINTexpress to identify differentially abundant proteins. Lists of proteins identified using SAINTexpress can be found in Supplementary tables S12–S15. Those with a SAINTscore of 1 were chosen and compared to the protein lists identified in supplementary tables S1–S4, and an overlap of between 49 and 57% was identified across cell lines (Supplementary fig. S9).

### Identification of proteins common to the cancer and non-cancer cell lines

**Figure 2:**
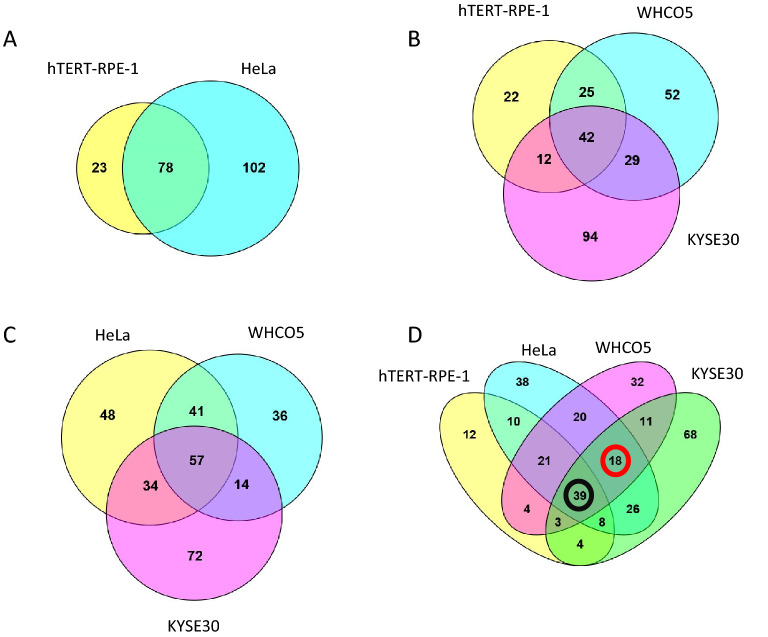
Venn diagrams of binding partners of Kpnβ1 identified using IP-MS. Venn diagrams were drawn representing overlaps in binding partners of Kpnβ1 in all replicates of (**A**) hTERT-RPE1 and HeLa cell lines, (**B**) hTERT-RPE1, WHCO5 and KYSE30 cell lines, (**C**) HeLa, WHCO5 and KYSE30 cell lines and (**D**) hTERT-RPE1, HeLa, WHCO5 and KYSE30 cell lines. The number of Kpnβ1 binding partners that are common to the normal and cancer cell lines is encircled in a black ring and the number of Kpnβ1 binding partners which are unique to the cancer cell lines is encircled in a red ring.

Having established significant overlap with other datasets of Kpnβ1 interaction partners, the lists of Kpnβ1 binding partners identified in our study were further explored. Venn diagrams were drawn, in order to identify overlapping binding partners in the normal and cancer cell lines, allowing for the identification of binding partners that are either common to all cell lines or unique to a specific cell line. As shown in Fig. 2, 77 proteins were identified as common binding partners of Kpnβ1 in normal hTERT-RPE1 and HeLa cervical cancer cells (Fig. 2A, numbers shown in the Venn diagrams include Kpnβ1), while 41 proteins were common binding partners of Kpnβ1 in hTERT-RPE1, WHCO5 and KYSE30 oesophageal cancer cell lines (Fig. 2B), and 56 proteins were identified as common binding partners of Kpnβ1 in HeLa, WHCO5 and KYSE30 cancer cells extracts (Fig. 2C). Interestingly, KYSE30 appeared to be the most distinct of all the cell lines, having the highest number of unique proteins. An overlap of the pull-down data for all four cell lines revealed that 38 proteins were common binding partners of Kpnβ1 in the four cell lines (Fig. 2D, Table 1). This group of 38 binding partners of Kpnβ1 was therefore considered as having the highest probability of being true Kpnβ1 binding partners.

**Table 1:** List of common protein hits identified by IP-MS in the Kpnβ1 co-IP experiments from human normal, cervical cancer and oesophageal cancer cells extracts.

Group/pathway/function	Protein IDs	Protein name	Gene name	Molecular weight (kDa)
Nuclear import	Q14974	Importin subunit beta-1	KPNB1	97.169
Spliceosomal component	J3QLE5	Small nuclear ribonucleoprotein-associated protein N	SNRPN	17.546
	P08621	U1 small nuclear ribonucleoprotein 70 kDa	SNRNP70	51.556
	P09012	U1 small nuclear ribonucleoprotein A	SNRPA	31.279
	P62316	Small nuclear ribonucleoprotein Sm D2	SNRPD2	13.527
	P62318	Small nuclear ribonucleoprotein Sm D3	SNRPD3	13.916
	Q13435	Splicing factor 3B subunit 2	SF3B2	100.23
	Q15637	Splicing factor 1	SF1	68.329
Heterogeneous nuclear ribonucleoproteins	A0A087WUK2	Heterogeneous nuclear ribonucleoprotein D-like	HNRNPDL	40.04
	A0A0A0MRA5	Heterogeneous nuclear ribonucleoprotein U-like protein 1	HNRNPUL1	85.939
	D6R9P3	Heterogeneous nuclear ribonucleoprotein A/B	HNRNPAB	30.302
	O43390	Heterogeneous nuclear ribonucleoprotein R	HNRNPR	70.942
	Q13151	Heterogeneous nuclear ribonucleoprotein A0	HNRNPA0	30.84
RNA binding/processing	B0QYK0	RNA-binding protein EWS	EWSR1	64.929
	P35637	RNA-binding protein FUS	FUS	53.425
	Q15717	ELAV-like protein 1	ELAVL1	36.091
	Q9Y224	UPF0568 protein C14orf166	C14orf166	28.068
RNA Helicases	A0A0D9SFB3	ATP-dependent RNA helicase DDX3X	DDX3X	70.839
	A0A1X7SBZ2	Probable ATP-dependent RNA helicase DDX17	DDX17	80.253
	P26196	Probable ATP-dependent RNA helicase DDX6	DDX6	54.416
	J3KTA4	Probable ATP-dependent RNA helicase DDX5	DDX5	69.086
Cleavage and polyadenylation	F8WJN3	Cleavage and polyadenylation specificity factor subunit 6	CPSF6	52.269
	Q05048	Cleavage stimulation factor subunit 1	CSTF1	48.357
	O43809	Cleavage and polyadenylation specificity factor subunit 5	NUDT21	26.227
Ribosomal proteins	M0R3D6	60S ribosomal protein L18a	RPL18A	16.714
	P62277	40S ribosomal protein S13	RPS13	17.222
	P62899	60S ribosomal protein L31	RPL31	14.463
NPC component	Q8N1F7	Nuclear pore complex protein Nup93	NUP93	93.487
	F5H365	Protein transport protein Sec23A	SEC23A	82.968
	P55735	Protein SEC13 homolog	SEC13	35.54
ssDNA binding/stabilization	Q96I24	Far upstream element-binding protein 3	FUBP3	61.64
	P27694	Replication protein A 70 kDa DNA-binding subunit	RPA1	68.137
SWItch/Sucrose Non-Fermentable (SWI/SNF) component	Q8TAQ2	SWI/SNF complex subunit SMARCC2	SMARCC2	132.88
	O96019	Actin-like protein 6A	ACTL6A	47.46
Vesicle transport	Q92734	Protein TFG	TFG	43.447
Regulation of gene expression	A5YKK6	CCR4-NOT transcription complex subunit 1	CNOT1	266.94
Microtubule organization	P23258	Tubulin gamma-1 chain	TUBG1	51.169
Methylation of arginyl residues	Q86X55	Histone-arginine methyltransferase CARM1	CARM1	65.853
Regulation of circadian clock	Q8WXF1	Paraspeckle component 1	PSPC1	58.743

**Figure 3:**
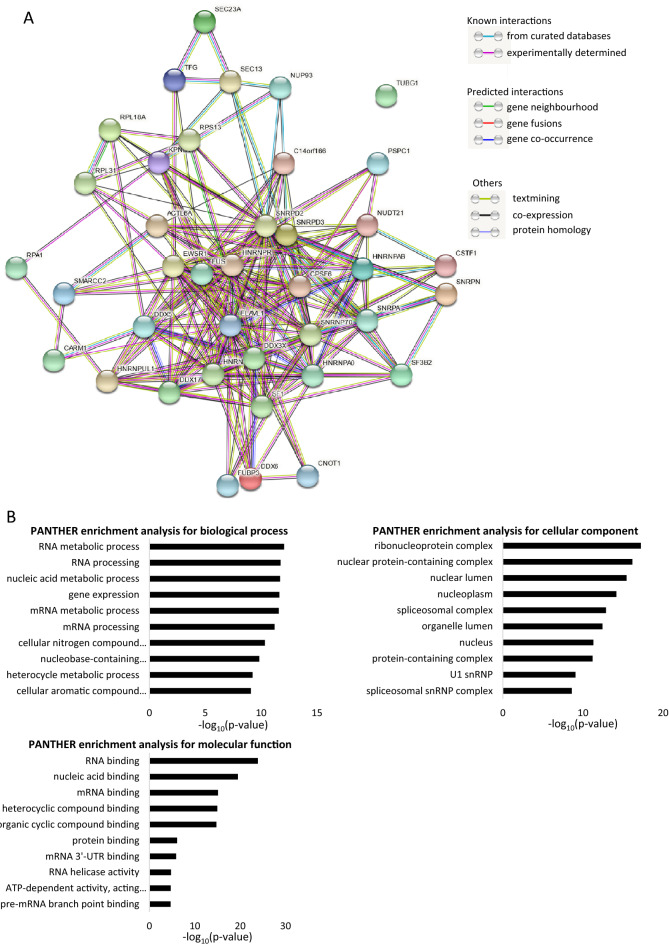
Protein–protein interaction network and gene ontology analyses of Kpnβ1 binding proteins common to cancer and non-cancer cells. (**A**) STRING protein–protein interaction (PPI) network analysis of 38 common Kpnβ1-interacting proteins reveals nearly all proteins are highly interconnected. A medium confidence level (0.4) was used. The circles represent proteins, while the straight lines represent the interactions between different proteins. (**B**) PANTHER gene ontology analysis of 38 Kpnβ1-interacting proteins common to cancer and non-cancer cells. The 10 most significantly enriched biological processes, cellular components and molecular functions are shown.

The list of 38 common Kpnβ1 binding partners was imported into the STRING database for further investigation. STRING analysis revealed that nearly all common Kpnβ1 binding partners were highly connected (Fig. 3A). To evaluate the functional significance of the proteins identified as common to the normal and cancer cells, these proteins were subjected to gene ontology analysis using the PANTHER database (see Supplementary table 10 for full PANTHER analysis). The top 10 most significantly enriched pathways/compartments in each gene ontology category were identified, and it was found that the list of common Kpnβ1 binding proteins was most significantly enriched in proteins involved in RNA metabolic processes (Fig. 3B), with 24 proteins allocated to this biological process. Gene expression was also significantly enriched, as expected, with 25 of the 38 proteins involved in gene expression. In terms of cellular component, the most significant enrichment was for ribonucleoprotein complex proteins, and for molecular function, RNA binding proteins were most significantly enriched (Fig. 3B), as observed for the individual cell lines (Supplementary fig. S4-7). Di Francesco et al.^12^ and Kimura et al.^24^ similarly described the identification of many RNA-binding proteins as novel Kpnβ1 interactors in their studies. This highlights the important role of Kpnβ1 in RNA metabolism; a role for Kpnβ1 which is often overlooked.

### Identification of proteins unique to the cancer cell lines

As well as identifying Kpnβ1 binding proteins common to all 4 cell lines, it was also of interest to identify proteins unique to the cancer cell lines, as these could play important roles in cancer biology, and might be useful future biomarkers/therapeutic targets of the disease. Overlapping the lists of Kpnβ1 binding partners for the normal and cancer cell lines revealed that 18 proteins were found in the cancer but not normal cell lines (Fig. 2D, Table 2). These proteins cannot be ruled out as Kpnβ1 binding partners in the normal cells, as it is possible that they do bind, albeit at a lower affinity or for a shorter duration, or their levels of expression might be lower in the normal cells, hence their lack of detection by the mass spectrometer.

**Table 2:** List of 18 common protein hits identified by IP-MS in the Kpnβ1 co-IP experiments unique to human cervical and oesophageal cancer cells extracts.

Group/pathway/function	Protein IDs	Protein name	Gene name	Molecular weight (kDa)
Nuclear transport	P62826	GTP-binding nuclear protein Ran	RAN	26.224
Ribosomal proteins	P18124	60S ribosomal protein L7	RPL7	29.225
	P62753	40S ribosomal protein S6	RPS6	28.68
	P27635	60S ribosomal protein L10	RPL10	18.565
	G3V203	60S ribosomal protein L18	RPL18	18.756
	P62701	40S ribosomal protein S4, X isoform	RPS4X	29.597
	M0QZC5	40S ribosomal protein S11	RPS11	13.997
	P40429	60S ribosomal protein L13A	RPL13A	23.577
Heterogeneous nuclear ribonucleoproteins	Q1KMD3	Heterogeneous nuclear ribonucleoprotein U-like protein 2	HNRNPUL2	84.69
	P31942	Heterogeneous nuclear ribonucleoprotein H3	HNRNPH3	36.926
RNA binding/processing	Q6UN15	Pre-mRNA 3-end-processing factor FIP1	FIP1L1	66.526
Cleavage and polyadenylation	Q8N684	Cleavage and polyadenylation specificity factor subunit 7	CPSF7	41.265
Spliceosomal component	J3KTL2	Serine/arginine-rich splicing factor 1	SRSF1	28.329
	A0A087X2D0	Serine/arginine-rich splicing factor 3	SRSF3	10.32
NPC component	P35658	Nuclear pore complex protein Nup214	NUP214	213.62
ssDNA binding/stabilization	Q96AE4	Far upstream element-binding protein 1	FUBP1	67.56
Motor protein	F8W1R7	Myosin light polypeptide 6	MYL6	14.436
Regulation of cell growth	Q8IX12	Cell division cycle and apoptosis regulator protein 1	CCAR1	132.82

**Figure 4:**
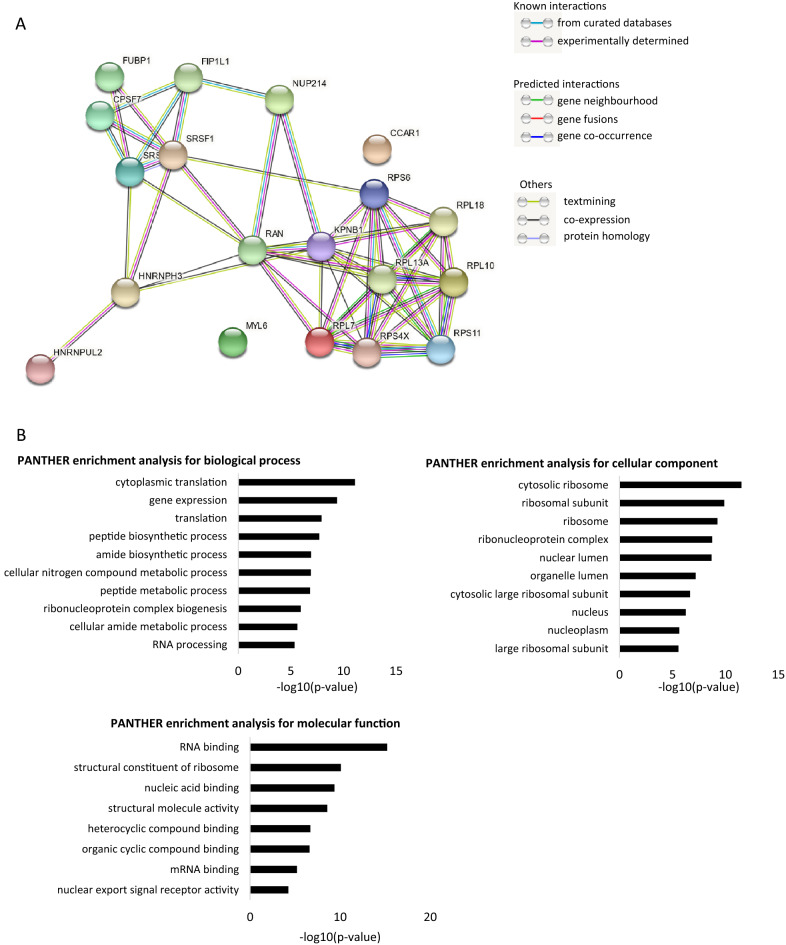
Protein–protein interaction network and gene ontology analyses of Kpnβ1 binding proteins unique to cancer cells. (**A**) STRING protein–protein interaction (PPI) network analysis of 18 unique Kpnβ1-interacting proteins reveals a high degree of protein–protein interaction. A medium confidence level (0.4) was used. The circles represent proteins, while the straight lines represent the interactions between different proteins. (**B**) PANTHER gene ontology analysis of 18 Kpnβ1-interacting proteins common to cancer and non-cancer cells. The 10 most significantly enriched biological processes, cellular components and molecular functions are shown.

STRING analysis identified interactions between nearly all 18 proteins identified in the cancer cell lines, with Kpnβ1 identified as a central node (Fig. 4A). For analysis of the functional significance of these proteins, a PANTHER gene ontology analysis was again performed (see Supplementary table 11 for full PANTHER analysis). The top 10 most significantly enriched pathways/compartments in each gene ontology category were identified (only 8 categories for molecular function were identified), and this analysis revealed that the list of Kpnβ1 binding proteins, unique to cancer cells, was most significantly enriched in proteins involved in translation (Fig. 4B), with 7 of the 18 proteins known to play a role in translation. In terms of cellular component, the most significant enrichment was for the ribosome, and for molecular function, RNA binding proteins were again most significantly enriched. The identification of a significant number of ribosomal proteins suggests that the interaction of specific ribosomal proteins with Kpnβ1 is enhanced in cancer cells, likely contributing to increased rates of protein translation.

In further interrogation of the list of proteins that bind Kpnβ1 uniquely in cancer cells, a few proteins were identified that are known to play roles in cancer-related processes, for example FUBP1 (Far upstream element-binding protein 1), a DNA-binding protein that regulates transcription of the proto-oncogene c-Myc^34^, and CCAR1 (Cell division cycle and apoptosis regulator protein 1), a transcriptional coactivator for nuclear receptors that plays a role in regulating cell growth and apoptosis^35,36^. The interaction between these proteins and Kpnβ1 has not been previously explored.

### Validation of known and novel Kpnβ1 interactions

In order to confirm the IP-MS findings and validate the interaction between Kpnβ1 and select proteins, co-immunoprecipitating complexes were subjected to Western blot analysis. We chose to validate the interaction of Kpnβ1 with known binding proteins as well as novel Kpnβ1 interactors. GAPDH was included as a negative control since it is not expected to interact with Kpnβ1.

**Figure 5:**
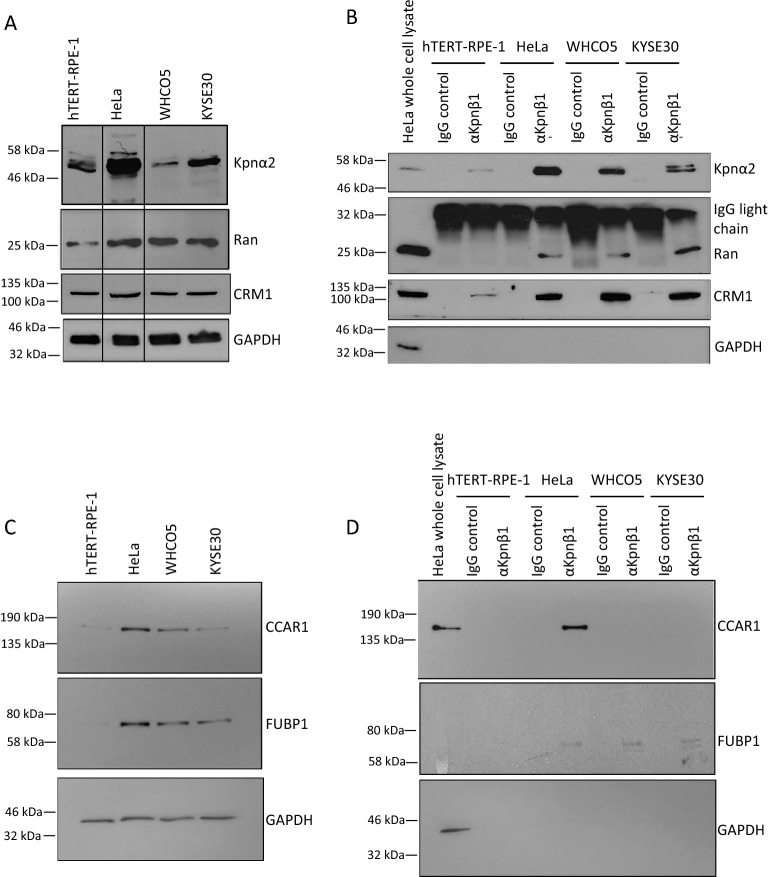
Validation of Kpnβ1 binding partners identified by IP-MS. (**A**) Expression of known Kpnβ1 interactors, Kpnα2 and Ran, and CRM1, was analysed in hTERT-RPE-1, HeLa, WHCO5 and KYSE30 whole cell lysates (30 µg) by Western blot analysis. GAPDH was used to control for protein loading. (**B**) Co-immunoprecipitation assays were performed and Kpnα2, Ran and CRM1 proteins detected by Western blot analysis. GAPDH was used as a negative control, as it is not expected to bind Kpnβ1. CRM1, Kpnα2 and Ran proteins were not pulled down in lysates incubated with a non-specific IgG isotope control and protein A agarose beads. 10 µg HeLa protein lysate was included as a positive control. (**C**) Western blot analysis showing expression of novel Kpnβ1 interactors, CCAR1 and FUBP1 in hTERT-RPE-1, HeLa, WHCO5 and KYSE30 whole cell lysates. GAPDH was used to control for protein loading. (**D**) Co-immunoprecipitation assays showing successful pull-down of CCAR1 and FUBP1 with Kpnβ1. GAPDH was used as a negative control. CCAR1 and FUBP1 were not pulled down in lysates incubated with a non-specific IgG isotope control and protein A agarose beads. Experiments were performed two times.

Known Kpnβ1 interacting proteins, Kpnα2 and Ran were first investigated. Endogenous levels were examined in whole cell lysates and higher levels of expression of Ran and Kpnα2 were noted in most of the cancer cells (Fig. 5A). Kpnα2 and Ran were next investigated in Kpnβ1 pull-down extracts, and results showed that both proteins were pulled down with Kpnβ1 in each of the cell lines, although there were higher levels of Kpnα2 and Ran present in the Kpnβ1 pull-down extracts of cancer cells compared to normal (Fig. 5B). Proteins were not detected in extracts in which the IgG isotype control was used instead of the Kpnβ1-specific antibody, confirming the specificity of the interaction. As CRM1 (Exportin 1 or Xpo1) appeared as a Kpnβ1 binding partner in the mass spectrometry results, its presence was also investigated in the Kpnβ1 pull-down extracts, as it is known to be the major exporter protein in the cell, but to our knowledge has not previously been shown to interact with Kpnβ1. Endogenous CRM1 was expressed at approximately equivalent levels in the cancer and non-cancer cells, whereas there were much higher levels of CRM1 present in the Kpnβ1 pull-down extracts of cancer cells compared to non-cancer cells (Fig. 5B). These results show that Kpnα2, Ran and CRM1 nuclear transport proteins interact with Kpnβ1, to a greater extent in cancer cells, and we propose that while these proteins are required to bind Kpnβ1 in normal cells, their interaction is substantially enriched in the cancer cells, likely contributing to faster rates of nuclear transport required by cancer cells.

To confirm the newly identified Kpnβ1 binding partners, the interactions between Kpnβ1 and FUBP1 and Kpnβ1 and CCAR1 were further investigated, as these protein interactions have not previously been validated by Western blot analysis, yet both FUBP1 and CCAR1 proteins have been implicated in cancer development. Western blot analysis was first performed to analyse levels of endogenous CCAR1 and FUBP1 in normal and cancer cells lines, and results revealed increased expression of both CCAR1 and FUBP1 in cancer cells compared to normal (Fig. 5C). CCAR1 and FUBP1 proteins were next analysed in Kpnβ1 pull-down extracts in two independent Western blot experiments, and both CCAR1 and FUBP1 were found to be enriched with Kpnβ1 in the pull-downs (Fig. 5D, Supplementary fig. S17). A strong putative CCAR1-Kpnβ1 interaction was detected in HeLa cells, but not in hTERT-RPE-1 or WHCO5 cells, and only faint levels were detected in KYSE30 cells (see Supplementary fig. S10 for detection using a more sensitive chemiluminescent substrate). FUBP1 was found to interact with Kpnβ1 in all three cancer cell lines, and although it was detected in non-cancer hTERT-RPE-1 cells, it was pulled down to a greater extent in HeLa and KYSE30 cells compared to the non-cancer cells (Fig. 5D, Supplementary fig. S10, and S17). The increased interaction of CCAR1 and FUBP1 with Kpnβ1 in cancer cells confirms the mass spectrometry data and suggests that cancer cells may display an increased reliance on CCAR1 and FUBP1 nuclear function.

Altogether, this study shows that Kpnβ1 binds numerous proteins: some unique and others common across cell types. The high number of identified binding partners, with diverse functions, also reveals that Kpnβ1 is involved in numerous cellular processes, in both interphase and mitosis, highlighting the essential role of this nuclear importer protein in normal and cancer cell biology.

## Discussion

Kpnβ1 plays multiple roles in cellular biology and as a result is expected to interact, directly or indirectly, with many other proteins. Herein, we used immunoproteomics to identify potential binding partners of Kpnβ1 in normal and cancer cell lines. Results highlighted the range of cellular processes in which Kpnβ1 is involved. Furthermore, enhanced interactions of Kpnβ1 with certain proteins were identified in cancer cells compared to normal. To the best of our knowledge, this study is the first to investigate and compare the binding partners of Kpnβ1 in normal and cancer cells and could be a useful reference for further studies.

We identified 38 proteins as common co-immunoprecipitants of Kpnβ1 in the normal and cancer cell extracts and 18 proteins as specific co-immunoprecipitants of Kpnβ1 in the cancer but not normal cells. While it is possible that these proteins only bind Kpnβ1 when cells are in the cancer state, we propose that the presence of these proteins in the cancer only group could also be due to their (1) increased expression, (2) higher affinity binding to Kpnβ1 due, for example, to a post-translational modification on one or other binding partner, or (3) increased duration of interaction with Kpnβ1 in the cancer cell lines. Thus, while their interaction with Kpnβ1 is likely enriched in cancer cells, it remains possible they do bind Kpnβ1 in non-cancer cells as well. The lack of detection of these low-abundance proteins in the non-cancer co-immunoprecipitates is also a known shortcoming of IP-MS technology, as, while it is important to co-immunoprecipitate low abundance proteins, this must be weighed up against minimising non-specific binding of proteins to the beads. Applying very stringent lysis and wash steps may help to minimise non-specific binding but at the same time can lead to loss of low abundance proteins which are true binding partners^27,37^.

Our IP-MS and IP-WB data both showed that Kpnα2, Ran and CRM1 bind Kpnβ1 to a greater extent in the cancer cell extracts with little/none detected in the normal cell extracts. There is evidence to show that Ran is overexpressed in prostate cancer, breast cancer, colon cancer and cervical cancer^38^. Kpnα2 has also been reportedly overexpressed in cervical cancer^39^, colon cancer^40^, renal cell carcinoma^41^, bladder cancer^42^ and breast cancer^43^, amongst others, as has CRM1 in cervical cancer^39^, ovarian cancer^44^, oesophageal cancer^45^ and pancreatic cancer^46^. The increased expression of these nuclear transporter proteins, together with their increased interaction with Kpnβ1 in cancer cells, supports studies that indicate that the rate of nuclear-cytoplasmic transport of cargo proteins is higher in cancer cells than in normal cells, and further highlights the potential of targeting this functional pathway as an anti-cancer therapeutic strategy. Interestingly, the interaction of CRM1 and Kpnβ1 is to our knowledge previously unreported and worth further investigation.

In our study ribosomal proteins were a major subclass of proteins found to interact with Kpnβ1, and these proteins were particularly evident in the list of proteins whose interaction with Kpnβ1 was enriched in cancer cells compared to normal. Ribosomal proteins play important roles in ribosome assembly and in ensuring the stability of ribosomal ribonucleic acid (rRNA) structure in the ribosome, thus promoting efficient protein synthesis^47^. While they are synthesized in the cytoplasm, they must be transported into the nucleus to associate with rRNAs being transcribed and form the two ribosomal subunits (large and small), which then translocate back to the cytoplasm, forming a functional ribosome^48^. There are reports which suggest that Kpnβ1 and other nuclear transport proteins are responsible for the nuclear import of ribosomal proteins^49,50^. Identifying an enhanced interaction of ribosomal proteins with Kpnβ1 in cancer cells suggests that the rate of ribosome assembly is enhanced in cancer cells compared to normal cells. Indeed, increased overall ribosome biogenesis has been reported to be a common feature of active proliferation and cancer progression, resulting in elevated protein translation^51^. It is also worth noting that the identification of a specific group of ribosomal proteins as co-immunoprecipitants of Kpnβ1 in cancer cells only suggests that these particular ribosomal proteins could potentially serve as novel anti-cancer targets. Indeed, targeting of specific ribosomal proteins, for example ribosomal protein L15, has been found to inhibit cancer growth in vitro and *in vivo*^52^. Interestingly, in our study, ribosomal protein L15 was identified as a Kpnβ1-binding partner in 2 of the 3 cancer cell lines, but not the non-cancer hTERT-RPE-1 cells.

Finally, we substantiated the associated between novel Kpnβ1 interacting proteins CCAR1 and FUBP1 and Kpnβ1 by Western blot analysis. While CCAR1-Kpnβ1 binding was detected predominantly in HeLa cells (and faintly in KYSE30 cells), it was not detected in hTERT-RPE-1 non-cancer cells, suggesting that this might be a protein that requires increased nuclear shuttling in cancer cells, in order to perform its function. CCAR1, also known as cell cycle- and apoptosis-regulatory protein-1 (CARP-1), is a perinuclear phosphoprotein which has biphasic roles in the regulation of apoptosis and cell growth, by serving as a co-activator of various nuclear receptors^35^. As a result, it displays both tumour promoting and tumor suppressing properties. Our study is the first to validate an interaction between CCAR1 and Kpnβ1, and to show that this interaction is enhanced in cancer, particularly in HeLa cervical cancer cells. We also show increased levels of endogenous CCAR1 in cervical and oesophageal cancer cell lines compared to normal. Previous reports have identified increased expression of CCAR1 in liver and renal cancer, where high expression of CCAR1 correlates with poor overall survival^53^. However, no reports have yet described the expression or role of CCAR1 in cervical or oesophageal cancer.

FUBP1 was found to associate with Kpnβ1 in all three cancer cell lines, and to a lesser extent in the non-cancer cells. FUBP1 is a DNA- and RNA-binding protein that regulates transcription, translation and RNA splicing. It plays an important role in transactivating c-myc proto-oncogene transcription^54^. Its expression has been reported to be upregulated in various cancer types, including oesophageal cancer, where it has been found to promote oesophageal cancer progression^55^. Its interaction with Kpnβ1 likely facilitates its role in the nucleus as a master gene regulator. FUBP1 expression has not been previously investigated in cervical cancer, but its high expression and enhanced interaction with Kpnβ1 in the cancer cells shown in our study warrants further investigation.

Taken together, this study identified more than one hundred known and novel candidate binding partners of Kpnβ1 in normal and cancer cell lines. Comparing the identified binding partners of Kpnβ1 in normal and cancer cell lines revealed 18 proteins as binding partners of Kpnβ1 which were enriched in the cancer cell lines compared to normal. These proteins should be investigated further in a wider range of non-cancer and cancer cell lines, as they may have future potential as anti-cancer therapeutic targets or biomarkers.

## Materials and methods

### Cell lines and cell culture

The human telomerase-immortalized retinal pigmented epithelial 1 (hTERT RPE-1) cell line and human cervical carcinoma cell line, HeLa, were purchased from American Type Culture Collection (ATCC). The human oesophageal squamous cell carcinoma (WHCO5) cell line was originally established from a South African patient with OSCC and was acquired from Professor Rob Veale at the University of Witwatersrand^56^ while human oesophageal squamous cell carcinoma cell line, KYSE30, was acquired from DSMZ. Cells were cultured in Dulbecco's Modified Eagle's Medium (DMEM) (Gibco, Life Technologies) supplemented with penicillin, streptomycin and 10% fetal calf serum (FCS) (HyClone Laboratories) except for hTERT-RPE1 cells, which were grown in DMEM/F-12 (1:1) nutrient mixture (Gibco, Life Technologies) supplemented with penicillin, streptomycin, 10% FCS and 0.01 mg/ml hygromycin B (Sigma Aldrich). Cells were maintained in a humidified incubator at 37 °C and in 5% carbon dioxide_._ Cancer cell lines were authenticated by DNA profiling using the Cell ID system (Promega, USA).

### Immunoprecipitation for Kpnβ1

Cells were grown to approximately 80% confluency and lysed using a non-denaturing lysis buffer (1% Triton X-100, 120 mM NaCl, 1 mM CaCl_2_, 25 mM Tris, pH 7.4) to retain the interactions between Kpnβ1 and its binding partners. Before co-IP and IP-MS experiments could commence, optimising the ideal antibodies concentration for immunoprecipitating Kpnβ1 was necessary. 50 μg of Anti-Karyopherin β1 (H-7) AC agarose conjugated antibody (Santa Cruz Biotechnology, sc-137016 AC) was determined to be most effective at pulling down Kpnβ1 from 500 μg of intracellular HeLa protein. A rabbit (DA1E) mAb IgG isotype control (Cell Signaling technology, #3423) that is not directed against any known antigen was included, together with protein A agarose beads (Abcam, high affinity beads ab193255), to account for proteins non-specifically binding the antibody or beads.

For the co-immunoprecipitation of Kpnβ1 and its binding partners, 500 μg of intracellular protein (from 3 biological replicates) was precleared using 50 μl of protein A agarose-conjugated beads (Abcam, high affinity beads) at 4 °C for 45 min with gentle rocking. The samples were centrifuged at 18,000×*g* for 10 min at 4 °C, then the supernatants were incubated with 50 μg of Anti-Karyopherin β1 (H-7) AC agarose conjugated antibody at 4 °C overnight with gentle rocking. 50 μl of protein A agarose (High Affinity) conjugated beads and 15 μl of IgG isotype control were added to the control sample and incubated at 4 °C overnight. The samples were centrifuged at 18,000×*g* for 3 min at 4 °C, and the pellets were washed five times with ice-cold 1 × PBS containing 1X protease inhibitor (Pierce) and centrifuging at 10,000×*g* for 3 min at 4 °C. The washed bead pellets were then subjected to further treatment for either IP-MS or IP-WB analysis.

### Proteomics

#### In-solution digestion and desalting of tryptic peptides

Immunoprecipitated proteins were eluted by incubation in 30 μl of denaturation buffer (6 M urea, 2 M thiourea, 10 mM Tris–HCl, pH 8.0) for 5 min at room temperature, and the entire eluate processed for mass spectrometry (without quantification). Proteins were reduced by incubation with dithiothreitol at a final concentration of 1 mM at room temperature (RT) for 1 h and free cysteine residues alkylated by incubation with iodoacetamide (Amresco Biochemicals and Life Science products) at a final concentration of 5.5 mM for 1 h at room temperature in the dark. The samples were diluted with 4 volumes of 20 mM ammonium bicarbonate (Sigma Aldrich) and 20 mM calcium chloride (Sigma Aldrich). Sequence-grade trypsin (New England Biolabs) was added to the samples with a protein to trypsin ratio of 50:1 and the samples were incubated for digestion at RT overnight. The digestion was stopped by addition of formic acid at a final concentration of 0.1%. Digested peptides were desalted using homemade STAGE tips with Empore™ Octadecyl solid-phase extraction disks (Supelco). STAGE tips were activated by adding 300 μl of solvent B (80% acetonitrile and 0.1% formic acid) and equilibrated by adding twice 100 μl of solvent A (2% acetonitrile and 0.1% formic acid). The samples were added to the STAGE tips and washed three times with solvent A. The bound peptides were then eluted three times by addition of 50 μl of solvent C (60% acetonitrile and 0.1% formic acid). The eluted peptides were dried in vacuo and resuspended in 50 μl of solvent A prior to measurement on a Q Exactive Mass Spectrometer (Thermo Fisher).

#### LC–MS/MS measurement

Tryptic peptides were separated by liquid chromatography on a homemade precolumn (100 μm ID × 20 mm) packed with C18 Luna beads (5 μm diameter, 100 Å pore size; Phenomenex 04A-5452) connected to an analytical column (75 μm × 200 mm) packed with Aeris C18 beads (3.6 µm diameter; Phenomenex 00B-4507-AN) connected to an Ultimate 3500 RS nano UPLC system (Dionex). Desalted peptides were loaded onto the column with a starting mobile phase of 2% ACN with ﻿0.1% formic acid and separated at a constant flow rate of 300 nL/min using the following gradient: increase to 5% ACN over 5 min, increase to 50% ACN over 15 min, to 80% ACN over 5 min, followed by a column wash of 80% for 20 min. Mass spectra were collected on a Q Exactive mass spectrometer (﻿Thermo Fisher Scientific) operated in a data-dependent manner with automatically switching between MS and MS/MS scans using a top-10 method. Peptides were ionised by electrospray ionisation and MS spectra were acquired at a resolution of 70,000 with a target value of 3 × 10^6^ ions or a maximum integration time of 250 ms. The scan range was restricted between 300 and 1750 m/z. Peptide fragmentation was performed by higher-energy collision dissociation (HCD) with the energy set at 25 NCE. Intensity threshold for ions selection was fixed at 1.7 × 10^4^ with charge exclusion of z = 1 and z > 5. The MS/MS spectra were acquired at a resolution of 17,500, with a target value of 2 × 10^5^ ions or a maximum integration time of 120 ms and the isolation window was set at 4.0 m/z.

#### Immunoprecipitation Western blot

Immunoprecipitated protein (the entire immunoprecipitate from an independent IP experiment to that performed for mass spectrometry) was resuspended in 35 μl of 2 × loading buffer without bromophenol blue. Samples were boiled at 95 °C for 5 min and centrifuged at 18,000×*g* for 3 min at RT and bromophenol blue then added to the supernatants. 35 μl sample was electrophoresed for Western blot analysis, performed using rabbit anti-Importin beta (1:5000, Abcam ab45938), rabbit anti-CRM1 (H-300) (1:1000, Santa Cruz Biotechnology, sc-5595), rabbit anti-Kpnα2 (1:2500, Abcam ab97580), mouse anti-Ran (1:500, Sigma-Aldrich R4777), rabbit anti-CCAR1 (1:1000, Novus Biologicals NB500-186), rabbit anti-FUBP1 (1:500, Novus Biologicals NBP2-16543) and mouse anti-GAPDH (0411) (1:10,000, Santa Cruz Biotechnology, sc-47724). For all IP-WB analysis, Abcam Veriblot for IP Detection Reagent (HRP) (ab131366) was used as a secondary antibody with a dilution of 1:2500 in 5% milk in TBST. For analysis of whole cell lysates, 30 µg protein was loaded onto SDS-PAGE gels and blots probed using the same antibodies mentioned above. Lumiglo (KPL) was used as the chemiluminescent substrate for Western blot detection. For all Western blot analyses, membranes were cut prior to hybridisation with antibodies. Images of the original blots can be seen in the supplementary material (Supplementary fig. S11–S17).

### Data analysis

All MS RAW files were processed with MaxQuant (version 1.5.4.1.)^30^ against the Uniprot human database (Proteome ID: UP000005640), downloaded on 7 February 2018, containing 71,785 reviewed and unreviewed entries. An MS/MS tolerance (FTMS) of 20 ppm was allowed. Default settings were used and match-between-runs functionality enabled. Carbamidomethylation of cysteine residues was specified as a fixed modification; variable modifications considered were oxidation of methionine and acetylation of protein N-terminus; trypsin was selected as digestion enzyme, with two missed cleavages allowed. Reverse hits to a target-decoy database and common contaminants were removed from the data sets and only protein identifications with a q-value < 0.01 were considered for further analysis. Moreover, protein hits were only considered as candidate interaction partners, if they were not identified in any of the respective isotype control samples but were present in all three replicates of the respective cell line. VennDis JavaFX-based Venn and Euler diagram software created by Ignatchenko et al*.*^57^ was used to generate Venn diagrams for the overlap of identified potential binding partners of Kpnβ1 in cells extracts. Protein–protein interaction networks were analysed using STRING-db (string-db.org), where full STRING networks were drawn (indicating both functional and physical protein associations), and all active interaction sources were selected. A medium confidence level of 0.4 was used. Gene ontology enrichment analysis was carried out using the statistical overrepresentation test of PANTHER (pantherdb.org) and using the Fisher’s exact test to calculate statistical significance. Homo sapiens (all genes in database) was selected as the reference list. For SAINT analysis, proteins were first run through STRINGdb, to increase the SAINT analysis stringency, and then analysed using SAINTexpress_v3.6.3_2018-03-09.

## Supplementary Information

Supplementary Information 1.

Supplementary Table S12.

Supplementary Table S13.

Supplementary Table S14.

Supplementary Table S15.

## Data Availability

The datasets generated during the current study are available in the PRIDE repository, accession number PXD034805.

## Abbreviations

CCAR1: Cell division cycle and apoptosis regulator protein 1
co-IP: Co-immunoprecipitation
FUBP1: Far upstream element-binding protein 1 (FUBP1)
IP-MS: Immunoprecipitation mass spectrometry
Kpnα2: Karyopherin alpha-2
Kpnβ1: Karyopherin beta-1
NPC: Nuclear pore complex
Nup: Nucleoporin
