# Supplementary material for: A mass spectrometry-based approach for the identification of Kpnβ1 binding partners in cancer cells
Source: Sci Rep. 2022 Nov 23;12:20171. doi: 10.1038/s41598-022-24194-6 (PMC9684564; doi:10.1038/s41598-022-24194-6)
Supplement: Supplementary file 1 — Supplementary Information 1. [file 41598_2022_24194_MOESM1_ESM.pdf]

Supplementary Figure 1

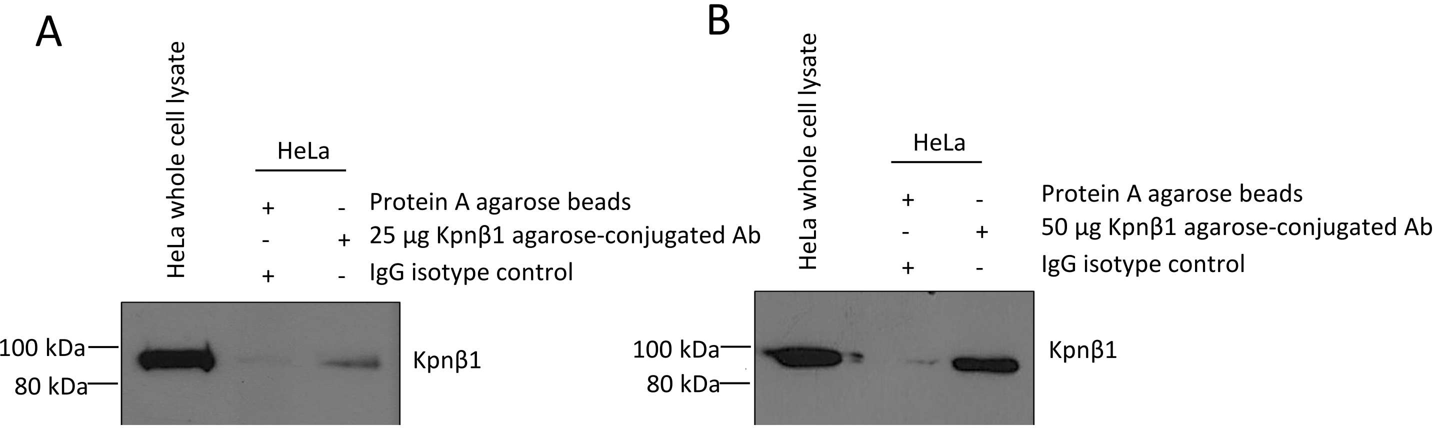

Supplementary figure S1. Optimisation of Kpnβ1 immunoprecipitation. A, B. 25 µg (A) and 50 µg (B) of anti-Karyopherin β1 agarose-conjugated antibody were used to pull-down Kpnβ1 from 500 µg of HeLa cell extracts. Subsequent experiments were carried out using 50 µg antibody.

Supplementary Figure 2

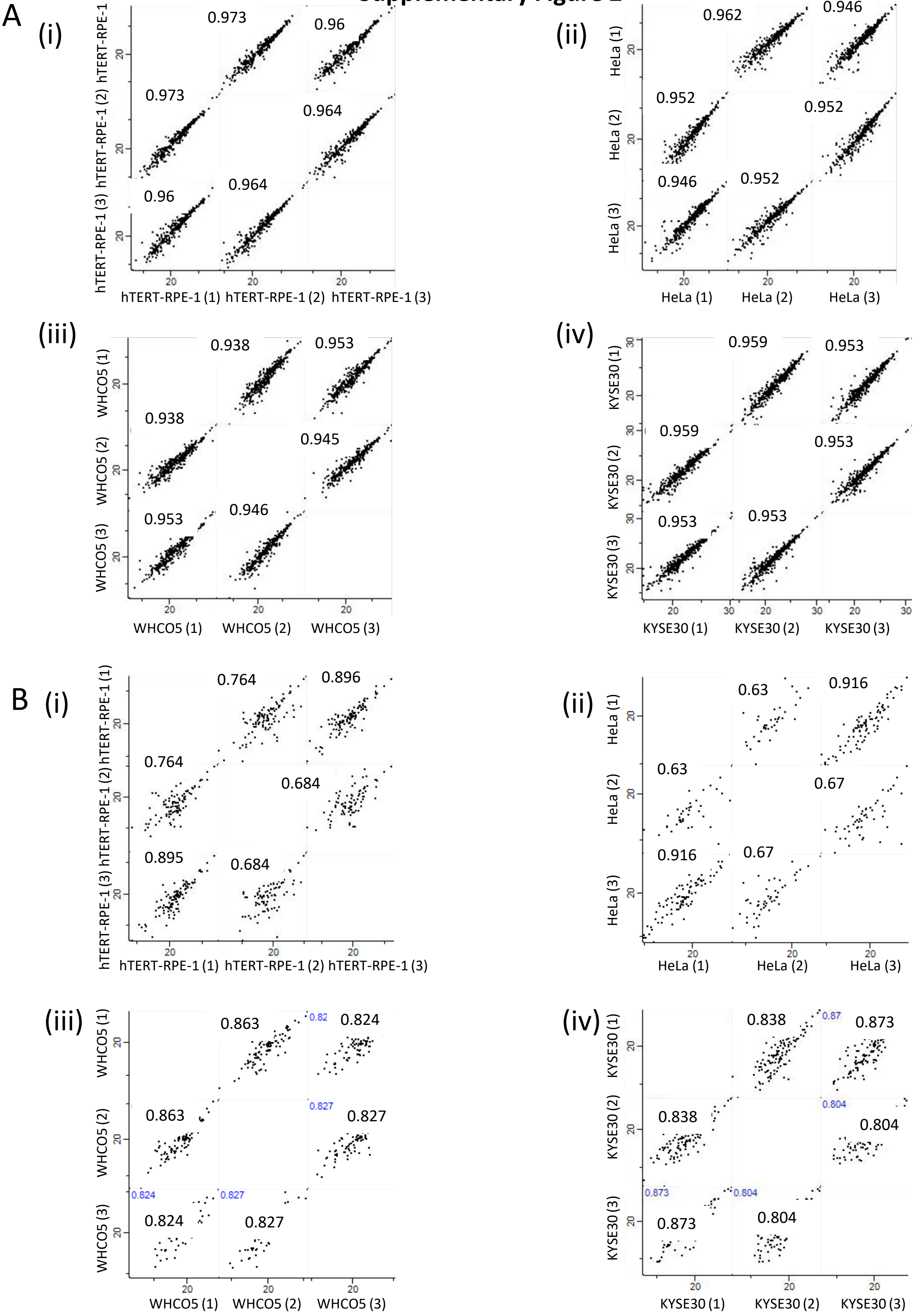

Supplementary figure S2. Multi scatterplots depicting the correlation between replicates samples. A. Scatterplots of log2-transformed iBAQ values of Kpnβ1 pull-down replicates, with Pearson correlation values shown. B. Scatterplots of log2-transformed iBAQ values of IgG isotype control replicates, with Pearson correlation values shown.

Supplementary Figure 3

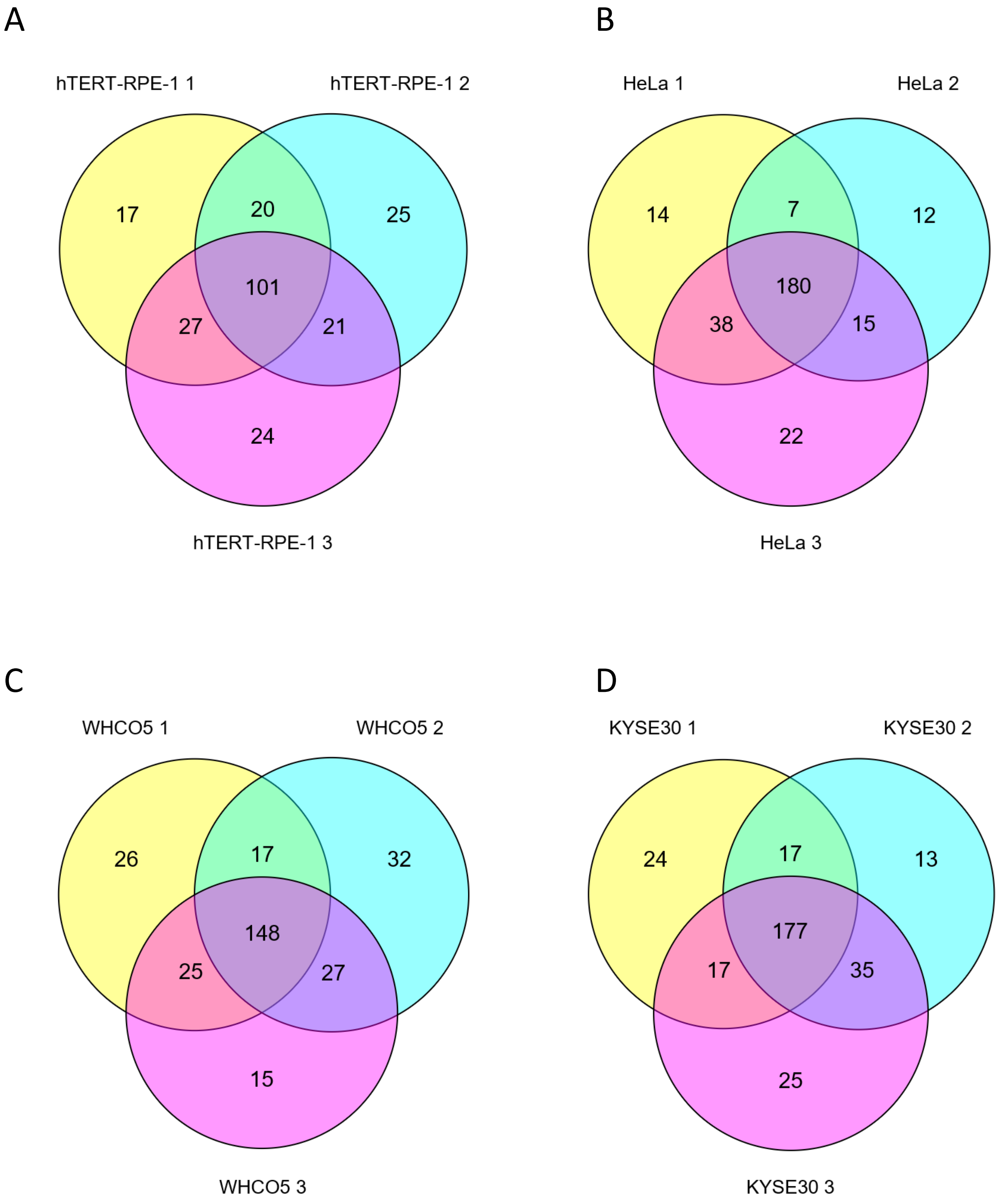

Supplementary figure S3. Venn diagrams showing reproducibility within triplicate samples. Venn diagrams were drawn representing overlaps in binding partners of Kpnβ1 in all three replicates of (A) hTERT-RPE1, (B) HeLa, (C) WHCO5 and (D) KYSE30 cell lines. Lists of proteins were used in which non-specifically bound proteins had been removed.

Supplementary Figure 4

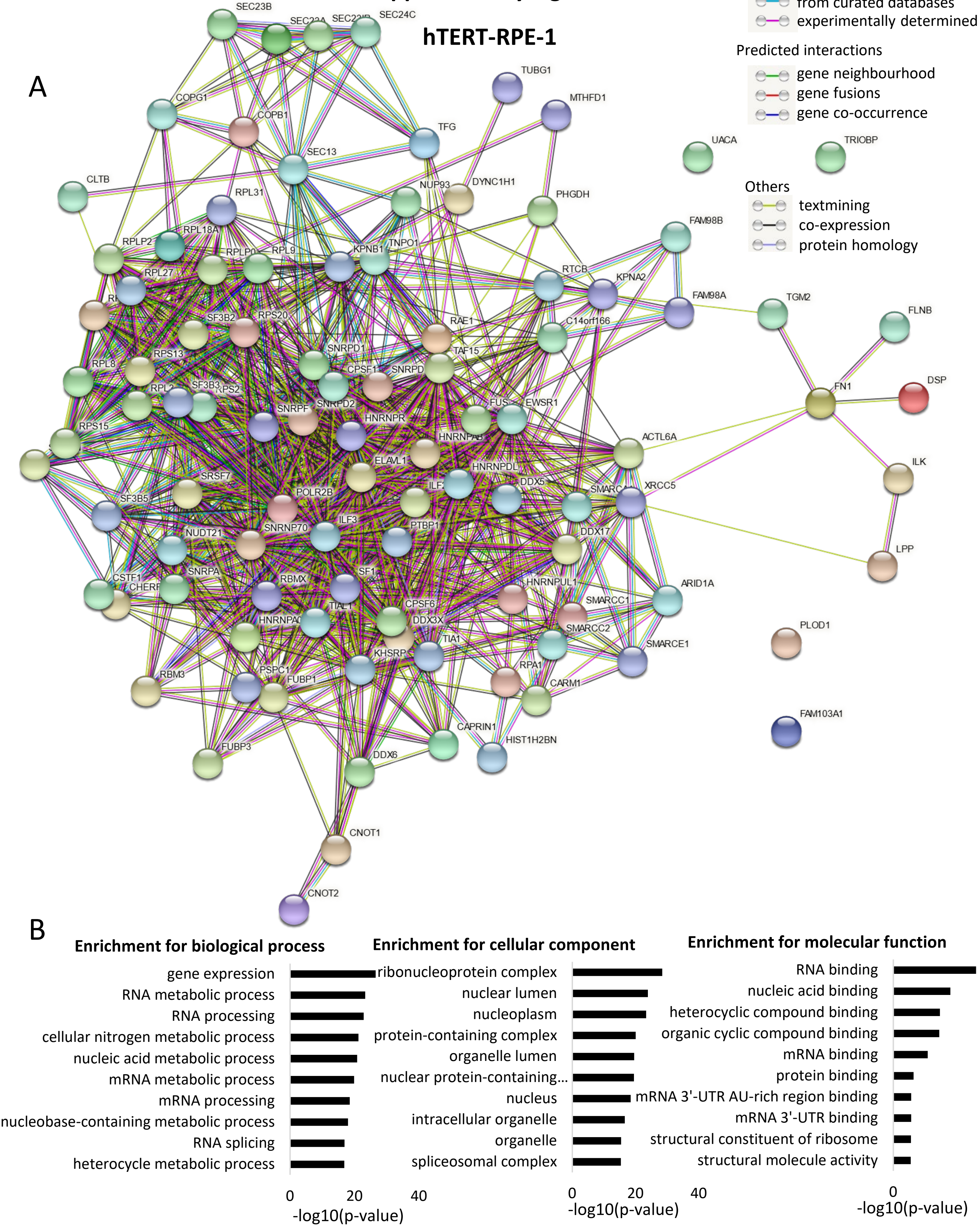

Supplementary figure S4. Protein-protein interaction network and gene ontology analyses of Kpnβ1 binding proteins in hTERT-RPE-1 cells. A. STRING protein-protein interaction (PPI) network analysis of Kpnβ1-interacting proteins in hTERT-RPE-1 cells reveals a high degree of protein-protein interaction. A medium confidence level (0.4) was used. B. PANTHER gene ontology analysis of Kpnβ1-interacting proteins in hTERT-RPE-1 cells. The 10 most significantly enriched biological processes, cellular components and molecular functions are shown.

Supplementary Figure 5

HeLa

A

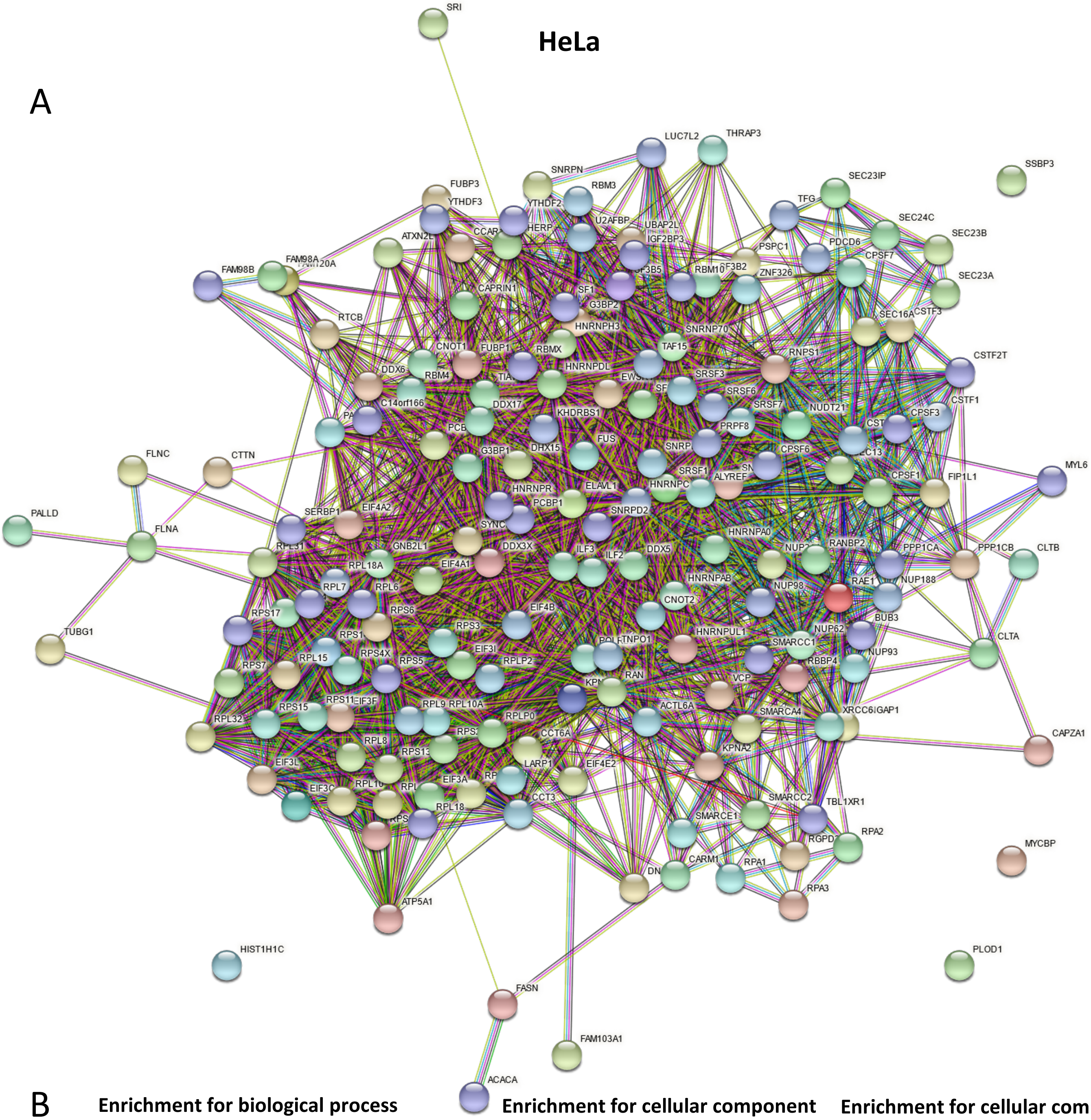

B

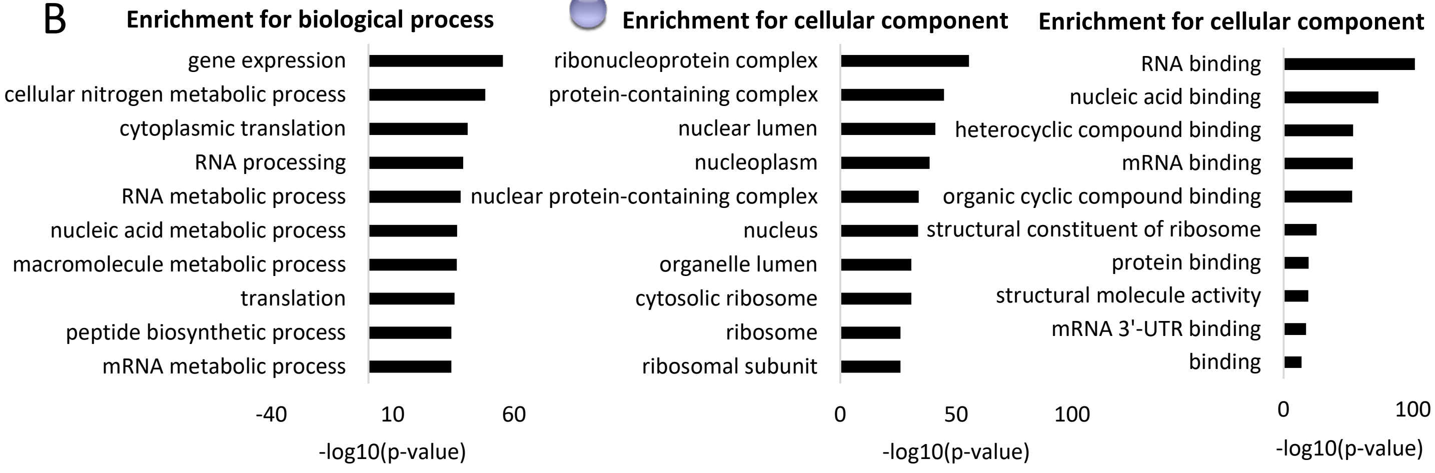

Supplementary figure S5. Protein-protein interaction network and gene ontology analyses of Kpnβ1 binding proteins in HeLa cells. A. STRING protein-protein interaction (PPI) network analysis of Kpnβ1-interacting proteins in HeLa cells reveals a high degree of protein-protein interaction. A medium confidence level (0.4) was used. B. PANTHER gene ontology analysis of Kpnβ1-interacting proteins in HeLa cells. The 10 most significantly enriched biological processes, cellular components and molecular functions are shown.

Supplementary Figure 6

WHCO5

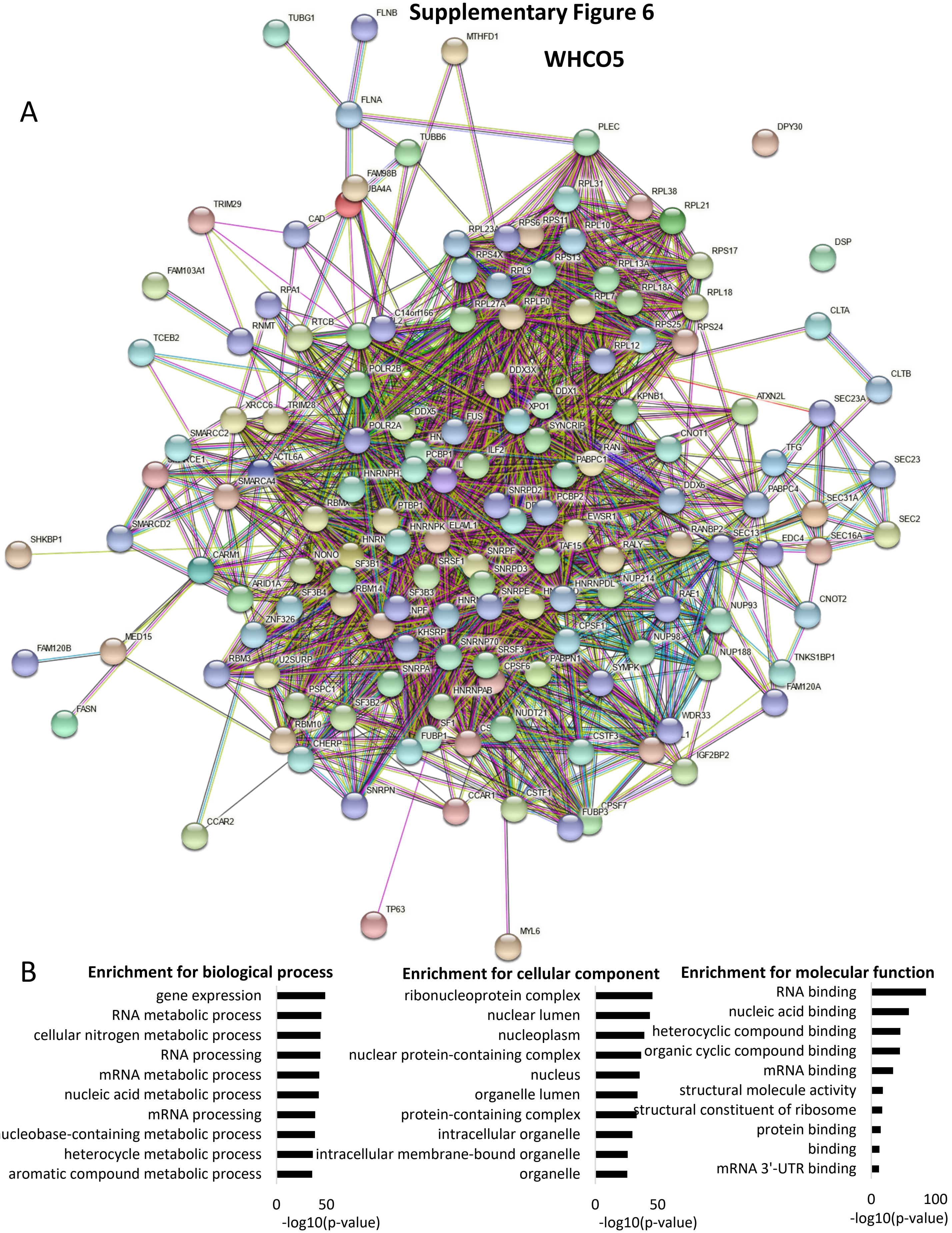

Supplementary figure S6. Protein-protein interaction network and gene ontology analyses of Kpnβ1 binding proteins in WHCO5 cells. A. STRING protein-protein interaction (PPI) network analysis of Kpnβ1-interacting proteins in WHCO5 cells reveals a high degree of protein-protein interaction. A medium confidence level (0.4) was used. B. PANTHER gene ontology analysis of Kpnβ1-interacting proteins in WHCO5 cells. The 10 most significantly enriched biological processes, cellular components and molecular functions are shown.

Supplementary Figure 7

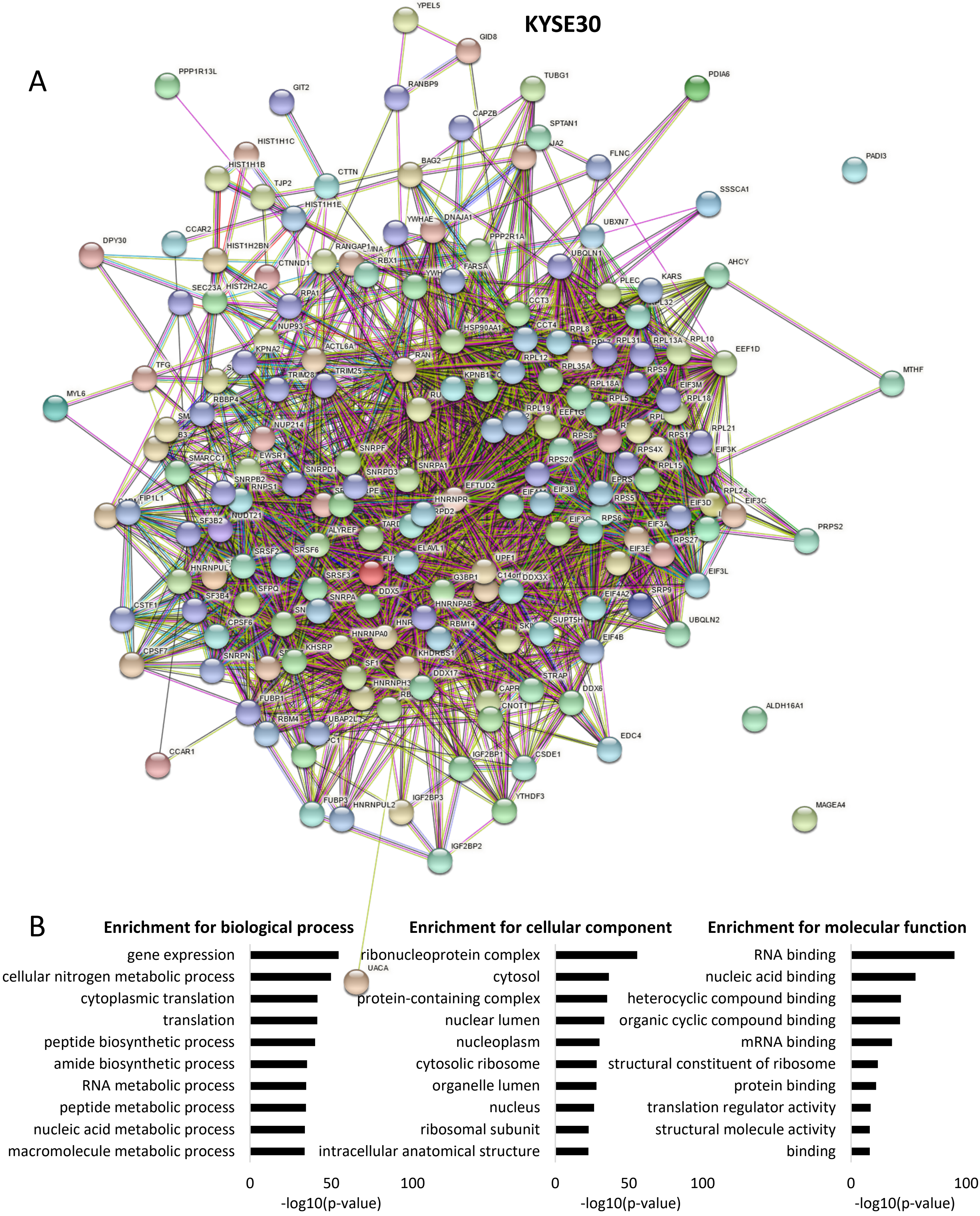

Supplementary figure S7. Protein-protein interaction network and gene ontology analyses of Kpnβ1 binding proteins in KYSE30 cells. A. STRING protein-protein interaction (PPI) network analysis of Kpnβ1-interacting proteins in KYSE30 cells reveals a high degree of protein-protein interaction. A medium confidence level (0.4) was used. B. PANTHER gene ontology analysis of Kpnβ1-interacting proteins in KYSE30 cells. The 10 most significantly enriched biological processes, cellular components and molecular functions are shown.

Supplementary Figure 8

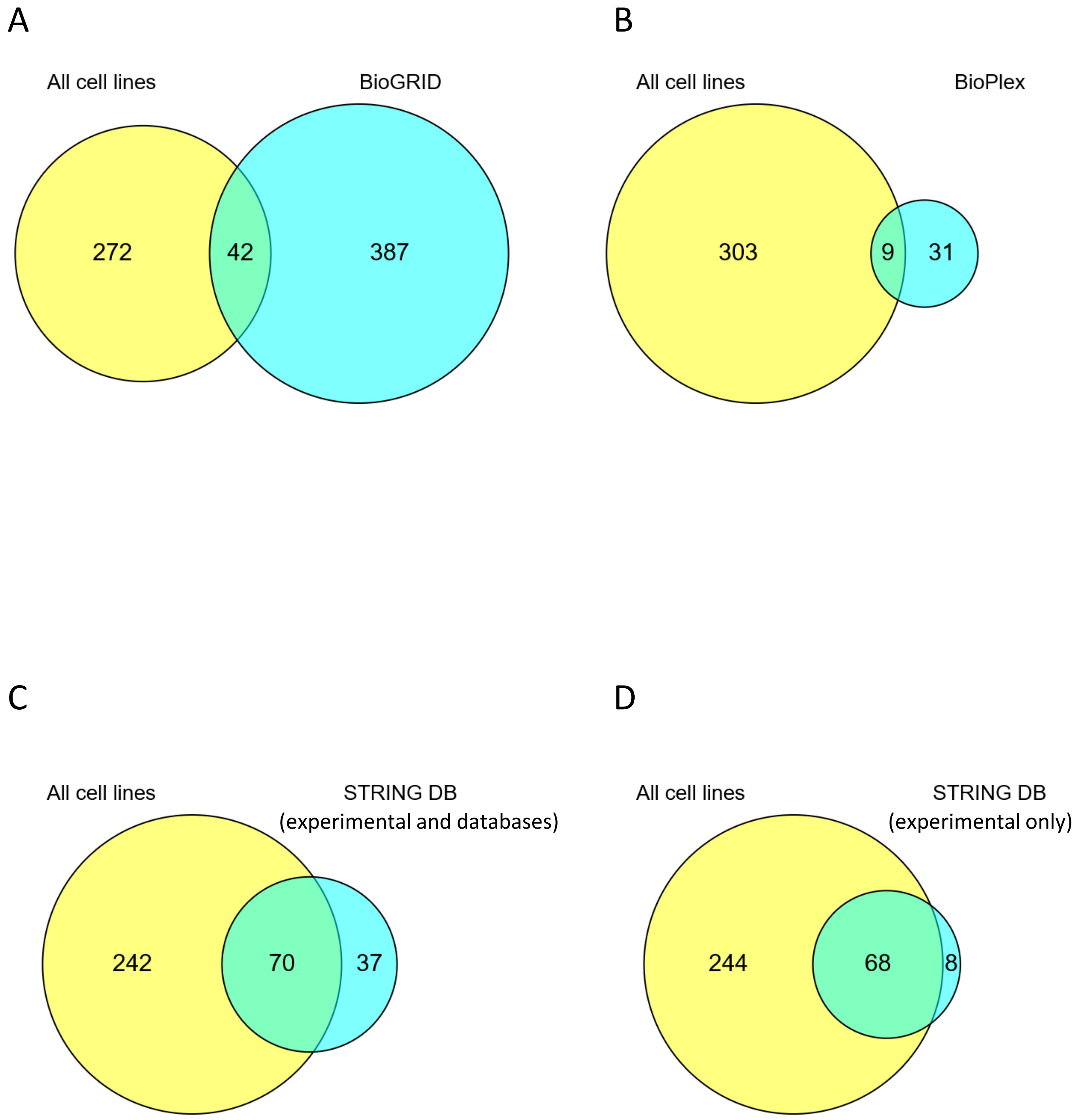

Supplementary figure S8. Venn diagrams showing overlap with other interactome repositories. Venn diagrams were drawn representing overlaps in binding partners of Kpnβ1 in our daaset with BioGRID (A), BioPlex (B) and StringDB (C and D) databases.

Supplementary Figure 9

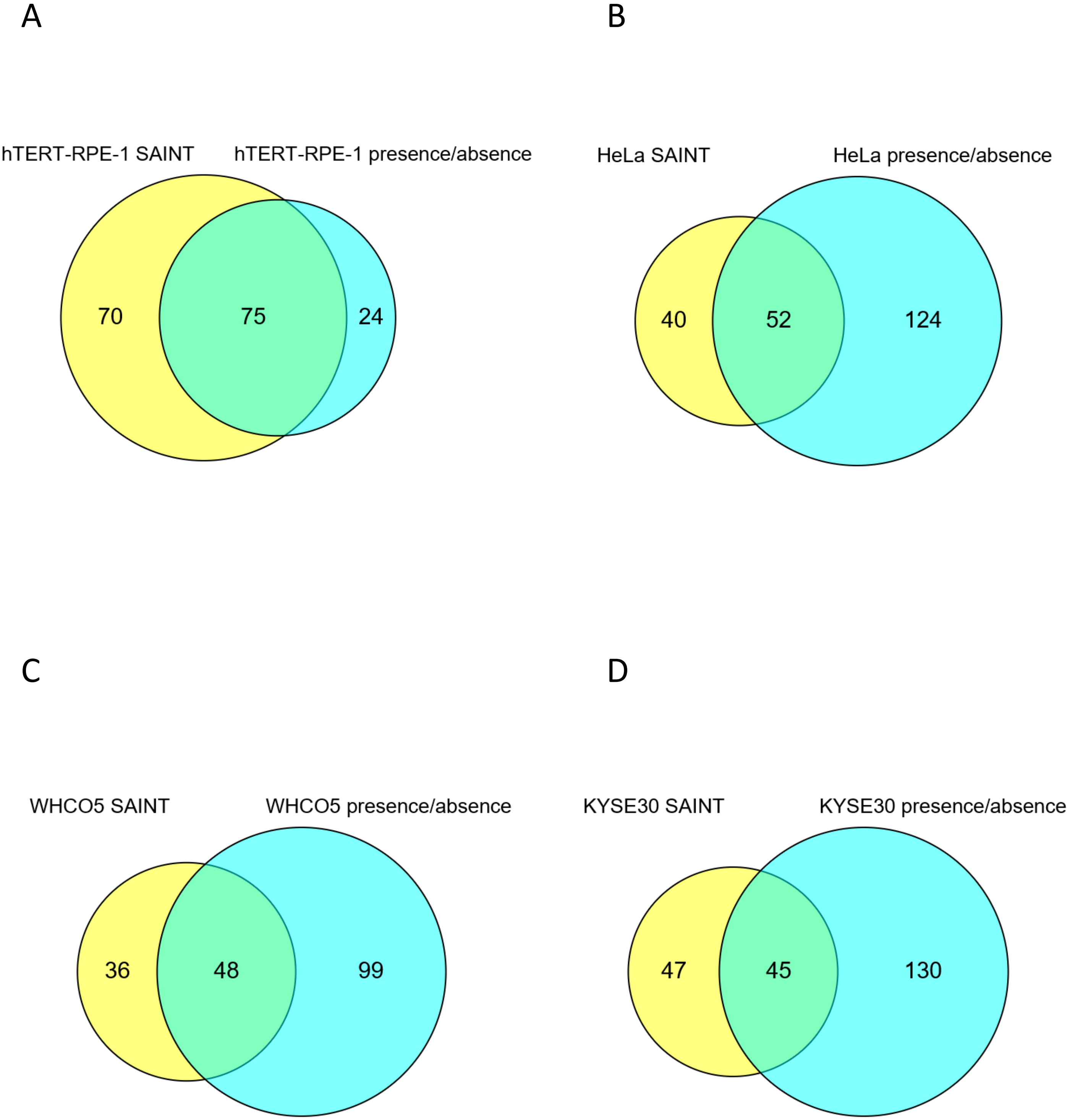

Supplementary figure S9. Venn diagrams showing overlap with SAINT protein lists. Venn diagrams were drawn representing overlaps in binding partners of Kpnβ1 in our daaset with those identified using SAINT, for (A) hTERT-RPE-1, (B) HeLa, (C) WHCO5, and (D) KYSE30.

Supplementary Figure 10

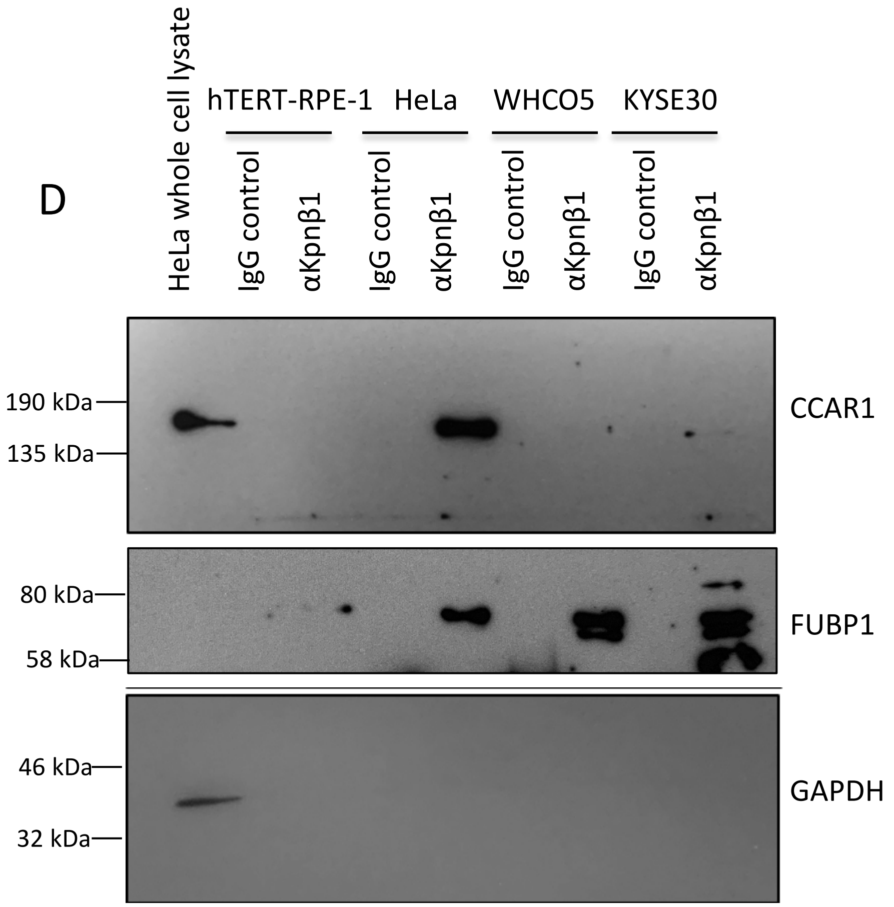

Supplementary figure S10. Validation of Kpnβ1-CCAR1 and Kpnβ1-FUBP1 interaction by Western blot analysis. Western blot analysis from Fig. 5D, showing detection with a more sensitive chemiluminescent substrate, which reveals an interaction between Kpnβ1 and CCAR1 in KYSE30 oesophageal cancer cells and Kpnβ1 and FUBP1 in hTERT-RPE-1 non-cancer cells.

Supplementary Figure 11

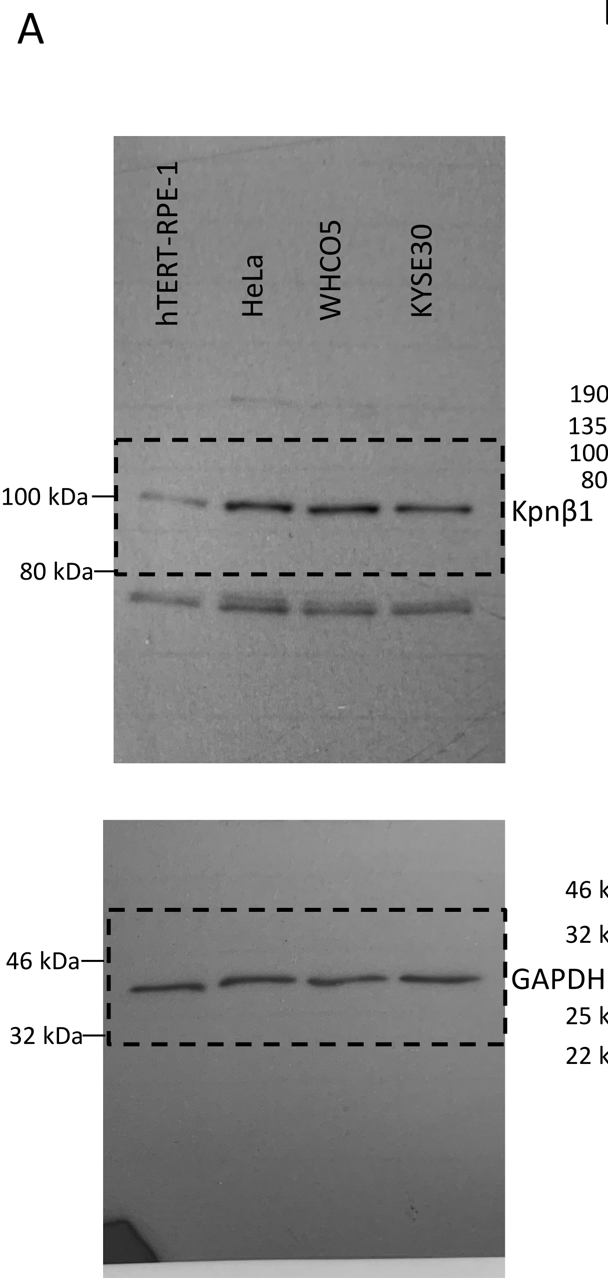

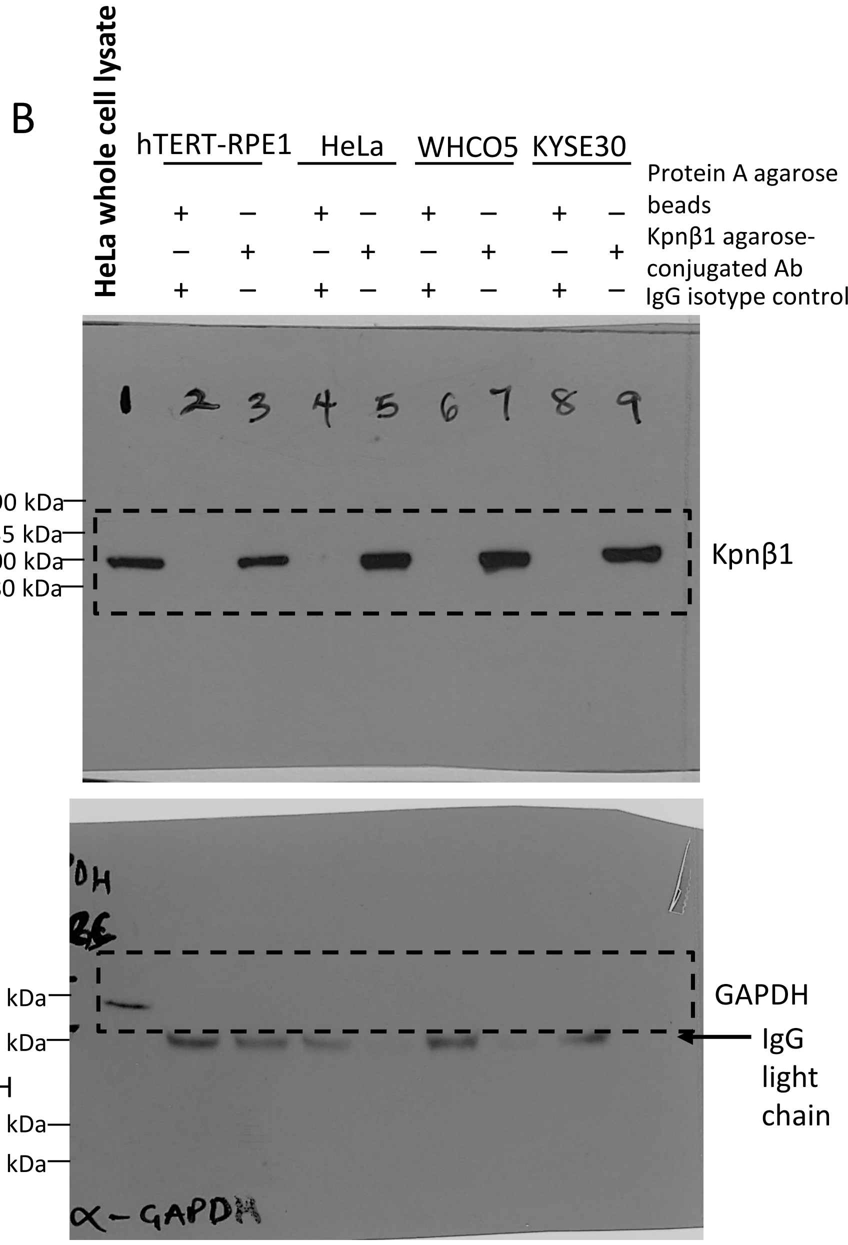

Supplementary figure S11. Uncropped full-length pictures of Western blotting membranes presented in Fig. 1. Membranes were cut to enable blotting for multiple antibodies.

Supplementary Figure 12

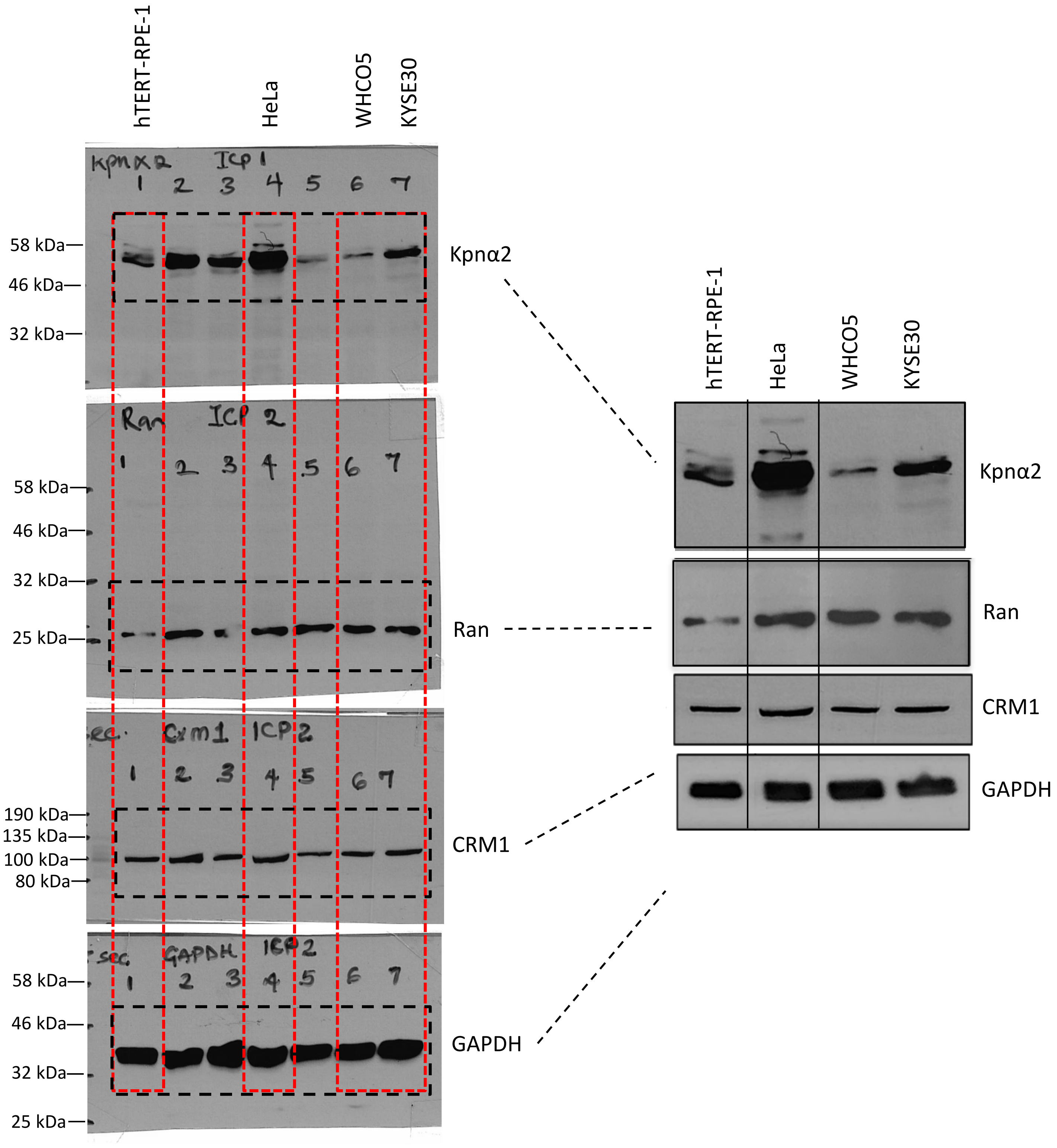

Supplementary figure S12. Uncropped full-length pictures of Western blotting membranes presented in Fig. 5A. Lanes 2, 3 and 5 refer to other cell lines investigated at the start of the study (2: SVWI38; 3: CT-1; 5: CaSki) .

Supplementary Figure 13

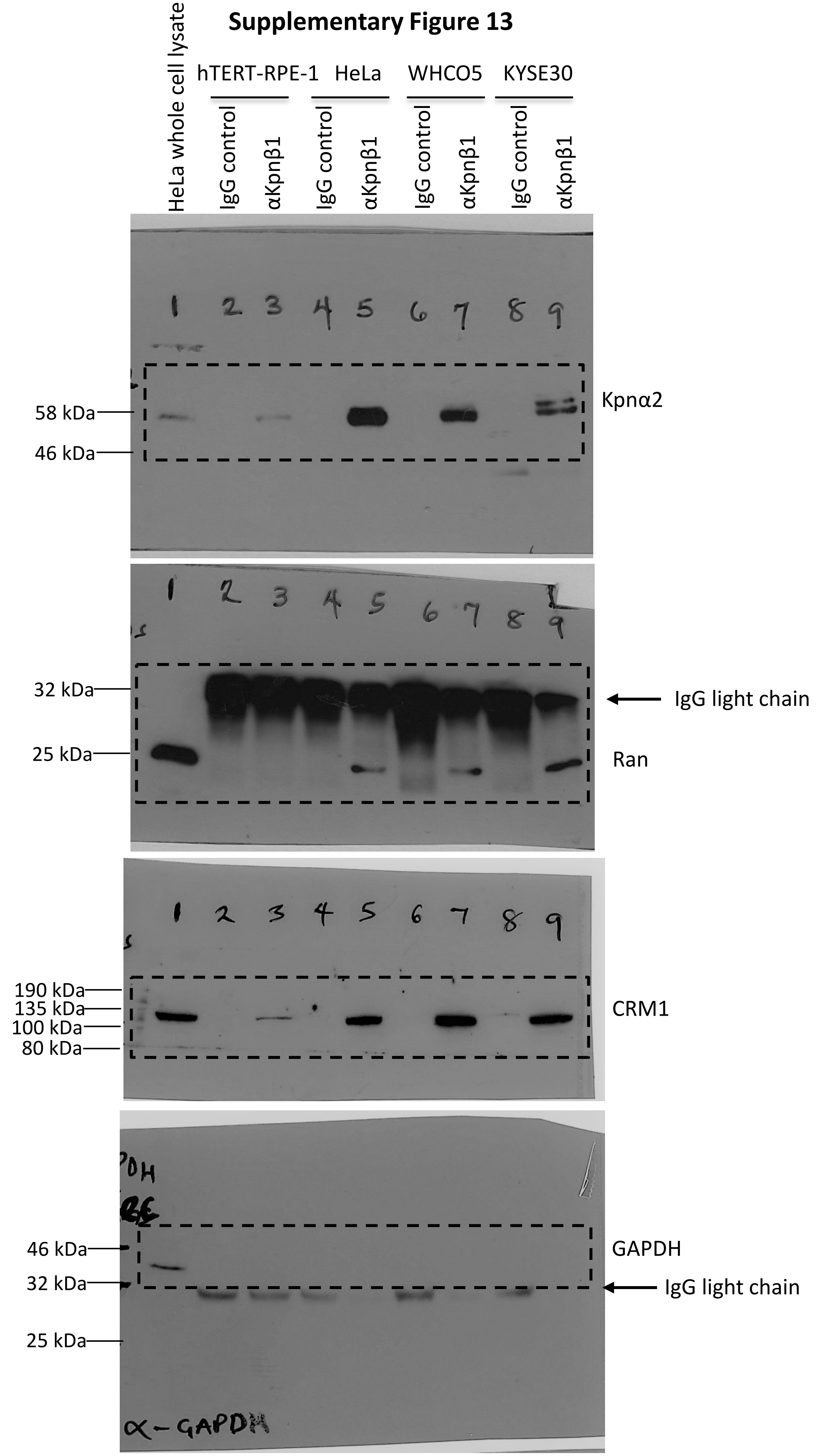

Supplementary figure S13. Uncropped full-length pictures of Western blotting membranes presented in Fig. 5B. Membranes were cut to enable blotting for multiple antibodies.

Supplementary Figure 14

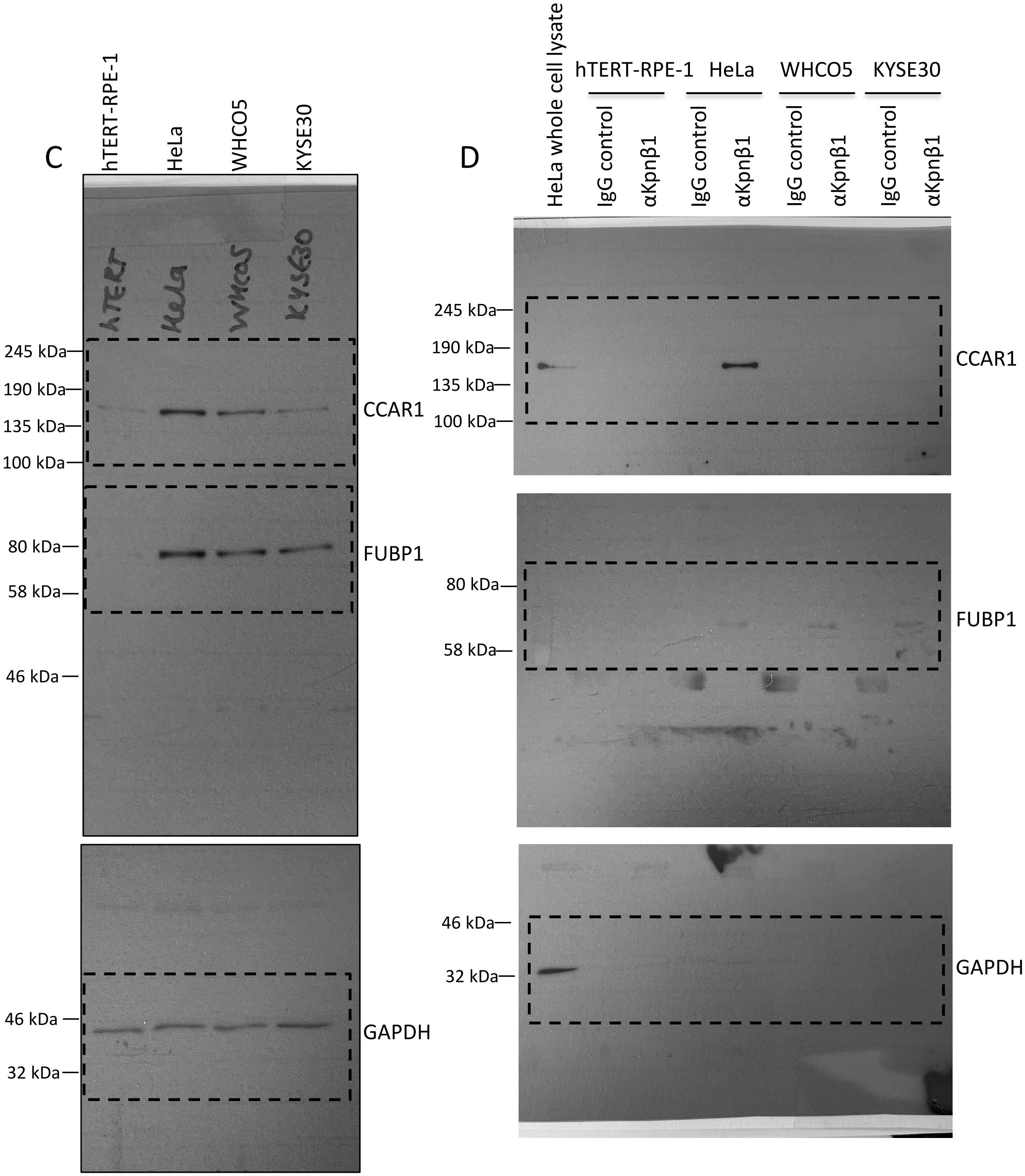

Supplementary figure S14. Uncropped full-length pictures of Western blotting membranes presented in Fig. 5C and D. Membranes were cut to enable blotting for multiple antibodies.

Supplementary Figure 15

A

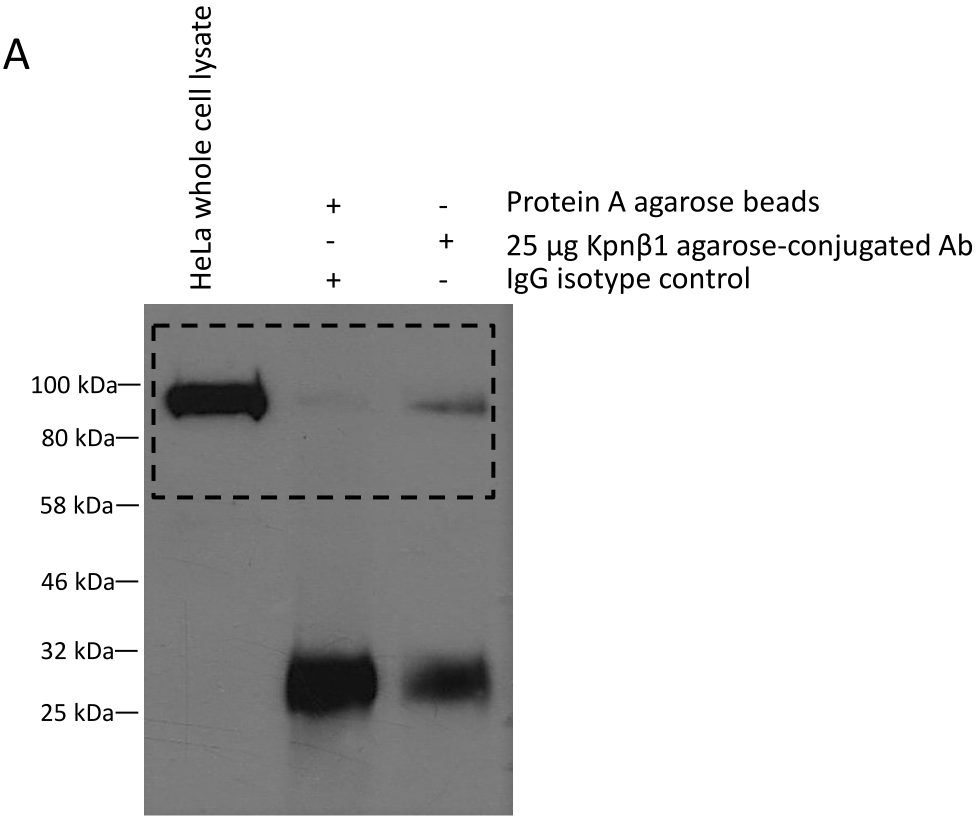

B

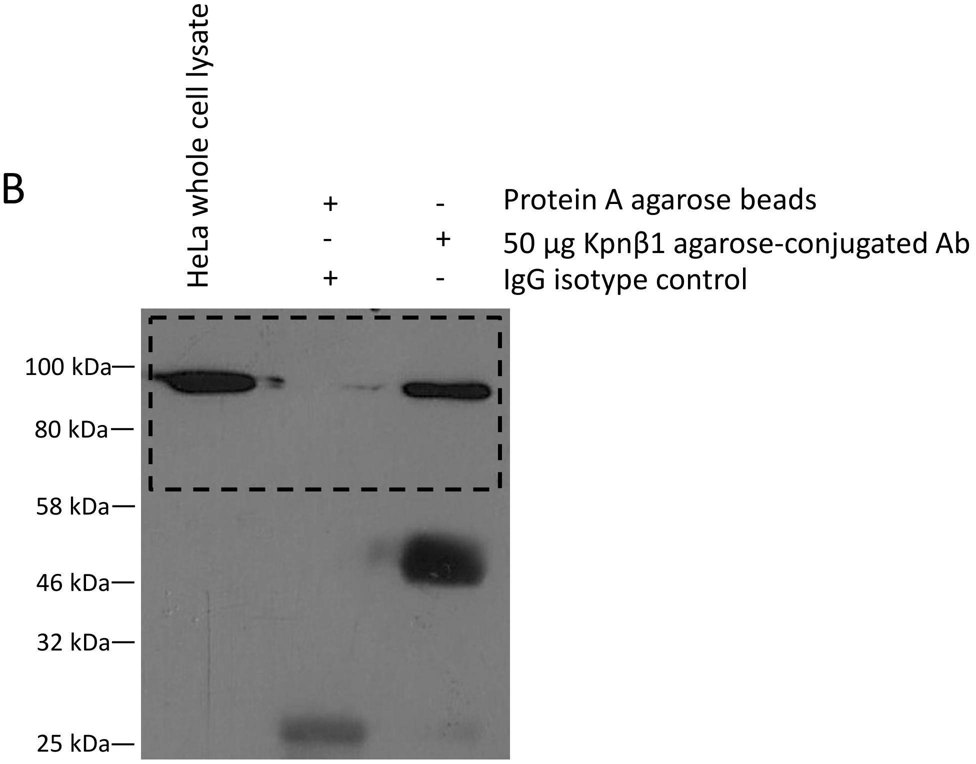

Supplementary figure S15. Uncropped full-length pictures of Western blotting membranes presented in Suppl fig. S1. Membranes were cut to enable blotting for multiple antibodies.

Supplementary Figure 16

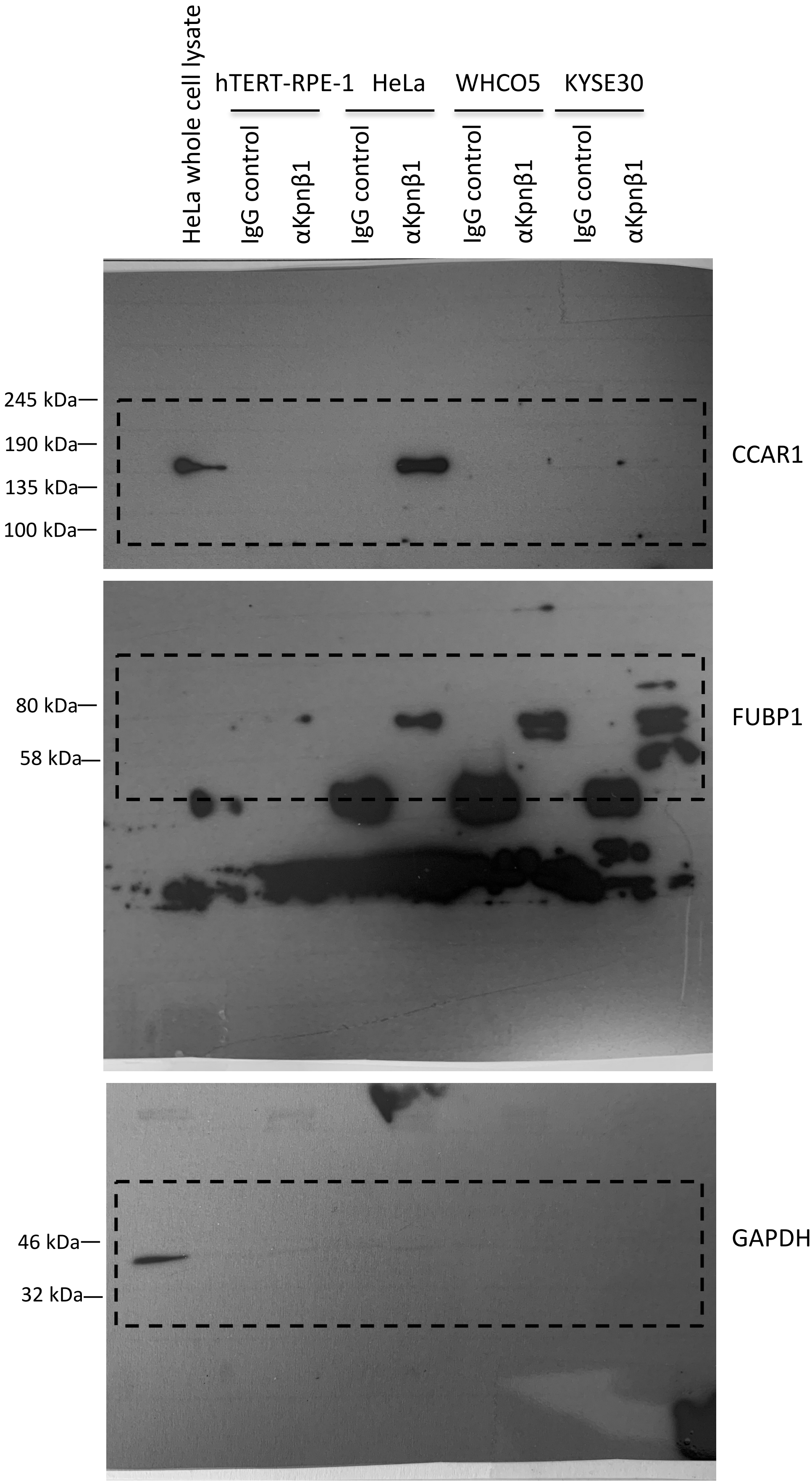

Supplementary figure S16. Uncropped full-length pictures of Western blotting membranes presented in Suppl. fig. S10. Membranes were cut to enable blotting for multiple antibodies.

Supplementary Figure 17

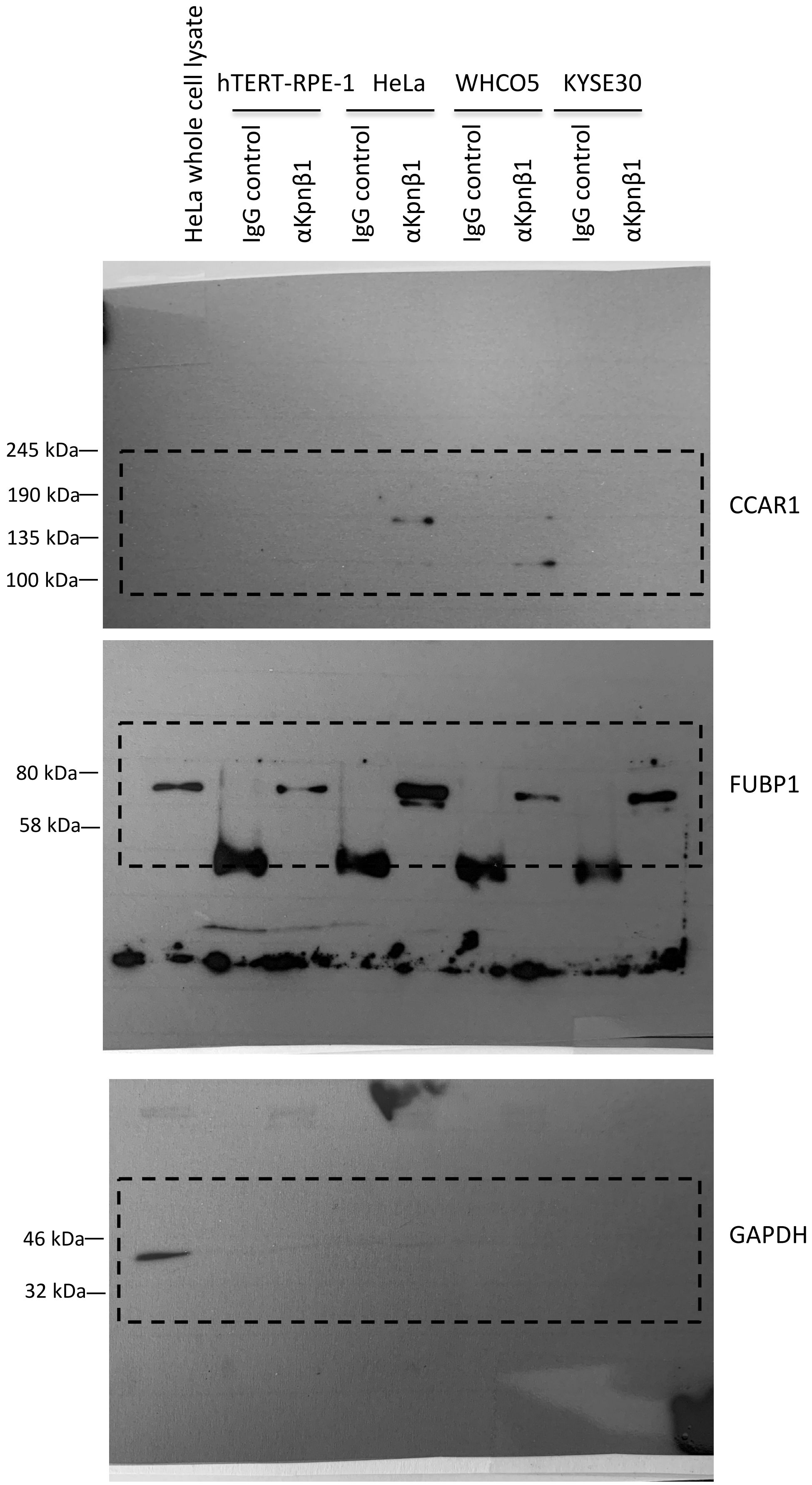

Supplementary figure S17. Uncropped full-length pictures of repeat Western blotting membranes showing pull-down of CCAR1 in HeLa cells and FUBP1 in all of the cell lines.

Supplementary Table 1: List of protein hits identified in IP-MS experiments of Kpnβ1 from hTERT-RPE cell extracts

Gene names	Majority protein IDs	Protein names	Mol. weight [kDa]	Total Peptides	Unique peptides	Total Intensity	Mean intensity	Standard deviation of intensity	Sequence coverage [%]	Score
KPNB1	Q14974	Importin subunit beta-1	97.169	6	6	2.1E+08	69836000	1.7E+07	8.3	45.552
HNRNPDL	A0A087WUK2	Heterogeneous nuclear ribonucleoprotein D-like	6.7215	1	1	28226000	9408667	7160206	31.6	32.233
LPP	A0A087WZF1	Lipoma-preferred partner	63.382	4	4	57318000	19106000	6356521	11.4	28.419
HNRNPUL1	A0A0A0MRA5	Heterogeneous nuclear ribonucleoprotein U-like protein 1	85.939	14	14	6.02E+08	2.01E+08	5006265	24	103.11
SMARCA4	Q9HBD4	Transcription activator BRG1	188.15	5	5	83380000	27793333	1.2E+07	3.6	32.978
ILK	A0A0A0MTH3	Integrin-linked protein kinase	54.611	4	4	31942000	10647067	3642789	9.3	25.752
SRSF7	A0A0B4J1Z1	Serine/arginine-rich splicing factor 7	15.763	3	2	78438000	26146000	3487651	29.2	21.454
DDX3X	A0A0D9SFB3	ATP-dependent RNA helicase DDX3X	70.839	27	26	1.17E+09	3.89E+08	1.2E+08	50.6	247.23
PTBP1	A0A0U1RRM4	Polypyrimidine tract-binding protein 1	62.463	11	11	3.04E+08	97714667	7860571	30.3	83.541
POLR2B	C9J2Y9	DNA-directed RNA polymerase	121.37	7	7	74673000	24891000	1375269	9.7	50.848
DDX17	A0A1X7SBZ2	Probable ATP-dependent RNA helicase DDX17	80.253	25	21	2.1E+09	7.01E+08	7.5E+07	39.5	252.69
PHGDH	A0A286YFA2	D-3-phosphoglycerate dehydrogenase	25.638	4	4	30999000	8603467	622166	19.7	24.151
CNOT1	A5YKK6	CCR4-NOT transcription complex subunit 1	266.94	11	11	1.48E+08	49165667	1.3E+07	5.2	65.446
EWSR1	B0QYK0	RNA-binding protein EWS	64.929	6	6	1.38E+09	4.56E+08	1E+08	13.9	104.02
ILF2	B4DY09	Interleukin enhancer-binding factor 2	38.91	6	6	3.38E+08	1.13E+08	2.4E+07	24.1	58.341
FUBP1	C9JSZ1	Far upstream element-binding protein 1	24.043	10	1	97795000	32598000	4680915	62.5	6.3042
TIA1	C9JTN7	Nucleolysin TIAR	16.794	5	1	36019000	12006333	8820723	34.9	11.699
HNRNPAB	D6R9P3	Heterogeneous nuclear ribonucleoprotein A/B	30.302	6	6	5.56E+08	1.81E+08	1.3E+07	25	50.7
RPL9	D6RAN4	60S ribosomal protein L9	20.775	4	4	1.25E+08	41726000	7974652	28.2	30.838
TIAL1	Q01085	Nucleolysin TIAR	14.616	6	3	4.12E+08	1.37E+08	2.1E+07	55.3	54.853
RPL8	P62917	60S ribosomal protein L8	22.389	3	3	1.25E+08	38477000	9377986	17.1	20.347
SF3B2	Q13435	Splicing factor 3B subunit 2	98.169	9	9	2.55E+08	84088333	1.9E+07	17.2	76.397
RAE1	P78406	mRNA export factor	47.831	5	5	1.63E+08	53066667	1E+07	22	39.994

UACA	F5H2B9	Uveal autoantigen with coiled-coil domains and ankyrin repeats	150.53	8	8	1.12E+08	37464000	8509298	7.8	56.664
MTHFD1	F5H2F4	C-1-tetrahydrofolate synthase, cytoplasmic	110.61	7	7	89042000	29368333	4951522	8.2	42.425
SEC23A	F5H365	Protein transport protein Sec23A	82.968	19	17	1.23E+09	4.09E+08	9.3E+07	36.1	323.31
RPLP0	P05388	60S acidic ribosomal protein P0	15.813	3	3	90313000	29030000	2754266	27.5	25.876
CNOT2	F8VV52	CCR4-NOT transcription complex subunit 2	58.62	5	5	1.21E+08	39592333	4315835	12.4	32.169
TIA1	F8W8I6	Nucleolysin TIAR	16.794	5	1	36019000	12006333	8820723	34.9	11.699
CPSF6	F8WJN3	Cleavage and polyadenylation specificity factor subunit 6	52.269	4	4	1.99E+08	66246667	2.5E+07	9.4	31.697
SEC24C	G5EA31	Protein transport protein Sec24C	111.98	17	17	7.18E+08	2.38E+08	4.5E+07	24.3	151.81
RBMX	P38159	RNA-binding motif protein, X chromosome	31.745	4	4	1.16E+08	38804000	3587592	16.8	36.65
NUP93	Q8N1F7	Nuclear pore complex protein Nup93	99.554	6	6	49972000	16657333	4903131	10.5	40.865
DDX5	J3KTA4	Probable ATP-dependent RNA helicase DDX5	69.086	10	6	3.14E+08	1.05E+08	7519483	17.6	44.233
CHERP	J3QK89	Calcium homeostasis endoplasmic reticulum protein	104.93	5	5	86424000	28808333	1.6E+07	7.3	32.55
SNRPN	J3QLE5	Small nuclear ribonucleoprotein-associated protein N	17.546	6	6	5.15E+08	1.68E+08	3.7E+07	26.6	54.026
RPS15	K7ELC2	40S ribosomal protein S15	17.723	3	3	29079000	9170667	6483154	44.7	27.452
RPL18A	M0R3D6	60S ribosomal protein L18a	16.714	4	4	90888000	29716667	9803119	24.1	24.158
SNRPA	P09012	U1 small nuclear ribonucleoprotein A	28.388	2	2	99423000	33141333	5066377	19.1	20.171
ARID1A	O14497	AT-rich interactive domain-containing protein 1A	242.04	12	12	1.8E+08	60042667	1.7E+07	7.4	75.102
HNRNPR	O43390	Heterogeneous nuclear ribonucleoprotein R	70.942	9	6	1.79E+08	59750333	1.4E+07	18	38.955
NUDT21	O43809	Cleavage and polyadenylation specificity factor subunit 5	26.227	7	7	2.97E+08	96824333	2.8E+07	39.6	55.088
HIST1H2BN	U3KQK0	Histone H2B	18.804	6	2	2.4E+08	78630667	4.8E+07	31.3	68.659
FLNB	O75369	Filamin-B	278.16	12	5	36638000	12212867	6700144	4.4	32.522
ACTL6A	O96019	Actin-like protein 6A	47.46	7	7	1.91E+08	63634000	1.9E+07	26.8	50.796
FN1	P02751	Fibronectin	262.62	19	19	7.47E+08	2.49E+08	5.2E+07	11.9	194.35
RPLP2	P05387	60S acidic ribosomal protein P2	11.665	2	2	1.06E+08	33661000	4196025	24.3	21.395

SNRNP70	P08621	U1 small nuclear ribonucleoprotein 70 kDa	51.556	5	5	1.54E+08	51328333	5778532	16	46.723
CLTB	P09497	Clathrin light chain B	25.19	5	5	2.46E+08	81956333	4.4E+07	14.8	30.58
XRCC5	P13010	X-ray repair cross-complementing protein 5	82.704	7	7	76290000	25430033	1.4E+07	19.7	45.729
RPS2	P15880	40S ribosomal protein S2	31.324	5	5	3.24E+08	1.07E+08	2.1E+07	18.8	31.549
DSP	P15924	Desmoplakin	331.77	13	13	1.49E+08	49658667	2.2E+07	5.5	96.576
TGM2	P21980	Protein-glutamine gamma-glutamyltransferase 2	77.328	12	12	8.31E+08	2.76E+08	5.1E+07	23.3	95.152
TUBG1	P23258	Tubulin gamma-1 chain	51.169	6	6	1.72E+08	56866667	6445884	18.4	47.895
DDX6	P26196	Probable ATP-dependent RNA helicase DDX6	54.416	11	11	3.15E+08	1.05E+08	1.8E+07	35.6	122.28
RPA1	P27694	Replication protein A 70 kDa DNA-binding subunit	68.137	5	5	80559000	26853000	3152666	9.9	29.946
FUS	P35637	RNA-binding protein FUS	53.425	11	9	2.58E+09	8.46E+08	1.6E+08	20.2	142.88
RPL3	P39023	60S ribosomal protein L3	46.108	5	5	2.06E+08	68662667	1.5E+07	17.9	38.42
KPNA2	P52292	Importin subunit alpha-1	57.861	4	4	75556000	25185333	4487411	12.3	56.088
COPB1	P53618	Coatomer subunit beta	107.14	5	5	68544000	22847667	7720211	8.8	38.202
SEC13	P55735	Protein SEC13 homolog	35.54	5	5	9.9E+08	3.23E+08	6.6E+07	23	139.67
RPS20	P60866	40S ribosomal protein S20	13.373	2	2	90225000	26537000	3270229	22.7	17.474
RPL27	P61353	60S ribosomal protein L27	15.798	6	6	2.26E+08	72444667	9623767	45.6	38.703
RPS8	Q5JR95	40S ribosomal protein S8	21.879	5	5	2.8E+08	93222000	1.8E+07	29.3	43.431
RPS13	P62277	40S ribosomal protein S13	17.222	3	3	1.05E+08	35110667	4701802	21.2	18.167
SNRPF	P62306	Small nuclear ribonucleoprotein F	9.7251	2	2	2.09E+08	68366000	2E+07	39.5	23.585
SNRPD1	P62314	Small nuclear ribonucleoprotein Sm D1	13.281	4	4	2.46E+08	78058000	1.9E+07	54.6	37.388
SNRPD2	P62316	Small nuclear ribonucleoprotein Sm D2	13.527	4	4	1.45E+08	48430000	1.2E+07	30.5	61.619
SNRPD3	P62318	Small nuclear ribonucleoprotein Sm D3	13.916	2	2	4.27E+08	1.37E+08	1.5E+07	15.1	17.834
RPL31	P62899	60S ribosomal protein L31	14.463	3	3	1.42E+08	47402667	5179142	26.4	23.504
RBM3	P98179	RNA-binding protein 3	17.17	4	4	1.82E+08	60786000	1.6E+07	47.8	30.426
PLOD1	Q02809	Procollagen-lysine	83.549	11	11	1.87E+08	60607667	2.1E+07	22.1	87.323
CSTF1	Q05048	Cleavage stimulation factor subunit 1	48.357	6	6	1.87E+08	62300333	2282239	20.6	54.508
CPSF1	Q10570	Cleavage and polyadenylation specificity factor subunit 1	160.88	5	5	39124000	13041333	378987	3.8	40.954

ILF3	Q12906	Interleukin enhancer-binding factor 3	95.337	10	10	2.44E+08	81303333	2.2E+07	16.7	100.71
HNRNPA0	Q13151	Heterogeneous nuclear ribonucleoprotein A0	30.84	6	6	4.11E+08	1.37E+08	2.1E+07	23.6	40.748
DYNC1H1	Q14204	Cytoplasmic dynein 1 heavy chain 1	532.4	15	15	2.29E+08	76403667	1E+07	4.4	97.047
CAPRIN1	Q14444	Caprin-1	78.365	6	6	1.78E+08	59389000	2.1E+07	15.7	43.488
SF3B3	Q15393	Splicing factor 3B subunit 3	135.58	11	11	4.54E+08	1.5E+08	7E+07	13	108.29
SEC23B	Q15437	Protein transport protein Sec23B	86.478	6	4	38516000	12838333	1526171	10.6	48.549
SF1	Q15637	Splicing factor 1	68.329	8	8	2.92E+08	97422667	2.8E+07	20.7	73.775
ELAVL1	Q15717	ELAV-like protein 1	36.091	5	5	1.43E+08	46836333	9640707	21.8	40.345
FAM98B	Q52LJ0	Protein FAM98B	37.19	6	5	2.57E+08	85541667	2.4E+07	24.2	131.83
CARM1	Q86X55	Histone-arginine methyltransferase CARM1	65.853	9	9	4.03E+08	1.34E+08	2.5E+07	24.8	69.129
FAM98A	Q8NCA5	Protein FAM98A	55.4	5	4	2.34E+08	78113000	1E+07	15.8	35.894
SMARCC2	Q8TAQ2	SWI/SNF complex subunit SMARCC2	132.88	9	5	2.66E+08	88562000	3.4E+07	11.9	125.11
PSPC1	Q8WXF1	Paraspeckle component 1	58.743	12	11	4.22E+08	1.41E+08	2.8E+07	27.5	86.703
TFG	Q92734	Protein TFG	43.447	11	11	1.68E+09	5.59E+08	1.1E+08	38	312.34
TAF15	Q92804	TATA-binding protein-associated factor 2N	61.829	5	3	1.62E+08	54085667	5463317	15.5	22.338
SMARCC1	Q92922	SWI/SNF complex subunit SMARCC1	122.87	5	1	36543000	12181167	1792774	4.9	13.685
KHSRP	Q92945	Far upstream element-binding protein 2	73.114	29	27	4.91E+09	1.63E+09	2.3E+08	51.9	323.31
TNPO1	Q92973	Transportin-1	102.35	7	7	1.26E+08	42061333	1.6E+07	10.2	42.502
SMARCE1	Q969G3	SWI/SNF-related matrix-associated actin-dependent regulator of chromatin subfamily E member 1	46.649	4	4	1.31E+08	43810000	1.3E+07	18.5	39.988
FUBP3	Q96I24	Far upstream element-binding protein 3	61.64	12	11	3.92E+08	1.31E+08	9749472	34.1	89.592
FAM103A1	Q9BTL3	RNMT-activating mini protein	14.381	2	2	1.03E+08	34487333	1661735	23.7	14.568
SF3B5	Q9BWJ5	Splicing factor 3B subunit 5	10.135	2	2	69418000	23139333	836030	25.6	19.897
TRIOBP	Q9H2D6	TRIO and F-actin-binding protein	261.37	5	5	1.15E+08	38394333	1.5E+07	2.7	63.453
C14orf166	Q9Y224	UPF0568 protein C14orf166	28.068	9	9	1.31E+09	4.34E+08	5.8E+07	41.8	140.04
RTCB	Q9Y3I0	tRNA-splicing ligase RtcB homolog	55.21	20	20	2.66E+09	8.86E+08	9.2E+07	49.5	216.51
COPG1	Q9Y678	Coatomer subunit gamma-1	97.717	2	2	46360000	15453367	6871131	2.6	12.541
SEC23IP	Q9Y6Y8	SEC23-interacting protein	111.08	14	14	6.44E+08	2.15E+08	5.6E+07	20.9	129.85

Supplementary Table 2: List of protein hits identified in IP-MS experiments of Kpnβ1 from HeLa cell extracts

Gene names	Majority protein IDs	Protein names	Mol. weight [kDa]	Total Peptides	Unique peptides	Total Intensity	Mean intensity	Standard deviation of intensity	Sequence coverage [%]	Score
KPNB1	Q14974	Importin subunit beta-1	97.169	11	11	3.75E+08	1.25E+08	59496420	17.1	109.62
RPS9	A0A024R4M0	40S ribosomal protein S9	22.591	4	4	1.19E+08	39698000	12125353	17.5	23.457
YTHDF3	A0A024R7W5	YTH domain-containing family protein 3	58.311	4	3	1.3E+08	43285000	14152202	10.7	32.34
TAF15	Q92804	TATA-binding protein-associated factor 2N	48.839	4	2	1.27E+08	42325333	30125195	14.5	26.82
HNRNPDL	A0A087WUK2	Heterogeneous nuclear ribonucleoprotein D-like	40.04	7	6	6.24E+08	2.08E+08	39531763	17.9	66.24
MYCBP	A0A087WV05	C-Myc-binding protein	12.755	3	3	1.21E+08	40208333	19187056	41.8	20.741
RPL10	P27635	60S ribosomal protein L10	18.565	5	5	2.1E+08	70070667	16200801	25.2	43.631
SSBP3	A0A087WVT6	Single-stranded DNA-binding protein 3	37.772	3	3	1.1E+08	36647000	5276880	11.9	30.001
SRSF3	A0A087X2D0	Serine/arginine-rich splicing factor 3	10.32	4	3	2.49E+08	83150367	66713504	42.1	39.063
RBM10	A0A0A0MR66	RNA-binding protein 10	110.36	7	7	1.13E+08	37516000	13675802	8.3	51.44
HNRNPUL1	A0A0A0MRA5	Heterogeneous nuclear ribonucleoprotein U-like protein 1	85.939	15	15	7.54E+08	2.51E+08	54894608	26.1	132.27
CSTF1	Q05048	Cleavage stimulation factor subunit 1	38.448	6	6	2.17E+08	72397000	31872267	27.2	122.61
SMARCA4	Q9HBD4	Transcription activator BRG1	188.15	11	11	1.59E+08	53140333	22832397	8.2	96.588
SRSF7	A0A0B4J1Z1	Serine/arginine-rich splicing factor 7	15.763	3	2	1.09E+08	36283667	16599373	29.2	17.414
LARP1	A0A0B4J210	La-related protein 1	69.746	4	4	91249000	30416333	21479112	8	26.034
DDX3X	A0A0D9SFB3	ATP-dependent RNA helicase DDX3X;ATP-dependent RNA helicase DDX3Y	70.839	23	23	1.49E+09	4.96E+08	1.66E+08	46.2	268.18
TBL1XR1	A0A0D9SF63	F-box-like/WD repeat-containing protein TBL1XR1	51.556	4	4	1.72E+08	57410667	16075955	14.3	84.914
FASN	A0A0U1RQF0	Fatty acid synthase	273.2	19	19	2.7E+08	90134333	25816845	10.9	185.17
POLR2B	C9J2Y9	DNA-directed RNA polymerase	121.37	9	9	1.88E+08	62570000	22613572	11.5	64.947
DDX17	A0A1X7SBZ2	Probable ATP-dependent RNA helicase DDX17	80.253	23	19	2.89E+09	9.64E+08	3.11E+08	37	307.02
CNOT1	A5YKK6	CCR4-NOT transcription complex subunit 1	266.94	13	13	2.32E+08	77344000	41006501	6.1	82.884

RGPD3	J3KNE0	RanBP2-like and GRIP domain-containing protein 3	197.62	4	1	31087000	10362667	3666462	2.4	12.868
EIF3L	B0QY90	Eukaryotic translation initiation factor 3 subunit L	55.161	2	2	19102000	6367267	5300612	4.9	25.729
EWSR1	B0QYK0	RNA-binding protein EWS	64.929	6	6	9.04E+08	3E+08	1.09E+08	13.9	94.219
XRCC6	B1AHC9	X-ray repair cross-complementing protein 6	64.283	10	10	2.32E+08	75526667	20506466	25.4	85.374
PABPC4	B1ANR0	Polyadenylate-binding protein	67.97	14	7	4.36E+08	1.45E+08	50967048	25.2	51.772
HNRNPC	G3V555	Heterogeneous nuclear ribonucleoproteins C1/C2	28.916	8	8	1.35E+09	4.39E+08	1.26E+08	33.2	119.18
ILF2	B4DY09	Interleukin enhancer-binding factor 2	38.91	9	9	4.92E+08	1.63E+08	60694993	42.3	68.465
RAN	B5MDF5	GTP-binding nuclear protein Ran	26.224	4	4	1.72E+08	57365333	12019179	21.9	38.535
EIF3C	Q99613	Eukaryotic translation initiation factor 3 subunit C	105.34	3	3	54462000	18154367	7601887	4.2	32.858
MYL6	F8W1R7	Myosin light polypeptide 6	14.436	3	3	1.02E+08	34102000	14533680	33.8	21.857
EIF4E2	B8ZZL3	Eukaryotic translation initiation factor 4E type 2	23.248	2	2	32266000	10755500	3300394	11.5	13.821
SRI	C9J0K6	Sorcin	17.605	3	3	74773000	24924333	6926704	19.4	17.197
FUBP1	C9JSZ1	Far upstream element-binding protein 1	24.043	11	1	1.35E+08	45064000	11503995	65.5	7.7765
TIA1	C9JTN7	Nucleolysin TIA-1 isoform p40	42.835	3	1	15533000	5177600	1846657	8.6	6.4034
RPL32	D3YTB1	60S ribosomal protein L32	15.616	2	2	43773000	13918667	11443594	17.3	12.525
HNRNPAB	D6R9P3	Heterogeneous nuclear ribonucleoprotein A/B	30.302	9	9	9.11E+08	3.02E+08	1.31E+08	32.5	90.208
RPL9	D6RAN4	60S ribosomal protein L9	20.775	4	4	2.29E+08	75497000	19093200	28.2	43.128
RPL14	E7EPB3	60S ribosomal protein L14	14.558	2	2	1.9E+08	60812000	18491984	19.4	18.211
EIF4A2	E7EQG2	Eukaryotic initiation factor 4A-II	41.29	4	1	4314100	1438033	2033686	14.4	6.2353
RPL15	E7EQV9	Ribosomal protein L15	20.51	3	3	2.42E+08	80500667	24692297	20.1	33.956
CSTF2	E7EWR4	Cleavage stimulation factor subunit 2	62.942	13	8	4.36E+08	1.45E+08	35460235	32.8	130.5
EIF4B	E7EX17	Eukaryotic translation initiation factor 4B	69.697	4	4	2.16E+08	71847000	44209096	8.3	24.049
RPL8	P62917	60S ribosomal protein L8	22.389	4	4	2.1E+08	68201333	24911114	21	28.487
SF3B2	Q13435	Splicing factor 3B subunit 2	98.169	14	14	5.73E+08	1.9E+08	26860195	27.3	180.97
SEC23A	F5H365	Protein transport protein Sec23A	82.968	17	15	1.07E+09	3.57E+08	87134604	32.7	323.31

CPSF7	Q8N684	Cleavage and polyadenylation specificity factor subunit 7	41.265	9	9	3.3E+08	1.1E+08	35219778	31.8	87.814
CNOT2	F8VV52	CCR4-NOT transcription complex subunit 2	58.62	6	6	1.31E+08	43610000	19372095	19.6	41.41
PCBP2	Q15366	Poly(rC)-binding protein 2	31.6	4	2	1.22E+08	40618333	14275847	23.3	29.978
UBAP2L	F8W726	Ubiquitin-associated protein 2-like	113.63	12	12	2.96E+08	96647667	54538757	20.7	125.17
TIA1	F8W8I6	Nucleolysin TIA-1 isoform p40	42.835	3	1	15533000	5177600	1846657	8.6	6.4034
CPSF6	F8WJN3	Cleavage and polyadenylation specificity factor subunit 6	52.269	6	6	2.96E+08	98746333	38828479	19.5	90.824
RPL18	G3V203	60S ribosomal protein L18	18.756	4	4	2.14E+08	71221333	37582527	28.7	56.795
CPSF3	G5E9W3	Cleavage and polyadenylation specificity factor subunit 3	73.476	3	3	64287000	21429000	7647672	9	26.735
SEC24C	G5EA31	Protein transport protein Sec24C	111.98	10	10	3.04E+08	1.01E+08	19359843	15.9	94.729
RNPS1	H3BV80	RNA-binding protein with serine-rich domain 1	24.561	3	3	60923000	20307333	5903472	13.7	18.024
HNRNPUL2-BSCL2	Q1KMD3	Heterogeneous nuclear ribonucleoprotein U-like protein 2	84.69	5	5	82806000	27601667	9662189	9.5	38.607
NUP93	Q8N1F7	Nuclear pore complex protein Nup93	99.554	17	17	5.57E+08	1.86E+08	42675678	26.5	175
RPS13	P62277	40S ribosomal protein S13	16.733	4	4	2.32E+08	77227000	33414169	25.7	30.693
SEC16A	J3KNL6	Protein transport protein Sec16A	251.89	12	12	2.9E+08	96688000	22380998	10.1	99.859
DDX5	J3KTA4	Probable ATP-dependent RNA helicase DDX5	69.086	14	10	1.01E+09	3.35E+08	93471736	20.7	71.319
SRSF1	J3KTL2	Serine/arginine-rich splicing factor 1	28.329	10	10	4.09E+08	1.36E+08	52024409	39.1	90.09
CHERP	J3QK89	Calcium homeostasis endoplasmic reticulum protein	104.93	4	4	1.17E+08	38942000	14703374	8.7	42.739
SNRPN	J3QLE5	Small nuclear ribonucleoprotein-associated protein N;Small nuclear ribonucleoprotein-associated proteins B and B	17.546	6	6	6.46E+08	2.14E+08	79038972	26.6	43.252
BUB3	J3QT28	Mitotic checkpoint protein BUB3	31.703	3	3	1.93E+08	64179667	20538942	16.9	52.297
RPS15	K7ELC2	40S ribosomal protein S15	17.723	4	4	2.55E+08	83420000	27206577	53.3	98.582
RPS5	M0R0F0	40S ribosomal protein S5	22.391	4	4	1.19E+08	39752667	11751452	22	30.181
RPL18A	M0R3D6	60S ribosomal protein L18a	16.714	6	6	1.69E+08	56262667	21238729	26.2	35.768
EIF3F	O00303	Eukaryotic translation initiation factor 3 subunit F	37.563	5	5	73646000	24548667	5946849	25.8	42.639

IGF2BP3	O00425	Insulin-like growth factor 2 mRNA-binding protein 3	63.704	4	4	79788000	26596000	5813681	10.4	30.773
DHX15	O43143	Pre-mRNA-splicing factor ATP-dependent RNA helicase DHX15	90.932	10	10	2.81E+08	93700667	20932463	18.1	86.972
HNRNPR	O43390	Heterogeneous nuclear ribonucleoprotein R	70.942	15	13	8.91E+08	2.97E+08	1.41E+08	31	160.01
NUDT21	O43809	Cleavage and polyadenylation specificity factor subunit 5	26.227	10	10	8.31E+08	2.77E+08	1.03E+08	52	110.37
SYNCRIP	O60506	Heterogeneous nuclear ribonucleoprotein Q	69.602	8	6	2.36E+08	78557000	15615699	19.9	53.446
PDCD6	O75340	Programmed cell death protein 6	21.868	5	5	1.49E+08	49737333	20191068	27.7	32.441
RPS11	M0QZC5	40S ribosomal protein S11	13.997	4	4	1.88E+08	62730333	16233892	28.8	25.07
SF3B1	O75533	Splicing factor 3B subunit 1	145.83	19	19	3.43E+08	1.14E+08	34090728	25	165.89
ACTL6A	O96019	Actin-like protein 6A	47.46	9	9	5E+08	1.67E+08	39817670	33.6	112.33
RPLP2	P05387	60S acidic ribosomal protein P2	11.665	6	6	2.74E+08	91168333	35088204	70.4	73.644
RPLP0	P05388	60S acidic ribosomal protein P0	34.273	5	5	1.56E+08	51935333	12718032	18.9	75.437
SNRNP70	P08621	U1 small nuclear ribonucleoprotein 70 kDa	51.556	6	6	3.09E+08	1.03E+08	31730854	19.7	51.948
RPS17	P08708	40S ribosomal protein S17	15.55	4	4	1.77E+08	58914333	15879016	40.7	37.126
CLTA	P09496	Clathrin light chain A	27.076	6	6	1.5E+09	4.99E+08	2.08E+08	16.9	39.124
CLTB	P09497	Clathrin light chain B	25.19	6	6	2.79E+08	93003333	69074442	20.5	36.757
U2AF1	P0DN76	Splicing factor U2AF 35 kDa subunit	27.872	3	3	1.24E+08	41168000	13816244	17.9	27.032
RPS2	P15880	40S ribosomal protein S2	31.324	8	8	8.03E+08	2.65E+08	69961282	28.3	63.573
RPA2	P15927	Replication protein A 32 kDa subunit	29.247	5	5	2.06E+08	65662000	14183695	36.3	41.083
HIST1H1C	P16403	Histone H1.2	21.364	4	2	1.36E+08	45252333	17376704	19.7	30.472
RPL7	P18124	60S ribosomal protein L7	29.225	5	5	1.94E+08	64530667	24770131	23.8	33.342
FLNA	Q5HY54	Filamin-A	276.55	39	34	1.97E+09	6.56E+08	1.73E+08	21.6	323.31
TUBG1	P23258	Tubulin gamma-1 chain	51.169	9	9	2.42E+08	80771333	23516041	35.5	125.18
RPS3	P23396	40S ribosomal protein S3	26.688	12	12	1.46E+09	4.85E+08	1.69E+08	59.3	101.53
ATP5A1	P25705	ATP synthase subunit alpha, mitochondrial	59.75	3	3	56556000	18852000	6100154	6.1	20.802
DDX6	P26196	Probable ATP-dependent RNA helicase DDX6	54.416	12	12	4.92E+08	1.64E+08	50951271	41.8	233.99

RPA1	P27694	Replication protein A 70 kDa DNA-binding subunit	68.137	9	9	4.22E+08	1.41E+08	46632752	21.6	102.62
DNAJA1	P31689	DnaJ homolog subfamily A member 1	44.868	3	3	49725000	15604667	1265000	14.1	35.866
HNRNPH3	P31942	Heterogeneous nuclear ribonucleoprotein H3	36.926	11	11	1.41E+09	4.63E+08	1.38E+08	53.5	223.95
RPA3	P35244	Replication protein A 14 kDa subunit	13.569	4	4	2.03E+08	67621667	25523794	52.9	59.904
FUS	P35637	RNA-binding protein FUS	53.425	9	7	2.31E+09	7.53E+08	2.32E+08	20.2	182.56
NUP214	P35658	Nuclear pore complex protein Nup214	213.62	21	21	6.94E+08	2.31E+08	56664093	17	264.17
NUP62	P37198	Nuclear pore glycoprotein p62	53.254	5	5	2.19E+08	72834333	11237662	14.2	66.281
RBMX	P38159	RNA-binding motif protein, X chromosome	42.331	7	7	3.05E+08	1.02E+08	26145106	22	77.039
CCT6A	P40227	T-complex protein 1 subunit zeta	58.024	5	5	67515000	22505000	3457892	14.5	36.206
RPL13A	P40429	60S ribosomal protein L13a	23.577	5	5	2.83E+08	93679333	29037301	22.2	34.495
RANGAP1	P46060	Ran GTPase-activating protein 1	63.541	6	6	2.57E+08	85617667	38372586	17.7	58.495
RPL5	P46777	60S ribosomal protein L5	34.362	4	4	1.68E+08	56142667	17058073	14.5	31.681
CCT3	P49368	T-complex protein 1 subunit gamma	60.533	4	4	53392000	17797067	10503772	10.3	30.043
RANBP2	P49792	E3 SUMO-protein ligase RanBP2	358.2	11	8	1.69E+08	56264000	14069073	4.8	64.181
KPNA2	P52292	Importin subunit alpha-1	57.861	8	8	1.45E+08	48428000	19065838	29.3	109.47
CAPZA1	P52907	F-actin-capping protein subunit alpha-1	32.922	3	2	1.58E+08	51822333	8767841	15.4	24.892
NUP98	P52948	Nuclear pore complex protein Nup98-Nup96	197.58	9	9	4.05E+08	1.35E+08	26011798	7.2	91.445
VCP	P55072	Transitional endoplasmic reticulum ATPase	89.321	3	3	49642000	16547167	5864153	4.2	20.025
SEC13	P55735	Protein SEC13 homolog	35.54	3	3	6.47E+08	2.16E+08	64740319	16.8	124.17
EIF4A1	P60842	Eukaryotic initiation factor 4A-I	46.153	4	1	1.17E+08	39045000	9262835	18.2	40.047
RPS7	P62081	40S ribosomal protein S7	22.127	6	6	3.39E+08	1.07E+08	17278929	43.8	49.69
PPP1CA	P62136	Serine/threonine-protein phosphatase PP1-alpha catalytic subunit	37.512	3	2	56168000	18722667	7681275	11.8	20.918
PPP1CB	P62140	Serine/threonine-protein phosphatase PP1-beta catalytic subunit	37.186	2	1	34054000	11351267	2787457	8.3	14.781
RPS14	P62263	40S ribosomal protein S14	16.273	6	6	2.53E+08	84441000	19982896	39.7	52.318
SNRPD1	P62314	Small nuclear ribonucleoprotein Sm D1	13.281	4	4	4.84E+08	1.61E+08	45809445	54.6	71.951

SNRPD2	P62316	Small nuclear ribonucleoprotein Sm D2	13.527	3	3	2.82E+08	93935667	26236959	30.5	27.551
SNRPD3	P62318	Small nuclear ribonucleoprotein Sm D3	13.916	2	2	5.29E+08	1.76E+08	34538761	15.1	23.461
RPS4X	P62701	40S ribosomal protein S4, X isoform	29.597	7	7	2.5E+08	83284333	35009760	25.5	47.309
RPS6	P62753	40S ribosomal protein S6	28.68	3	3	3.28E+08	1.07E+08	37105414	14.1	42.005
RPL31	P62899	60S ribosomal protein L31	14.463	4	4	3.06E+08	1.02E+08	27547573	32.8	37.042
RPL10A	P62906	60S ribosomal protein L10a	24.831	6	6	1.48E+08	49357667	26047816	28.1	41.302
GNB2L1	P63244	Guanine nucleotide-binding protein subunit beta-2-like 1	35.076	5	5	1.49E+08	49756667	25101393	17.4	34.607
RAE1	P78406	mRNA export factor	40.968	7	7	4.22E+08	1.41E+08	31545396	31	142.15
RBM3	P98179	RNA-binding protein 3	17.17	3	3	2.08E+08	69243667	16072716	38.2	46.433
PLOD1	Q02809	Procollagen-lysine,2-oxoglutarate 5-dioxygenase 1	83.549	6	6	1.11E+08	37143667	27607903	10	55.058
RPL6	Q02878	60S ribosomal protein L6	32.728	9	9	6.13E+08	2.04E+08	92519353	39.2	108.88
KHDRBS1	Q07666	KH domain-containing, RNA-binding, signal transduction-associated protein 1	48.227	5	5	2.8E+08	93345667	22069688	15.3	43.529
RBBP4	Q09028	Histone-binding protein RBBP4	47.655	2	2	21311000	7103767	4739072	5.2	16.236
CPSF1	Q10570	Cleavage and polyadenylation specificity factor subunit 1	160.88	9	9	1.2E+08	39884333	12501282	7.7	68.629
ILF3	Q12906	Interleukin enhancer-binding factor 3	95.337	16	16	4.24E+08	1.41E+08	44328491	26.8	122.7
CSTF3	Q12996	Cleavage stimulation factor subunit 3	82.921	5	5	90749000	30249667	12576693	10.2	56.041
ACACA	Q13085	Acetyl-CoA carboxylase 1;Biotin carboxylase	265.55	6	6	67321000	22440333	5999187	3.8	44.522
HNRNPA0	Q13151	Heterogeneous nuclear ribonucleoprotein A0	30.84	6	6	1.07E+09	3.56E+08	1.03E+08	23.6	62.111
SRSF6	Q13247	Serine/arginine-rich splicing factor 6	39.586	4	4	2.35E+08	78221333	30146132	12.5	38.488
G3BP1	Q13283	Ras GTPase-activating protein-binding protein 1	52.164	15	13	1.38E+09	4.56E+08	1.23E+08	44	252.4
EIF3I	Q13347	Eukaryotic translation initiation factor 3 subunit I	36.501	6	6	1.26E+08	41868000	14835965	25.2	56.992
EIF3A	Q14152	Eukaryotic translation initiation factor 3 subunit A	166.57	6	6	1.12E+08	37221667	15558870	5.3	48.933
CTTN	Q14247	Src substrate cortactin	61.585	5	5	71658000	23886067	22716666	14.4	42.499
FLNA	Q5HY54	Filamin-A	276.55	39	34	1.97E+09	6.56E+08	1.73E+08	21.6	323.31
CAPRIN1	Q14444	Caprin-1	78.365	12	12	1.22E+09	4.06E+08	1.53E+08	28.1	155.8

PCBP1	Q15365	Poly(rC)-binding protein 1	37.497	6	4	3.2E+08	1.07E+08	55073009	29.8	102.48
SEC23B	Q15437	Protein transport protein Sec23B	86.478	8	6	90185000	29664333	16666381	14	38.9
SF1	Q15637	Splicing factor 1	68.329	13	13	1.01E+09	3.38E+08	1E+08	26.1	197.33
ELAVL1	Q15717	ELAV-like protein 1	36.091	5	5	1.4E+08	46626000	12409680	21.8	38.561
FAM98B	Q52LJ0	Protein FAM98B	37.19	8	6	3.53E+08	1.17E+08	38819306	30	133.73
ZNF326	Q5BKZ1	DBIRD complex subunit ZNF326	65.653	7	7	94068000	31356333	13876664	17.7	44.599
NUP188	Q5SRE5	Nucleoporin NUP188 homolog	196.04	8	8	1.24E+08	41238667	16224625	6.5	61.748
PRPF8	Q6P2Q9	Pre-mRNA-processing-splicing factor 8	273.6	3	3	22111000	7370100	5189206	1.7	17.379
FIP1L1	Q6UN15	Pre-mRNA 3-end-processing factor FIP1	66.526	5	5	2.24E+08	74564667	25095102	14.1	50.219
ALYREF	Q86V81	THO complex subunit 4	26.888	4	4	53655000	17885333	4996337	32.3	38.609
CARM1	Q86X55	Histone-arginine methyltransferase CARM1	65.853	11	11	5.74E+08	1.91E+08	71260991	28	173.21
CCAR1	Q8IX12	Cell division cycle and apoptosis regulator protein 1	132.82	11	11	3.29E+08	1.1E+08	27213686	11.7	124.33
SERBP1	Q8NC51	Plasminogen activator inhibitor 1 RNA-binding protein	44.965	4	4	1.25E+08	41687333	32462891	13	31.658
FAM98A	Q8NCA5	Protein FAM98A	55.4	7	5	2.32E+08	77214333	30306653	18.7	44.008
SMARCC2	Q8TAQ2	SWI/SNF complex subunit SMARCC2	132.88	6	3	75680000	25227000	4859368	6.1	47.005
ATXN2L	Q8WWM7	Ataxin-2-like protein	113.37	8	8	2.86E+08	95454000	34211004	12.6	61.185
PALLD	Q8WX93	Palladin	150.56	7	7	1.15E+08	38490667	18521870	8.9	57.144
PSPC1	Q8WXF1	Paraspeckle component 1	58.743	10	10	4.94E+08	1.65E+08	40973611	27.2	124.27
TFG	Q92734	Protein TFG	43.447	12	12	1.3E+09	4.32E+08	1.08E+08	37.5	323.31
SMARCC1	Q92922	SWI/SNF complex subunit SMARCC1	122.87	10	7	2.22E+08	73160000	26372104	12.4	82.52
TNPO1	Q92973	Transportin-1	102.35	11	11	6.03E+08	2.01E+08	71163542	15.7	91.77
SMARCE1	Q969G3	SWI/SNF-related matrix-associated actin-dependent regulator of chromatin subfamily E member 1	46.649	5	5	1.51E+08	50186667	21458437	20.9	53.643
FUBP1	Q96AE4	Far upstream element-binding protein 1	24.043	11	1	1.35E+08	45064000	11503995	65.5	7.7765
FUBP3	Q96I24	Far upstream element-binding protein 3	61.64	10	9	3.38E+08	1.13E+08	26313774	25.7	73.998
FAM103A1	Q9BTL3	RNMT-activating mini protein	14.381	3	3	75592000	25197333	5576240	39	20.184
RBM4	Q9BWF3	RNA-binding protein 4	40.313	3	3	50097000	16698900	7282507	14.8	55.113

SF3B5	Q9BWJ5	Splicing factor 3B subunit 5	10.135	2	2	1.02E+08	33900333	8570562	25.6	32.855
FAM120A	Q9NZB2	Constitutive coactivator of PPAR-gamma-like protein 1	121.89	3	3	64779000	21593333	4063169	3.8	19.07
G3BP2	Q9UN86	Ras GTPase-activating protein-binding protein 2	54.12	9	7	2.41E+08	76927000	22610543	24.9	70.576
C14orf166	Q9Y224	UPF0568 protein C14orf166	28.068	10	10	1.72E+09	5.74E+08	1.33E+08	45.1	160.75
THRAP3	Q9Y2W1	Thyroid hormone receptor-associated protein 3	108.66	3	3	49141000	16380333	2768672	3.6	18.011
LUC7L2	Q9Y383	Putative RNA-binding protein Luc7-like 2	46.513	4	4	56435000	18811333	3984318	12	27.023
RTCB	Q9Y3I0	tRNA-splicing ligase RtcB homolog	55.21	21	21	3.41E+09	1.13E+09	3.09E+08	47.1	282.27
YTHDF2	Q9Y5A9	YTH domain-containing family protein 2	62.333	5	5	1.43E+08	46991667	11593112	15	35.505
SEC23IP	Q9Y6Y8	SEC23-interacting protein	111.08	9	9	1.89E+08	62938667	23867668	11.6	74.654
CSTF2T	Q9H0L4	Cleavage stimulation factor subunit 2	62.942	13	8	4.36E+08	1.45E+08	35460235	32.8	130.5

Supplementary Table 3: List of protein hits identified in IP-MS experiments of Kpnβ1 from WHCO5 cell extracts

Gene names	Majority protein IDs	Protein names	Mol. weight [kDa]	Total Peptides	Unique peptides	Total Intensity	Mean intensity	Standard deviation of intensity	Sequence coverage [%]	Score
KPNB1	Q14974	Importin subunit beta-1	97.169	6	6	1.49E+08	49755333	15745493	8.7	44.051
TAF15	Q92804	TATA-binding protein-associated factor 2N	48.839	4	2	2.59E+08	86219333	8736860	14.5	27.718
HNRNPDL	A0A087WUK2	Heterogeneous nuclear ribonucleoprotein D-like	40.04	8	6	4.05E+08	1.35E+08	27693669	21.8	57.498
RPS24	E7ETK0	40S ribosomal protein S24	15.197	5	5	1.81E+08	57340000	12069685	35.9	31.96
FLNA	Q5HY54	Filamin-A	245.85	6	5	73648000	24549333	5294367	3.9	43.272
SRSF3	A0A087X2D0	Serine/arginine-rich splicing factor 3	10.32	4	3	3.47E+08	1.16E+08	35869349	49.5	45.624
RBM10	A0A0A0MR66	RNA-binding protein 10	110.36	4	4	43625000	14541667	2405486	5.5	25.857
HNRNPUL1	A0A0A0MRA5	Heterogeneous nuclear ribonucleoprotein U-like protein 1	85.939	9	9	2.93E+08	97243333	34082194	21.9	73.085
NUP214	P35658	Nuclear pore complex protein Nup214	152.57	9	9	2.07E+08	68903667	19184260	10.7	101.43
SMARCA4	Q9HBD4	Transcription activator BRG1	188.15	13	13	2.23E+08	74421333	4663380	10.2	120.95
TCEB2	Q15370	Transcription elongation factor B polypeptide 2	7.7561	2	2	4687900	1335283	566529.8	12.1	6.449
FAM120B	A0A0D9SEJ5	Constitutive coactivator of peroxisome proliferator-activated receptor gamma	105.19	3	3	27905000	9301733	2933527	6.5	19.042
DDX3X	A0A0D9SFB3	ATP-dependent RNA helicase DDX3X	70.839	19	18	6.88E+08	2.29E+08	64941740	37.2	191.82
FASN	A0A0U1RQF0	Fatty acid synthase	273.2	8	8	1.09E+08	36361333	2415639	5.3	58.336
PTBP1	A0A0U1RRM4	Polypyrimidine tract-binding protein 1	56.51	9	9	6.22E+08	2.07E+08	65440637	28.7	107.06
DDX17	A0A1X7SBZ2	Probable ATP-dependent RNA helicase DDX17	80.253	26	21	3.24E+09	1.08E+09	1.8E+08	40.3	314.53
CNOT1	A5YKK6	CCR4-NOT transcription complex subunit 1	266.94	21	21	5.02E+08	1.67E+08	76109474	10.7	139.87
RPL23A	H7BY10	60S ribosomal protein L23a	17.692	4	4	1.35E+08	35540367	24972346	29.7	31.786
EWSR1	B0QYK0	RNA-binding protein EWS	64.929	5	5	1.23E+09	4.05E+08	85452248	11.8	123.91
XRCC6	B1AHC9	X-ray repair cross-complementing protein 6	64.283	5	5	60498000	19242000	4389487	12.5	30.89
PABPC4	B1ANR0	Polyadenylate-binding protein	67.97	26	18	2.68E+09	8.87E+08	1.26E+08	43.7	323.31

ILF2	B4DY09	Interleukin enhancer-binding factor 2	38.91	6	6	2.01E+08	66885000	20810240	20.7	40.925
RAN	B5MDF5	GTP-binding nuclear protein Ran	26.224	2	2	70170000	23390000	5061448	10.7	14.666
MYL6	F8W1R7	Myosin light polypeptide 6	14.436	3	3	64428000	20160000	4216215	33.8	19.724
SMARCD2	J3KMX2	SWI/SNF-related matrix-associated actin-dependent regulator of chromatin subfamily D member 2	52.238	7	7	1.84E+08	61384000	9511923	22.8	49.973
POLR2B	C9J2Y9	DNA-directed RNA polymerase	133.06	12	12	2.87E+08	95750333	9993617	14.6	123.55
HNRNPAB	D6R9P3	Heterogeneous nuclear ribonucleoprotein A/B	30.302	11	11	1.99E+09	6.64E+08	73133958	36.1	140.79
RPL9	D6RAN4	60S ribosomal protein L9	20.775	4	4	1.86E+08	61886667	6605675	24.3	33.939
SEC31A	D6REX3	Protein transport protein Sec31A	136.22	11	11	2.44E+08	81037667	36344448	11	71.841
CSTF2	E7EWR4	Cleavage stimulation factor subunit 2	62.942	12	6	4.68E+08	1.56E+08	16002769	29.1	169.99
RPL27A	E9PLL6	60S ribosomal protein L27a	12.201	2	2	1.86E+08	59352333	4558951	22.2	13.979
MTHFD1	F5H2F4	C-1-tetrahydrofolate synthase, cytoplasmic	110.61	7	7	1.41E+08	47129000	20644979	8.2	49.123
SEC23A	F5H365	Protein transport protein Sec23A	82.968	10	8	2.97E+08	98933000	35748214	15.6	147.06
CPSF7	Q8N684	Cleavage and polyadenylation specificity factor subunit 7	41.265	5	5	1.07E+08	35612667	8995529	21.7	34.698
CAD	F8VPD4	CAD protein	236.02	42	42	2.37E+09	7.88E+08	2.11E+08	30.2	323.31
RPLP0	P05388	60S acidic ribosomal protein P0	15.813	4	4	1.12E+08	37245333	23540295	32.4	30.779
RPL18	G3V203	60S ribosomal protein L18	14.529	2	2	2.21E+08	67388333	6708149	20	35.169
CNOT2	F8VV52	CCR4-NOT transcription complex subunit 2	58.62	9	9	3.43E+08	1.14E+08	15865397	35.6	85.995
PCBP2	Q15366	Poly(rC)-binding protein 2	31.6	4	3	1.93E+08	64296667	10517687	24.9	52.563
IGF2BP2	F8W930	Insulin-like growth factor 2 mRNA-binding protein 2	66.785	6	5	2.52E+08	83946667	19354159	12.9	96.785
CPSF6	F8WJN3	Cleavage and polyadenylation specificity factor subunit 6	52.269	4	4	1.22E+08	40564000	4455695	11.9	29.908
SEC24C	G5EA31	Protein transport protein Sec24C	111.98	15	15	4.96E+08	1.65E+08	58721964	23.1	132.31
HNRNPUL2-BSCL2	Q1KMD3	Heterogeneous nuclear ribonucleoprotein U-like protein 2	84.69	3	3	89518000	29839333	11032180	5.1	22.255
SEC16A	J3KNL6	Protein transport protein Sec16A	251.89	13	13	2.75E+08	91548000	11773947	10	110.88
DDX5	J3KTA4	Probable ATP-dependent RNA helicase DDX5	69.086	14	9	7.41E+08	2.47E+08	55019249	23.8	67.013

SRSF1	J3KTL2	Serine/arginine-rich splicing factor 1	28.329	6	5	2.17E+08	72272667	24249678	24.5	44.474
CHERP	J3QK89	Calcium homeostasis endoplasmic reticulum protein	104.93	7	7	2.01E+08	67047667	26010489	12.9	64.541
SNRPN	J3QLE5	Small nuclear ribonucleoprotein-associated protein N	17.546	6	6	7.28E+08	2.38E+08	65597716	26.6	54.513
RPS11	M0QZC5	40S ribosomal protein S11	13.997	5	5	1.01E+08	32616333	24795999	43.2	31.63
RPL18A	M0R3D6	60S ribosomal protein L18a	16.714	5	5	1.16E+08	38518667	9432745	30.5	30.82
SNRPA	P09012	U1 small nuclear ribonucleoprotein A	28.388	4	4	1.79E+08	59546000	17555118	22.3	45.992
SHKBP1	M0R2P6	SH3KBP1-binding protein 1	73.707	6	6	1.05E+08	35073000	16591094	19.2	47.705
ARID1A	O14497	AT-rich interactive domain-containing protein 1A	242.04	17	17	3.99E+08	1.33E+08	27257371	12	125.65
XPO1	O14980	Exportin-1	123.38	4	4	62937000	20979000	2019307	5.4	26.326
U2SURP	O15042	U2 snRNP-associated SURP motif-containing protein	118.29	7	7	1.66E+08	55271000	18356144	11.5	46.299
RNMT	O43148	mRNA cap guanine-N7 methyltransferase	54.843	7	7	1.6E+08	53215333	20265862	19.7	65.52
HNRNPR	O43390	Heterogeneous nuclear ribonucleoprotein R	70.942	10	8	2.82E+08	93001000	34242982	21.3	66.18
NUDT21	O43809	Cleavage and polyadenylation specificity factor subunit 5	26.227	8	8	3.43E+08	1.14E+08	35532039	45.4	64.926
SYNCRIP	O60506	Heterogeneous nuclear ribonucleoprotein Q	69.602	8	6	1.62E+08	53925000	28739844	23	58.765
FLNB	O75369	Filamin-B	278.16	9	8	1.29E+08	42857333	3792172	5.3	77.044
SF3B1	O75533	Splicing factor 3B subunit 1	145.83	14	14	2.77E+08	92304333	21994148	17.7	99.575
ACTL6A	O96019	Actin-like protein 6A	47.46	7	7	4.07E+08	1.36E+08	45248906	28.7	91.505
SNRNP70	P08621	U1 small nuclear ribonucleoprotein 70 kDa	51.556	7	7	4.13E+08	1.38E+08	31801824	27.9	65.747
RPS17	P08708	40S ribosomal protein S17	15.55	5	5	1.81E+08	58107667	12940678	45.9	47.902
CLTA	P09496	Clathrin light chain A	27.076	8	8	1.51E+09	5.02E+08	13126331	20.6	85.497
CLTB	P09497	Clathrin light chain B	25.19	5	5	5.26E+08	1.75E+08	27702060	16.6	66.736
PABPC1	P11940	Polyadenylate-binding protein 1	70.67	24	16	1.72E+09	5.73E+08	98458990	48.1	158.33
DSP	P15924	Desmoplakin	331.77	69	69	2.55E+09	8.5E+08	1.58E+08	27.1	323.31
RPL7	P18124	60S ribosomal protein L7	29.225	5	5	1.29E+08	42146333	5372423	22.6	43.467
TUBG1	P23258	Tubulin gamma-1 chain	51.169	5	5	1.53E+08	50918000	21825607	15.1	45.454

POLR2A	P24928	DNA-directed RNA polymerase II subunit RPB1	217.17	10	10	1.43E+08	47649000	4464412	9	90.459
DDX6	P26196	Probable ATP-dependent RNA helicase DDX6	54.416	10	10	2.63E+08	87720000	33370790	35.4	136.61
RPL10	P27635	60S ribosomal protein L10	24.604	6	6	2.01E+08	66763000	11197930	22	43.073
RPA1	P27694	Replication protein A 70 kDa DNA-binding subunit	68.137	2	2	49312000	16437333	1215033	3.7	12.029
RPL12	P30050	60S ribosomal protein L12	17.818	3	3	1.39E+08	46458667	15331666	24.2	26.749
HNRNPH3	P31942	Heterogeneous nuclear ribonucleoprotein H3	36.926	12	12	1.4E+09	4.6E+08	1.17E+08	50	323.31
FUS	P35637	RNA-binding protein FUS	53.425	12	10	3.12E+09	1.03E+09	1.75E+08	22.2	245.61
RBMX	P38159	RNA-binding motif protein, X chromosome	42.331	8	8	3.95E+08	1.32E+08	7430623	21.7	77.799
RPL13A	P40429	60S ribosomal protein L13a	23.577	4	4	1.07E+08	34979333	5106804	16.7	27.938
RPL21	P46778	60S ribosomal protein L21	18.565	5	5	2.43E+08	78049667	15975297	36.9	40.885
RANBP2	P49792	E3 SUMO-protein ligase RanBP2	358.2	7	7	1.36E+08	45315000	20078133	3.7	52.425
HNRNPF	P52597	Heterogeneous nuclear ribonucleoprotein F	45.671	11	9	4.49E+08	1.5E+08	14592356	43.4	116.3
NUP98	P52948	Nuclear pore complex protein Nup98-Nup96	197.58	8	8	3.14E+08	1.03E+08	51614095	6.7	63.095
SEC13	P55735	Protein SEC13 homolog	35.54	3	3	4.19E+08	1.4E+08	23019233	15.5	90.62
HNRNPK	P61978	Heterogeneous nuclear ribonucleoprotein K	50.976	21	5	2.32E+09	7.64E+08	1.55E+08	47.3	302.88
RPS13	P62277	40S ribosomal protein S13	17.222	6	6	1.72E+08	57210000	13103333	30.5	38.104
SNRPE	P62304	Small nuclear ribonucleoprotein E	10.803	4	4	2.42E+08	80596000	17971031	42.4	46.737
SNRPF	P62306	Small nuclear ribonucleoprotein F	9.7251	3	3	2.91E+08	96960333	45054584	39.5	40.348
SNRPD2	P62316	Small nuclear ribonucleoprotein Sm D2	13.527	7	7	2.46E+08	81945667	58452206	48.3	64.677
SNRPD3	P62318	Small nuclear ribonucleoprotein Sm D3	13.916	3	3	7.17E+08	2.32E+08	69487505	20.6	32.378
RPS4X	P62701	40S ribosomal protein S4, X isoform	29.597	9	9	2.31E+08	77091667	37290791	37.3	64.376
RPS6	P62753	40S ribosomal protein S6	28.68	5	5	3.88E+08	1.29E+08	9381394	20.5	64.9
RPS25	P62851	40S ribosomal protein S25	13.742	2	2	4.37E+08	1.38E+08	9550656	15.2	12.706
RPL31	P62899	60S ribosomal protein L31	14.463	4	4	2.46E+08	81892333	5930311	32.8	41.901
RPL38	P63173	60S ribosomal protein L38	8.2178	3	3	2.02E+08	59982667	6904835	50	19.984

TUBA4A	P68366	Tubulin alpha-4A chain	49.924	19	2	2.14E+08	71216000	22563685	40.2	19.482
RAE1	P78406	mRNA export factor	40.968	8	8	4.67E+08	1.53E+08	33913353	35.3	148.07
RBM3	P98179	RNA-binding protein 3	17.17	3	3	1.54E+08	51427667	15459194	38.2	25.389
CSTF1	Q05048	Cleavage stimulation factor subunit 1	48.357	8	8	3.39E+08	1.13E+08	27087461	28.1	108.35
CPSF1	Q10570	Cleavage and polyadenylation specificity factor subunit 1	160.88	9	9	1.58E+08	52778000	18101908	6.2	80.442
ILF3	Q12906	Interleukin enhancer-binding factor 3	95.337	6	6	2.64E+08	88020000	17305138	11.1	108.19
CSTF3	Q12996	Cleavage stimulation factor subunit 3	82.921	6	6	1.46E+08	48573000	25064658	11.6	68.996
HNRNPA0	Q13151	Heterogeneous nuclear ribonucleoprotein A0	30.84	8	8	6.56E+08	2.19E+08	88066197	24.9	60.543
TRIM28	Q13263	Transcription intermediary factor 1-beta	88.549	5	5	65402000	21800667	4139870	10.4	50.462
SF3B2	Q13435	Splicing factor 3B subunit 2	100.23	18	18	5.74E+08	1.91E+08	45351254	28.9	159.02
HNRNPD	Q14103	Heterogeneous nuclear ribonucleoprotein D0	38.434	11	9	8.08E+08	2.69E+08	1.12E+08	25.9	87.49
TRIM29	Q14134	Tripartite motif-containing protein 29	65.834	14	14	4.56E+08	1.52E+08	25763500	28.6	144.67
PLEC	Q15149	Plectin	531.78	9	9	1.07E+08	35577667	16870892	3.2	70.022
NONO	Q15233	Non-POU domain-containing octamer-binding protein	54.231	26	24	5.91E+09	1.97E+09	2.64E+08	58.2	323.31
PCBP1	Q15365	Poly(rC)-binding protein 1	37.497	5	4	3.67E+08	1.19E+08	37689704	27.5	55.133
SF3B3	Q15393	Splicing factor 3B subunit 3	135.58	10	10	5.04E+08	1.68E+08	37650512	12.9	98.475
SF3B4	Q15427	Splicing factor 3B subunit 4	44.385	2	2	82158000	27386000	12062893	8.7	14.784
SF1	Q15637	Splicing factor 1	68.329	11	11	6.71E+08	2.24E+08	52421006	23.2	182.69
ELAVL1	Q15717	ELAV-like protein 1	36.091	6	6	2.76E+08	91941000	17285888	24.5	49.962
FAM98B	Q52LJ0	Protein FAM98B	37.19	6	5	2.73E+08	91082000	18811115	24.2	136.72
ZNF326	Q5BKZ1	DBIRD complex subunit ZNF326	65.653	10	10	2.33E+08	77641000	18488656	24.9	77.29
RALY	Q5QPM2	RNA-binding protein Raly	9.714	2	2	78386000	26128667	4424151	25.8	13.837
NUP188	Q5SRE5	Nucleoporin NUP188 homolog	196.04	6	6	43357000	14452267	10950017	4.9	39.154
EDC4	Q6P2E9	Enhancer of mRNA-decapping protein 4	151.66	7	7	2.16E+08	72113333	15988183	7.9	76.347
FIP1L1	Q6UN15	Pre-mRNA 3-end-processing factor FIP1	66.526	7	7	2.47E+08	82365000	40627910	19.4	83.183
PABPN1	Q86U42	Polyadenylate-binding protein 2	32.749	2	2	52242000	17414333	1827007	8.8	38.259

CARM1	Q86X55	Histone-arginine methyltransferase CARM1	65.853	10	10	5.06E+08	1.68E+08	56698224	25.7	95.542
CCAR1	Q8IX12	Cell division cycle and apoptosis regulator protein 1	132.82	8	8	91222000	30407667	6633852	8.5	53.467
CCAR2	Q8N163	Cell cycle and apoptosis regulator protein 2	102.9	3	3	47183000	15727667	1040003	4.3	21.923
NUP93	Q8N1F7	Nuclear pore complex protein Nup93	93.487	8	8	1.32E+08	43939000	18655059	15.9	94.621
SMARCC2	Q8TAQ2	SWI/SNF complex subunit SMARCC2	132.88	11	8	3.34E+08	1.11E+08	41665869	14.3	166.94
ATXN2L	Q8WWM7	Ataxin-2-like protein	113.37	3	3	36303000	12100833	6876574	4.6	19.755
PSPC1	Q8WXF1	Paraspeckle component 1	58.743	17	16	1.25E+09	4.17E+08	77815136	41.9	197.28
DDX1	Q92499	ATP-dependent RNA helicase DDX1	82.431	31	31	2.28E+09	7.61E+08	2.36E+08	44.2	323.31
TFG	Q92734	Protein TFG	43.447	8	8	7.18E+08	2.39E+08	1.15E+08	32.8	241.88
SYMPK	Q92797	Symplekin	141.15	6	6	87828000	29276000	11691719	8.6	55.914
KHSRP	Q92945	Far upstream element-binding protein 2	73.114	32	29	6.62E+09	2.21E+09	1.41E+08	58.8	323.31
SMARCE1	Q969G3	SWI/SNF-related matrix-associated actin-dependent regulator of chromatin subfamily E member 1	46.649	4	4	2.06E+08	68525667	11901401	21.4	56.141
FUBP1	Q96AE4	Far upstream element-binding protein 1	24.043	10	1	1.31E+08	43666000	3778810	65.1	5.7808
FUBP3	Q96I24	Far upstream element-binding protein 3	61.64	11	9	4.37E+08	1.46E+08	16337069	25.2	65.805
RBM14	Q96PK6	RNA-binding protein 14	69.491	7	7	65612000	21870667	5124070	14.3	51.466
MED15	Q96RN5	Mediator of RNA polymerase II transcription subunit 15	86.753	7	7	94276000	31425000	8342892	9	50.721
FAM103A1	Q9BTL3	RNMT-activating mini protein	14.381	5	5	2.61E+08	87014667	397646.7	64.4	55.625
TUBB6	Q9BUF5	Tubulin beta-6 chain	49.857	19	8	1.12E+09	3.69E+08	1.02E+08	50.9	220.49
DPY30	Q9C005	Protein dpy-30 homolog	11.25	4	4	85204000	28401400	14517236	76.8	44.066
TNKS1BP1	Q9C0C2	182 kDa tankyrase-1-binding protein	181.79	13	13	1.91E+08	63540333	24017544	13.1	96.391
WDR33	Q9C0J8	pre-mRNA 3 end processing protein WDR33	145.89	9	9	1.89E+08	63062333	10946137	11.5	81.3
TP63	Q9H3D4	Tumor protein 63	76.785	9	9	2.92E+08	97253333	16553061	16.8	74.892
FAM120A	Q9NZB2	Constitutive coactivator of PPAR-gamma-like protein 1	121.89	8	8	1.42E+08	47412000	11212547	12.1	56.774
C14orf166	Q9Y224	UPF0568 protein C14orf166	28.068	10	10	1.47E+09	4.89E+08	1.35E+08	41.8	178.66
RUVBL2	Q9Y230	RuvB-like 2	51.156	5	5	1.01E+08	31381000	6670129	13.8	44.854

RTCB	Q9Y3I0	tRNA-splicing ligase RtcB homolog	55.21	23	23	2.98E+09	9.93E+08	2.3E+08	51.9	306.27
SEC23IP	Q9Y6Y8	SEC23-interacting protein	111.08	3	3	87829000	29276333	4232745	5.2	23.38

Supplementary Table 4: List of protein hits identified in IP-MS experiments of Kpnβ1 from KYSE30 cell extracts

Gene names	Majority protein IDs	Protein names	Mol. weight [kDa]	Total Peptides	Unique peptides	Total Intensity	Mean intensity	Standard deviation of intensity	Sequence coverage [%]	Score
KPNB1	Q14974	Importin subunit beta-1	97.169	10	10	5.69E+08	1.9E+08	20282382	13.1	108.95
RPS9	A0A024R4M0	40S ribosomal protein S9	22.591	11	11	1.07E+09	3.56E+08	35955104	41.8	72.175
YTHDF3	A0A024R7W5	YTH domain-containing family protein 3	58.311	3	3	1.63E+08	54311000	11804278	7.7	28.658
RPL18	G3V203	60S ribosomal protein L18	15.639	4	4	7.12E+08	2.37E+08	36782400	26.3	54.743
HNRNPDL	A0A087WUK2	Heterogeneous nuclear ribonucleoprotein D-like	6.7215	1	1	30649000	10216333	8138877	31.6	14.387
RPL10	P27635	60S ribosomal protein L10	18.565	7	7	6.8E+08	2.25E+08	28240657	33.1	66.494
TARDBP	Q13148	TAR DNA-binding protein 43	26.743	5	5	4.68E+08	1.55E+08	26123775	30.9	97.564
SRSF3	A0A087X2D0	Serine/arginine-rich splicing factor 3	10.32	3	3	7.63E+08	2.54E+08	80613707	49.5	102.73
HNRNPUL1	A0A0A0MRA5	Heterogeneous nuclear ribonucleoprotein U-like protein 1	85.939	8	8	3.36E+08	1.12E+08	10074208	18.7	63.254
SNRPC	A0A0A0MRR7	U1 small nuclear ribonucleoprotein C	19.687	2	2	3.62E+08	1.21E+08	14351932	11.7	48.123
NUP214	P35658	Nuclear pore complex protein Nup214	152.57	3	3	71902000	23967333	3092652	3.2	20.549
IARS	J3KR24	Isoleucine--tRNA ligase, cytoplasmic	131.76	6	6	2.57E+08	83644333	41125253	7	37.956
CSTF1	Q05048	Cleavage stimulation factor subunit 1	38.448	2	2	1.34E+08	44604000	3862961	7	42.981
SRSF7	A0A0B4J1Z1	Serine/arginine-rich splicing factor 7	15.763	3	3	2.59E+08	86348000	13140680	37.2	30.046
DDX3X	A0A0D9SFB3	ATP-dependent RNA helicase DDX3X	70.839	7	7	2.09E+08	69631667	21329966	13.6	55.715
TJP2	A0A1B0GTW1	Tight junction protein ZO-2	140.73	6	6	1.56E+08	52101333	34851869	7.9	51.977
DDX17	A0A1X7SBZ2	Probable ATP-dependent RNA helicase DDX17	80.253	17	17	1.64E+09	5.46E+08	85568356	33.5	169.65
CNOT1	A5YKK6	CCR4-NOT transcription complex subunit 1	266.94	9	9	2.04E+08	68087667	13226223	4.1	76.261
EIF3L	B0QY89	Eukaryotic translation initiation factor 3 subunit L	70.901	14	14	5.66E+08	1.89E+08	47961166	23.1	237.34
EWSR1	B0QYK0	RNA-binding protein EWS	64.929	3	3	6.66E+08	2.22E+08	41247090	8.6	57.044
CAPZB	B1AK87	F-actin-capping protein subunit beta	29.295	5	5	2.62E+08	87210000	22212582	26.9	40.551
RAN	B5MDF5	GTP-binding nuclear protein Ran	26.224	3	3	2.33E+08	77517000	10023148	18.5	24.568
MYL6	F8W1R7	Myosin light polypeptide 6	16.29	4	4	3.79E+08	1.26E+08	24657262	35.9	38.631

RPL24	C9JXB8	60S ribosomal protein L24	14.369	3	3	8.34E+08	2.73E+08	64320914	24.8	20.254
FUBP1	C9JSZ1	Far upstream element-binding protein 1	24.043	1	1	1.61E+08	53624333	5486329	65.1	8.3273
CTNND1	C9JZR2	Catenin delta-1	104.85	5	5	86804000	28935000	4320378	9.2	57.57
RPL32	D3YTB1	60S ribosomal protein L32	15.616	5	5	2.71E+08	90294667	46617780	26.3	33.517
HNRNPAB	D6R9P3	Heterogeneous nuclear ribonucleoprotein A/B	30.302	8	8	2.13E+09	7.08E+08	45880877	31.8	87.955
EIF4A2	E7EQG2	Eukaryotic initiation factor 4A-II	41.29	6	1	26250000	8750000	12374369	20.7	6.2259
RPL15	E7EQV9	Ribosomal protein L15;60S ribosomal protein L15	20.51	5	5	6.52E+08	2.16E+08	13502475	32.2	39.391
EIF4B	E7EX17	Eukaryotic translation initiation factor 4B	69.697	12	12	1.24E+09	4.15E+08	54460744	21.3	237.95
YWHAZ	E7EX29	14-3-3 protein zeta/delta	28.036	3	1	1.62E+08	54015667	9533988	13	58.199
ALYREF	Q86V81	THO complex subunit 4	27.557	3	3	75806000	24970333	9875155	26.5	41.373
SRP9	P49458	Signal recognition particle 9 kDa protein	7.6677	2	2	2.99E+08	99553333	7828582	28.8	19.154
EEF1D	E9PMW7	Elongation factor 1-delta	28.821	6	6	5.07E+08	1.69E+08	27430507	32.2	89.024
UACA	F5H2B9	Uveal autoantigen with coiled-coil domains and ankyrin repeats	150.53	4	4	98600000	32866667	13433000	3.3	27.86
MTHFD1	F5H2F4	C-1-tetrahydrofolate synthase, cytoplasmic	110.61	8	8	1.3E+08	42825667	14008520	9.7	46.53
SEC23A	F5H365	Protein transport protein Sec23A	82.968	3	3	65691000	21897033	11971853	8.8	38.629
GIT2	F8W822	ARF GTPase-activating protein GIT2	46.988	3	3	28665000	9554767	4243023	15.9	21.397
UBAP2L	F8W726	Ubiquitin-associated protein 2-like	113.63	5	5	2.28E+08	75979333	10290365	7.2	45.603
IGF2BP2	F8W930	Insulin-like growth factor 2 mRNA-binding protein 2	66.785	9	9	9.54E+08	3.17E+08	5932695	23	182.95
CPSF6	F8WJN3	Cleavage and polyadenylation specificity factor subunit 6	52.269	4	4	1.71E+08	57121000	2414403	13.2	43.329
EIF3M	H0YCQ8	Eukaryotic translation initiation factor 3 subunit M	25.092	4	4	82589000	25572667	2318948	34.1	43.305
SSSCA1	H0YEB6	Sjoegren syndrome/scleroderma autoantigen 1	20.916	2	2	1.63E+08	54184667	27787252	11.9	26.98
RNPS1	H3BV80	RNA-binding protein with serine-rich domain 1	24.561	4	4	1.17E+08	39031333	15710290	29.4	54.049
SRSF2	J3QL05	Serine/arginine-rich splicing factor 2	15.156	2	2	2E+08	66658000	8647010	30.8	66.546
DDX5	J3KTA4	Probable ATP-dependent RNA helicase DDX5	69.086	6	6	6.68E+08	2.23E+08	16162674	17.6	40.441

RPL19	J3QR09	Ribosomal protein L19;60S ribosomal protein L19	23.134	4	4	6.36E+08	2.11E+08	77372675	18.1	53.819
SRSF1	J3KTL2	Serine/arginine-rich splicing factor 1	28.329	9	9	9.4E+08	3.13E+08	35300988	35.2	76.078
SNRPN	J3QLE5	Small nuclear ribonucleoprotein-associated protein N	17.546	6	6	1.66E+09	5.51E+08	25583907	26.6	109.67
BUB3	J3QT28	Mitotic checkpoint protein BUB3	31.703	5	5	6.09E+08	2.03E+08	25828363	23.4	48.913
EIF3K	K7ES31	Eukaryotic translation initiation factor 3 subunit K	15.875	4	4	2.99E+08	99044667	20455766	40.9	31.296
RPS11	M0QZC5	40S ribosomal protein S11	13.997	6	6	3.23E+08	1.07E+08	21264913	36.4	38.635
RPS5	M0R0F0	40S ribosomal protein S5	22.391	4	4	1.61E+08	51205333	31915585	33.5	33.742
RPL18A	M0R3D6	60S ribosomal protein L18a	16.714	6	6	4.23E+08	1.41E+08	6203032	31.2	37.08
SUPT5H	O00267	Transcription elongation factor SPT5	121	5	5	1.98E+08	66109000	51794737	5.6	72.308
IGF2BP3	O00425	Insulin-like growth factor 2 mRNA-binding protein 3	63.704	8	8	6.18E+08	2.06E+08	12697504	19.9	127.54
EIF3D	O15371	Eukaryotic translation initiation factor 3 subunit D	63.972	7	7	3.68E+08	1.19E+08	21559014	21.4	65.791
HNRNPR	O43390	Heterogeneous nuclear ribonucleoprotein R	70.942	4	4	2.35E+08	78314000	10323489	8.5	31.019
NUDT21	O43809	Cleavage and polyadenylation specificity factor subunit 5	26.227	10	10	1.09E+09	3.63E+08	52190424	55.5	86.171
HIST1H2BN	U3KQK0	Histone H2B	18.804	3	3	5.46E+08	1.79E+08	74791605	20.5	51.74
DNAJA2	O60884	DnaJ homolog subfamily A member 2	45.745	6	6	4.86E+08	1.62E+08	10016430	22.6	61.422
CSDE1	O75534	Cold shock domain-containing protein E1	88.884	5	5	1.04E+08	31132333	2699980	10.3	36.195
EIF3G	O75821	Eukaryotic translation initiation factor 3 subunit G	35.611	8	8	6.8E+08	2.27E+08	41625430	28.1	116.4
UBXN7	O94888	UBX domain-containing protein 7	54.862	6	6	2.83E+08	94293667	23323118	16.6	44.96
BAG2	O95816	BAG family molecular chaperone regulator 2	23.772	3	3	1.73E+08	57644667	13986838	20.9	56
ACTL6A	O96019	Actin-like protein 6A	47.46	7	7	3.81E+08	1.27E+08	12395070	26.6	67.984
LMNA	P02545	Prelamin-A/C;Lamin-A/C	74.139	13	13	7.94E+08	2.65E+08	38013354	29.7	183.96
RPLP2	P05387	60S acidic ribosomal protein P2	11.665	11	11	2.85E+09	9.48E+08	85467210	82.6	222.29
EPRS	P07814	Bifunctional glutamate/proline--tRNA ligase	170.59	14	14	6.68E+08	2.2E+08	71547513	12.4	115.48
HSP90AA1	P07900	Heat shock protein HSP 90-alpha	84.659	3	3	1.03E+08	34308333	4855317	14.3	22.706

SNRPB2	P08579	U2 small nuclear ribonucleoprotein B	25.486	3	3	77281000	25760400	19089187	25.3	23.216
SNRNP70	P08621	U1 small nuclear ribonucleoprotein 70 kDa	51.556	7	7	6.8E+08	2.27E+08	70726671	21.7	93.893
SNRPA	P09012	U1 small nuclear ribonucleoprotein A	31.279	4	4	4.33E+08	1.44E+08	50899700	24.8	45.929
SNRPA1	P09661	U2 small nuclear ribonucleoprotein A	28.415	4	4	1.56E+08	51889000	17747710	21.6	44.697
HIST1H1E	P10412	Histone H1.4	21.865	1	1	3.7E+08	1.23E+08	38638944	19.2	34.293
PRPS2	P11908	Ribose-phosphate pyrophosphokinase 2	34.769	2	2	4.14E+08	1.34E+08	39136440	24.2	108.58
HIST1H1B	P16401	Histone H1.5	22.58	3	3	3.95E+08	1.32E+08	15552089	17.7	30.076
HIST1H1C	P16403	Histone H1.2;Histone H1.3	21.364	3	1	95199000	31733000	4737341	17.8	7.7382
RPL35A	P18077	60S ribosomal protein L35a	12.538	2	2	3.87E+08	1.29E+08	16034069	13.6	12.214
RPL7	P18124	60S ribosomal protein L7	29.225	11	11	9.49E+08	3.16E+08	76643539	36.7	80.696
SFPQ	P23246	Splicing factor, proline- and glutamine-rich	76.149	14	14	1.69E+09	5.65E+08	1.25E+08	27.3	165.75
TUBG1	P23258	Tubulin gamma-1 chain	51.169	5	5	2.39E+08	79133667	6631388	15.7	34.796
AHCY	P23526	Adenosylhomocysteinase	47.716	4	4	1.92E+08	63847000	24737856	9.5	26.536
DDX6	P26196	Probable ATP-dependent RNA helicase DDX6	54.416	8	8	2.18E+08	72749667	9104651	32.9	128.77
EEF1G	P26641	Elongation factor 1-gamma	50.118	3	3	1.87E+08	62200667	9298014	9.2	28.794
RPA1	P27694	Replication protein A 70 kDa DNA-binding subunit	68.137	4	4	1.95E+08	64902000	8942388	8	26.775
RPL12	P30050	60S ribosomal protein L12	17.818	3	3	1.32E+09	4.35E+08	51525283	24.2	74.942
PPP2R1A	P30153	Serine/threonine-protein phosphatase 2A 65 kDa regulatory subunit A alpha isoform	65.308	7	7	1.23E+08	41162000	21760358	18.2	49.756
DNAJA1	P31689	DnaJ homolog subfamily A member 1	44.868	7	7	4.75E+08	1.57E+08	23465659	30.2	67.426
HNRNPH3	P31942	Heterogeneous nuclear ribonucleoprotein H3	36.926	8	8	1.22E+09	3.96E+08	45754270	42.2	154.17
FUS	P35637	RNA-binding protein FUS	53.425	9	9	1.48E+09	4.75E+08	1.11E+08	20.2	139.85
RPL13A	P40429	60S ribosomal protein L13a	23.577	5	5	4.91E+08	1.63E+08	40826552	21.7	34.833
SKIV2L2	P42285	Superkiller viralicidic activity 2-like 2	117.8	11	11	2.11E+08	70397000	5540462	14.8	76.936
RPS27	Q5T4L4	40S ribosomal protein S27	7.3564	3	3	3.33E+08	1.11E+08	7625916	37.9	24.481
MAGEA4	P43358	Melanoma-associated antigen 4	34.899	5	5	3.73E+08	1.24E+08	22497965	15.1	126.07

RANGAP1	P46060	Ran GTPase-activating protein 1	63.541	9	9	3.51E+08	1.17E+08	15896965	24.2	99.19
RPL5	P46777	60S ribosomal protein L5	34.362	7	7	1.11E+09	3.71E+08	1.23E+08	23.2	63.515
RPL21	P46778	60S ribosomal protein L21	18.565	5	5	1.48E+09	4.91E+08	50388379	36.9	76.985
CCT3	P49368	T-complex protein 1 subunit gamma	60.533	5	5	3.71E+08	1.24E+08	30114104	13.8	45.83
RBM25	P49756	RNA-binding protein 25	100.18	5	5	87360000	29120000	4682030	10.1	31.872
CCT4	P50991	T-complex protein 1 subunit delta	57.924	5	5	2.25E+08	74584333	10842573	16.5	100.81
KPNA2	P52292	Importin subunit alpha-1	57.861	6	6	1.64E+08	54526000	16838432	17.8	98.266
SEC13	P55735	Protein SEC13 homolog	35.54	3	3	4.03E+08	1.33E+08	44156931	15.5	105.39
EIF3B	P55884	Eukaryotic translation initiation factor 3 subunit B	92.48	17	17	8.33E+08	2.78E+08	64789368	26.7	182.69
EIF3E	P60228	Eukaryotic translation initiation factor 3 subunit E	52.22	10	10	1.27E+09	4.23E+08	34053607	28.5	116.8
EIF4A1	P60842	Eukaryotic initiation factor 4A-I	46.153	5	5	4.41E+08	1.47E+08	15349673	36	94.725
RPS20	P60866	40S ribosomal protein S20	13.373	4	4	1.53E+09	5.1E+08	64972920	25.2	46.106
RPS8	Q5JR95	40S ribosomal protein S8	21.879	5	5	9.04E+08	3.01E+08	55226071	32.4	143.67
YWHAE	P62258	14-3-3 protein epsilon	29.174	1	1	33024000	11007667	7805003	11.8	6.8104
RPS13	P62277	40S ribosomal protein S13	17.222	6	6	5.98E+08	1.99E+08	27965065	42.4	44.919
SNRPE	P62304	Small nuclear ribonucleoprotein E	10.803	3	3	4E+08	1.32E+08	36483317	42.4	36.231
SNRPF	P62306	Small nuclear ribonucleoprotein F	9.7251	4	4	6.67E+08	2.2E+08	52277128	80.2	84.853
SNRPD1	P62314	Small nuclear ribonucleoprotein Sm D1	13.281	4	4	9.11E+08	3.02E+08	88868720	54.6	72.499
SNRPD2	P62316	Small nuclear ribonucleoprotein Sm D2	13.527	8	8	8.25E+08	2.74E+08	13870632	54.2	70.61
SNRPD3	P62318	Small nuclear ribonucleoprotein Sm D3	13.916	2	2	1.3E+09	4.31E+08	36114946	15.1	29.092
RPL7A	P62424	60S ribosomal protein L7a	29.995	9	9	1.63E+09	5.4E+08	41560616	29.7	87.518
YPEL5	P62699	Protein yippee-like 5	13.841	2	2	58742000	19581000	691550.4	19.8	12.659
RPS4X	P62701	40S ribosomal protein S4, X isoform	29.597	9	9	7.58E+08	2.53E+08	1.21E+08	40.7	70.806
RPS6	P62753	40S ribosomal protein S6	28.68	5	5	1.02E+09	3.38E+08	37071234	18.9	56.163
RBX1	P62877	E3 ubiquitin-protein ligase RBX1	12.274	2	2	49316000	16438667	1193960	17.6	29.116
RPL31	P62899	60S ribosomal protein L31	14.463	4	4	5.79E+08	1.92E+08	66605552	32.8	38.022
RPL8	P62917	60S ribosomal protein L8	28.024	6	6	6.97E+08	2.3E+08	73818062	29.6	60.113
CCT2	P78371	T-complex protein 1 subunit beta	57.488	7	7	2.03E+08	67638333	12212296	21.7	49.875

KHDRBS1	Q07666	KH domain-containing,RNA-binding,signal transduction-associated protein 1	48.227	4	4	2.63E+08	87622000	21677678	12.2	30.392
RBBP4	Q09028	Histone-binding protein RBBP4	47.655	4	4	3.86E+08	1.28E+08	35756837	28.5	51.063
HNRNPA0	Q13151	Heterogeneous nuclear ribonucleoprotein A0	30.84	5	5	7.3E+08	2.4E+08	64582428	19	70.148
SRSF6	Q13247	Serine/arginine-rich splicing factor 6	39.586	4	4	7.88E+08	2.63E+08	19804545	14.5	37.144
TRIM28	Q13263	Transcription intermediary factor 1-beta	88.549	7	7	4.96E+08	1.65E+08	25644263	12.7	94.196
G3BP1	Q13283	Ras GTPase-activating protein-binding protein 1	52.164	12	12	1.1E+09	3.55E+08	57155995	42.1	139.37
SF3B2	Q13435	Splicing factor 3B subunit 2	100.23	11	11	6.63E+08	2.21E+08	71602104	21.3	171.32
SPTAN1	Q13813	Spectrin alpha chain, non-erythrocytic 1	284.54	12	12	4.63E+08	1.54E+08	28096736	7.5	92.418
EIF3A	Q14152	Eukaryotic translation initiation factor 3 subunit A	166.57	21	21	1.5E+09	5.01E+08	38488431	18.2	212.32
CTTN	Q14247	Src substrate cortactin	61.585	17	17	1.37E+09	4.44E+08	68440659	33.8	188.86
TRIM25	Q14258	E3 ubiquitin/ISG15 ligase TRIM25	70.973	4	4	2.04E+08	68011667	18467703	7.9	32.841
FLNA	Q5HY54	Filamin-A	276.55	48	43	5.93E+09	1.97E+09	1.22E+08	27.3	323.31
CAPRIN1	Q14444	Caprin-1	20.236	3	3	2.86E+08	95186667	23625715	34.9	48.366
EFTUD2	Q15029	116 kDa U5 small nuclear ribonucleoprotein component	109.43	11	11	4.9E+08	1.62E+08	34272695	18.1	93.553
KARS	Q15046	Lysine--tRNA ligase	68.047	9	9	3.66E+08	1.22E+08	29234136	22.9	99.523
PDIA6	Q15084	Protein disulfide-isomerase A6	48.121	4	4	3.95E+08	1.32E+08	42787385	15.2	44.438
PLEC	Q15149	Plectin	531.78	5	5	54557000	18186000	6710315	1.9	31.331
SF3B4	Q15427	Splicing factor 3B subunit 4	44.385	2	2	2.8E+08	93424000	23915212	8.7	27.038
SF1	Q15637	Splicing factor 1	68.329	7	7	8.29E+08	2.76E+08	64385497	19.6	96.365
ELAVL1	Q15717	ELAV-like protein 1	36.091	4	4	2.19E+08	73121000	11605405	17.8	30.562
HIST2H2AC	Q16777	Histone H2A type 2-C	13.988	3	2	4.47E+08	1.49E+08	11901547	44.2	30.94
HNRNPUL2	Q1KMD3	Heterogeneous nuclear ribonucleoprotein U-like protein 2	85.104	6	6	1.7E+08	56652667	14595058	13	58.663
EDC4	Q6P2E9	Enhancer of mRNA-decapping protein 4	151.66	6	6	3.03E+08	1.01E+08	15227881	7.5	98.107
FIP1L1	Q6UN15	Pre-mRNA 3-end-processing factor FIP1	66.526	5	5	1.66E+08	55197333	14409903	14.1	42.993
CARM1	Q86X55	Histone-arginine methyltransferase CARM1	65.853	9	9	6.65E+08	2.21E+08	38859372	24.7	88.945

CCAR1	Q8IX12	Cell division cycle and apoptosis regulator protein 1	132.82	7	7	2.43E+08	80576667	15954695	11	80.523
ALDH16A1	Q8IZ83	Aldehyde dehydrogenase family 16 member A1	85.126	5	5	89483000	29827667	14158439	7.9	51.368
CCAR2	Q8N163	Cell cycle and apoptosis regulator protein 2	102.9	19	19	1.29E+09	4.3E+08	54702778	31.4	201.25
NUP93	Q8N1F7	Nuclear pore complex protein Nup93	93.487	6	6	65232000	21744000	4133778	12.2	55.296
CPSF7	Q8N684	Cleavage and polyadenylation specificity factor subunit 7	52.049	8	8	3.93E+08	1.31E+08	14273519	24.4	68.502
SMARCC2	Q8TAQ2	SWI/SNF complex subunit SMARCC2	132.88	2	2	1.97E+08	65467667	19995512	5.8	52.692
PPP1R13L	Q8WUF5	RelA-associated inhibitor	89.09	5	5	84870000	27983667	9634257	7.7	34.949
PSPC1	Q8WXF1	Paraspeckle component 1	58.743	10	10	9.09E+08	3.03E+08	62135225	26	107.71
TFG	Q92734	Protein TFG	43.447	8	8	9.84E+08	3.28E+08	70353721	28.5	232.47
UPF1	Q92900	Regulator of nonsense transcripts 1	124.34	5	5	1.64E+08	54775000	2803018	5.2	32.778
SMARCC1	Q92922	SWI/SNF complex subunit SMARCC1	122.87	1	1	13410000	4470000	6321535	4.3	14.804
KHSRP	Q92945	Far upstream element-binding protein 2	73.114	18	18	2.6E+09	8.64E+08	1.64E+08	40.6	260.04
FUBP1	Q96AE4	Far upstream element-binding protein 1	24.043	1	1	1.61E+08	53624333	5486329	65.1	8.3273
FUBP3	Q96I24	Far upstream element-binding protein 3	61.64	2	2	65875000	21958333	10272557	7	12.622
RBM14	Q96PK6	RNA-binding protein 14	69.491	5	5	2.81E+08	93687333	10747873	10.9	51.328
RANBP9	Q96S59	Ran-binding protein 9	77.846	4	4	1.14E+08	37917667	6220919	7.5	32.338
EIF3C	Q99613	Eukaryotic translation initiation factor 3 subunit C	105.34	15	15	1.32E+09	4.41E+08	32022580	18.4	246.42
RBM4	Q9BWF3	RNA-binding protein 4	40.313	3	3	1.22E+08	40789000	10028434	14.8	84.522
DPY30	Q9C005	Protein dpy-30 homolog	11.25	3	3	99985000	33328333	3750573	47.5	25.189
GID8	Q9NWU2	Glucose-induced degradation protein 8 homolog	26.748	3	3	1.44E+08	47916333	7339839	23.2	30.322
IGF2BP1	Q9NZI8	Insulin-like growth factor 2 mRNA-binding protein 1	63.48	2	2	92203000	30734333	8966217	6.9	20.011
UBQLN2	Q9UHD9	Ubiquilin-2	65.695	2	2	65545000	21848333	4508048	8.7	11.83
PADI3	Q9ULW8	Protein-arginine deiminase type-3	74.742	4	4	2.34E+08	74495000	5916978	10.2	26.929
UBQLN1	Q9UMX0	Ubiquilin-1	62.518	2	2	4.7E+08	1.57E+08	10189061	14.4	113.84
C14orf166	Q9Y224	UPF0568 protein C14orf166	28.068	7	7	1.53E+09	5.06E+08	34290494	37.7	114.39

RUVBL1	Q9Y265	RuvB-like 1	50.227	8	8	6.59E+08	2.2E+08	59228068	27.6	73.473
FARSA	Q9Y285	Phenylalanine--tRNA ligase alpha subunit	57.563	3	3	57344000	19114667	5305199	7.1	18.876
STRAP	Q9Y3F4	Serine-threonine kinase receptor-associated protein	38.438	6	6	1.97E+08	65120333	7318243	26	54.387

Supplementary Table 5: IP-MS summary statistics

Samples	Total spectra submitted	Total spectra identified	Peptides identified	Protein groups identified	Missed cleavages (%)		
					0	1	2
hTERT-RPE1 CTRL 1	58644	7156	752	135	73.1	23.6	3.3
hTERT-RPE1 CTRL 2			365	63			
hTERT-RPE1 CTRL 3			434	79			
hTERT-RPE1 PD 1			1481	235			
hTERT-RPE1 PD 2			1620	254			
hTERT-RPE1 PD 3			1623	249			
HeLa CTRL 1	55177	7178	293	58	75.2	22.4	2.4
HeLa CTRL 2			93	23			
HeLa CTRL 3			412	85			
HeLa PD 1			1942	309			
HeLa PD 2			1805	286			
HeLa PD 3			1994	315			
WHCO5 CTRL 1	61386	7249	301	67	67.8	26.7	5.5
WHCO5 CTRL 2			429	92			
WHCO5 CTRL 3			301	73			
WHCO5 PD 1			1771	278			
WHCO5 PD 2			1923	297			
WHCO5 PD 3			1728	270			
KYSE30 CTRL 1	66717	9220	340	70	74.3	22.1	3.6
KYSE30 CTRL 2			431	91			
KYSE30 CTRL 3			353	80			
KYSE30 PD 1			2072	346			
KYSE30 PD 2			2136	346			
KYSE30 PD 3			2166	362			

Supplementary table 6: List of 69 protein hits identified in isotype control samples of hTERT-RPE-1 cell extracts

		Isotype control			Kpnβ1 pull-down		
Protein name	Gene name	Intensity (1)	Intensity (2)	Intensity (3)	Intensity (1)	Intensity (2)	Intensity (3)
Clathrin heavy chain	CLTC	3.45E+07	1.55E+08	1.54E+07	3.72E+09	6.53E+09	5.01E+09
60S ribosomal protein L17	RPL17	8.99E+06	8.74E+06	1.96E+06	4.32E+07	5.44E+07	4.43E+07
Tumor protein D54	TPD52L2	3.43E+06	6.61E+05	1.14E+06	0.00E+00	0.00E+00	0.00E+00
Heterogeneous nuclear ribonucleoprotein M	HNRNPM	3.61E+08	9.56E+07	1.06E+08	4.52E+08	5.70E+08	5.81E+08
Calpain small subunit 1	CAPNS1	2.86E+07	3.89E+06	1.63E+07	0.00E+00	0.00E+00	0.00E+00
Heat shock 70 kDa protein 1B; 1A	HSPA1B;HSPA1A	6.31E+07	1.27E+07	2.97E+07	3.68E+07	8.98E+07	8.11E+07
Matrin-3	MATR3	3.49E+07	1.23E+07	7.83E+06	6.70E+07	1.06E+08	8.55E+07
Histone H2A type 1	HIST1H2A	8.86E+06	1.10E+07	1.71E+07	7.31E+07	1.05E+08	8.75E+07
Heterogeneous nuclear ribonucleoproteins C1/C2	HNRNPC	5.06E+06	1.60E+07	3.40E+06	1.62E+08	2.11E+08	2.68E+08
Ubiquitin-60S ribosomal protein L40; Ubiquitin-40S ribosomal protein S27a;40S ribosomal protein S27a;Polyubiquitin-B;Polyubiquitin-C	UBB;UBC;UBA52;RPS27A	1.24E+07	4.24E+06	8.31E+06	1.55E+07	2.14E+07	1.78E+07
Cdc42 effector protein 3	CDC42EP3	3.23E+07	2.49E+06	8.89E+06	0.00E+00	0.00E+00	0.00E+00
60S ribosomal protein L24	RPL24	4.32E+07	2.62E+06	1.86E+07	8.97E+07	1.01E+08	1.08E+08
Protein transport protein Sec31A	SEC31A	8.48E+06	8.33E+07	4.62E+05	8.66E+08	1.05E+09	1.05E+09
40S ribosomal protein S3a	RPS3A	5.18E+06	1.07E+07	3.01E+06	3.63E+07	0.00E+00	2.37E+07
Keratin, type I cytoskeletal 18	KRT18	3.26E+07	1.58E+06	1.58E+07	1.71E+07	2.17E+07	5.40E+07
Heterogeneous nuclear ribonucleoprotein A1; A1-like 2	HNRNPA1;HNRNPA1L2	7.98E+07	2.02E+08	8.68E+07	1.21E+09	1.70E+09	1.91E+09
Enhancer of rudimentary homolog	ERH	4.20E+07	6.19E+06	8.74E+06	0.00E+00	0.00E+00	0.00E+00
Heterogeneous nuclear ribonucleoprotein H	HNRNPH1	1.37E+08	1.97E+08	6.04E+07	1.23E+09	1.22E+09	1.44E+09
PDZ and LIM domain protein 5	PDLIM5	4.99E+06	1.01E+06	1.30E+06	1.53E+06	0.00E+00	3.71E+06
DAZ-associated protein 1	DAZAP1	2.10E+07	1.09E+08	9.72E+06	9.45E+07	1.41E+08	2.13E+08
Glyceraldehyde-3-phosphate dehydrogenase	GAPDH	2.72E+08	6.60E+07	6.49E+07	1.57E+08	2.06E+08	2.46E+08
Heat shock protein beta-1	HSPB1	8.52E+08	2.41E+08	4.39E+08	2.86E+08	3.14E+08	3.72E+08
Tubulin beta chain	TUBB	3.40E+09	8.18E+08	2.03E+09	9.52E+09	1.49E+10	1.47E+10
Vimentin	VIM	4.69E+08	9.88E+07	1.21E+08	4.96E+08	6.80E+08	8.34E+08
60 kDa heat shock protein, mitochondrial	HSPD1	1.07E+08	8.70E+06	3.23E+07	1.11E+07	3.15E+07	3.44E+07
78 kDa glucose-regulated protein	HSPA5	3.78E+08	9.01E+07	1.34E+08	2.08E+07	3.61E+07	4.34E+07
Heat shock cognate 71 kDa protein	HSPA8	9.97E+08	2.47E+08	4.13E+08	4.13E+08	4.65E+08	4.07E+08
Polyadenylate-binding protein 1	PABPC1	5.63E+07	1.81E+07	1.28E+07	5.51E+08	7.89E+08	8.35E+08
Macrophage migration inhibitory factor	MIF	7.69E+07	1.81E+07	2.58E+07	0.00E+00	0.00E+00	3.94E+06
Heterogeneous nuclear ribonucleoprotein L	HNRNPL	1.16E+07	1.13E+07	6.20E+06	3.79E+08	6.93E+08	5.03E+08
Histone H1.2;Histone H1.3	HIST1H1C;HIST1H1D	2.64E+07	4.01E+06	1.43E+06	8.39E+07	6.93E+07	1.20E+08
Heat shock 70 kDa protein 6; heat shock 70 kDa protein 7	HSPA6;HSPA7	1.17E+08	2.83E+07	4.61E+07	4.52E+07	0.00E+00	0.00E+00
Calpain-2 catalytic subunit	CAPN2	5.50E+07	6.54E+06	8.71E+06	2.51E+07	4.02E+07	5.69E+07
T-complex protein 1 subunit alpha	TCP1	1.05E+08	6.18E+06	2.88E+07	5.56E+07	1.32E+08	1.33E+08
E3 ubiquitin-protein ligase TRIM21	TRIM21	6.69E+07	8.69E+06	1.30E+07	5.70E+07	7.85E+07	6.81E+07
Filamin-A	FLNA	2.63E+06	5.14E+06	8.54E+05	2.53E+08	6.49E+08	4.47E+08

Heterogeneous nuclear ribonucleoproteins A2/B1	HNRNPA2B1	1.33E+08	5.49E+08	8.04E+07	2.64E+09	2.61E+09	2.91E+09
Splicing factor, proline- and glutamine-rich	SFPQ	6.26E+07	1.67E+07	6.22E+06	1.98E+09	1.98E+09	2.27E+09
40S ribosomal protein S12	RPS12	1.60E+07	2.63E+06	3.17E+06	5.40E+07	6.05E+07	5.84E+07
ATP synthase subunit alpha, mitochondrial	ATP5A1	4.71E+07	1.46E+06	2.35E+07	3.63E+07	4.20E+07	4.41E+07
60S ribosomal protein L13	RPL13	2.31E+07	5.22E+06	1.27E+07	2.86E+07	1.87E+07	0.00E+00
Heterogeneous nuclear ribonucleoprotein H3	HNRNPH3	3.39E+07	5.45E+07	1.56E+07	4.47E+08	4.71E+08	5.27E+08
60S ribosomal protein L4	RPL4	1.59E+07	2.45E+06	6.77E+06	1.53E+08	1.95E+08	1.46E+08
40S ribosomal protein S19	RPS19	3.01E+07	3.03E+06	7.74E+06	8.03E+07	5.45E+07	1.06E+08
40S ribosomal protein S10	RPS10;RPS10-NUDT3	5.86E+06	3.90E+06	1.09E+07	6.59E+07	3.78E+07	5.22E+07
T-complex protein 1 subunit gamma	CCT3	1.08E+08	4.53E+06	2.68E+07	1.19E+08	1.29E+08	1.40E+08
Serpin H1	SERPINH1	4.24E+08	2.24E+07	2.84E+08	6.26E+08	9.12E+08	9.45E+08
T-complex protein 1 subunit delta	CCT4	4.01E+07	5.23E+06	9.95E+06	1.10E+08	1.55E+08	1.25E+08
Heterogeneous nuclear ribonucleoprotein A3	HNRNPA3	1.89E+07	1.28E+07	1.57E+06	1.03E+09	1.26E+09	1.32E+09
Heterogeneous nuclear ribonucleoprotein F	HNRNPF	2.21E+07	5.93E+06	5.89E+06	5.91E+07	1.09E+08	1.29E+08
Heterogeneous nuclear ribonucleoprotein H2	HNRNPH2	5.67E+07	8.12E+07	2.30E+07	9.97E+07	2.24E+08	2.81E+08
Actin, cytoplasmic 2; 1	ACTG1;ACTB	1.21E+09	1.42E+08	2.68E+08	8.10E+08	9.43E+08	1.09E+09
Heterogeneous nuclear ribonucleoprotein K	HNRNPK	4.71E+07	1.38E+07	2.00E+07	3.74E+08	5.59E+08	5.38E+08
40S ribosomal protein S16	RPS16	2.46E+07	2.49E+06	1.65E+07	1.00E+08	1.53E+08	1.28E+08
40S ribosomal protein S18	RPS18	8.84E+06	5.48E+06	6.80E+06	1.55E+08	2.38E+08	2.05E+08
40S ribosomal protein S4, X isoform; Y isoform 2; Y isoform 1	RPS4X;RPS4Y1;RPS4Y2	3.03E+07	3.52E+06	1.66E+06	4.03E+07	1.28E+08	7.27E+07
40S ribosomal protein S6	RPS6	6.48E+06	2.84E+06	6.50E+06	7.41E+07	5.94E+07	7.69E+07
Histone H4	HIST1H4A	2.21E+07	5.78E+06	5.35E+06	1.16E+07	1.64E+07	4.16E+07
60S ribosomal protein L23	RPL23	3.16E+07	2.16E+06	1.70E+07	5.25E+07	7.57E+07	7.47E+07
40S ribosomal protein S28	RPS28	2.20E+07	6.51E+06	7.22E+06	0.00E+00	0.00E+00	0.00E+00
40S ribosomal protein S21	RPS21	6.75E+06	6.20E+06	2.93E+06	1.31E+07	4.03E+07	5.06E+07
Nuclease-sensitive element-binding protein 1;Y-box-binding protein 2;Y-box-binding protein 3	YBX1;YBX2;YBX3	5.58E+07	1.57E+07	1.35E+07	9.48E+07	1.38E+08	1.42E+08
Actin, alpha skeletal muscle; cardiac muscle 1; gamma-enteric smooth muscle; aortic smooth muscle	ACTA1;ACTC1;ACTG2;ACTA2	1.72E+08	3.52E+07	6.46E+07	1.07E+08	1.79E+08	1.48E+08
Putative elongation factor 1-alpha-like 3; 1-alpha 1; 1-alpha 2	EEF1A1P5;EEF1A1;EEF1A2	5.13E+08	6.73E+07	2.80E+08	3.42E+08	6.36E+08	7.57E+08
Tubulin alpha-1B chain; alpha-1C chain	TUBA1B;TUBA1C	2.46E+09	6.95E+08	1.48E+09	7.33E+09	1.13E+10	1.06E+10
Synapse-associated protein 1	SYAP1	1.49E+07	2.77E+06	4.22E+06	0.00E+00	0.00E+00	0.00E+00
T-complex protein 1 subunit eta	CCT7	7.70E+06	4.44E+06	2.80E+06	2.81E+07	1.52E+07	1.57E+07
ADP-ribosylation factor GTPase-activating protein 3	ARFGAP3	6.94E+07	1.05E+07	2.77E+07	0.00E+00	0.00E+00	0.00E+00
GTP-binding protein SAR1a; SAR1b	SAR1A;SAR1B	1.11E+07	2.18E+06	6.96E+06	0.00E+00	0.00E+00	0.00E+00

Supplementary table 7: List of 37 protein hits identified in isotype control samples of HeLa cell extracts

		Isotype control			Kpnβ1 pull-down		
Protein name	Gene name	Intensity (1)	Intensity (2)	Intensity (3)	Intensity (1)	Intensity (2)	Intensity (3)
Clathrin heavy chain	CLTC	4.84E+07	2.66E+07	5.42E+07	8.26E+09	4.56E+09	6.55E+09
Heterogeneous nuclear ribonucleoprotein M	HNRNPM	7.92E+07	1.40E+07	1.39E+08	1.74E+09	8.01E+08	1.44E+09
40S ribosomal protein SA	RPSA	1.10E+07	1.21E+06	6.17E+06	6.62E+08	2.61E+08	4.32E+08
Matrin-3	MATR3	1.80E+07	9.16E+06	1.97E+07	7.90E+08	3.71E+08	5.79E+08
Polypyrimidine tract-binding protein 1	PTBP1	6.98E+06	2.28E+06	5.97E+06	3.25E+08	1.76E+08	3.36E+08
Heterogeneous nuclear ribonucleoproteins C1/C2	HNRNPC	1.28E+07	5.80E+06	1.66E+07	5.89E+08	2.81E+08	4.47E+08
Protein transport protein Sec31A	SEC31A	2.63E+07	8.33E+06	2.76E+07	8.03E+08	3.69E+08	6.25E+08
Tubulin alpha-1C chain;Tubulin alpha-1B chain;Tubulin alpha-8 chain	TUBA1C;TUBA1B;TUBA8	1.31E+08	3.28E+07	1.22E+08	5.07E+09	2.29E+09	3.58E+09
CAD protein	CAD	3.36E+06	5.96E+06	3.21E+06	1.31E+09	6.74E+08	1.10E+09
Heterogeneous nuclear ribonucleoprotein H	HNRNPH1	1.08E+08	3.67E+07	1.28E+08	2.20E+09	1.41E+09	2.34E+09
DAZ-associated protein 1	DAZAP1	2.35E+07	1.42E+07	2.89E+07	2.05E+08	7.33E+07	1.86E+08
Heat shock protein beta-1	HSPB1	2.52E+08	4.74E+06	3.47E+08	2.06E+08	1.45E+08	2.26E+08
Keratin, type I cytoskeletal 18	KRT18	1.44E+07	4.40E+06	3.20E+07	1.27E+08	1.05E+08	7.05E+07
Tubulin beta chain	TUBB	1.74E+08	2.24E+07	2.62E+08	7.16E+09	3.71E+09	5.93E+09
Vimentin	VIM	8.07E+06	2.16E+06	8.86E+06	9.01E+07	4.93E+07	3.28E+07
Heterogeneous nuclear ribonucleoprotein A1;A1-like 2	HNRNPA1;HNRNPA1L2	1.44E+08	6.16E+07	1.68E+08	3.63E+09	1.96E+09	3.43E+09
60 kDa heat shock protein, mitochondrial	HSPD1	2.97E+07	1.76E+07	5.26E+07	1.86E+08	3.16E+08	2.09E+08
78 kDa glucose-regulated protein	HSPA5	4.44E+07	8.95E+05	5.98E+07	7.43E+07	3.00E+07	5.93E+07
Polyadenylate-binding protein 1;Polyadenylate-binding protein	PABPC1	5.76E+06	1.33E+06	2.12E+07	1.14E+09	5.31E+08	9.69E+08
Replication protein A 32 kDa subunit	RPA2	4.38E+06	1.35E+06	3.33E+06	7.78E+07	4.58E+07	7.34E+07
Nucleolin	NCL	2.27E+06	1.46E+06	7.40E+05	1.31E+08	4.35E+07	6.10E+07
Heterogeneous nuclear ribonucleoproteins A2/B1	HNRNPA2B1	3.58E+08	1.97E+08	3.89E+08	7.64E+09	3.74E+09	6.79E+09
40S ribosomal protein S12	RPS12	6.01E+06	9.79E+06	3.29E+07	1.65E+08	9.24E+07	1.63E+08
RNA-binding protein FUS	FUS	1.88E+07	8.38E+06	2.52E+07	1.00E+09	4.45E+08	8.11E+08
40S ribosomal protein S10;Putative 40S ribosomal protein S10-like	RPS10;RPS10-NUDT3;RPS10P5	2.86E+06	2.68E+06	7.63E+06	1.60E+08	8.50E+07	2.03E+08
Heterogeneous nuclear ribonucleoprotein A3	HNRNPA3	3.34E+06	1.74E+06	7.88E+06	1.44E+09	7.84E+08	1.40E+09
Heterogeneous nuclear ribonucleoprotein F	HNRNPF	2.70E+06	3.21E+06	9.92E+06	2.44E+08	9.63E+07	2.26E+08
Actin, cytoplasmic 2;1	ACTG1;ACTB	8.85E+07	1.78E+06	8.71E+07	1.36E+09	6.21E+08	1.01E+09
40S ribosomal protein S3a	RPS3A	1.64E+07	7.31E+06	7.70E+06	1.43E+08	7.03E+07	1.85E+08
Heterogeneous nuclear ribonucleoprotein K	HNRNPK	1.92E+07	7.46E+06	3.01E+07	1.36E+09	6.68E+08	1.14E+09
40S ribosomal protein S7	RPS7	6.97E+06	3.61E+06	7.84E+06	1.12E+08	8.36E+07	1.25E+08
Putative elongation factor 1-alpha-like 3;1-alpha 1;1-alpha 2	EEF1A1P5;EEF1A1;EEF1A2	4.64E+07	2.00E+06	7.80E+07	4.92E+08	2.48E+08	5.76E+08
Tubulin beta-4B chain	TUBB4B	4.80E+07	6.64E+05	6.05E+07	1.36E+09	5.28E+08	1.12E+09
Heterogeneous nuclear ribonucleoprotein U	HNRNPU	1.62E+07	3.03E+06	1.44E+07	1.57E+09	8.67E+08	1.42E+09
ATP-dependent RNA helicase A	DHX9	2.86E+07	8.34E+06	2.75E+07	8.53E+08	4.55E+08	6.52E+08

TAR DNA-binding protein 43	TARDBP	2.53E+07	5.72E+06	3.33E+07	2.06E+08	1.23E+08	2.12E+08
Ras GTPase-activating protein-binding protein 2	G3BP2	3.66E+06	2.13E+06	4.20E+06	7.95E+07	4.80E+07	1.03E+08

Supplementary table 8: List of 69 protein hits identified in isotype control samples of WHCO5 cell extracts

		Isotype control			KpnB1 pull-down		
Protein name	Gene name	Intensity (1)	Intensity (2)	Intensity (3)	Intensity (1)	Intensity (2)	Intensity (3)
40S ribosomal protein S24	RPS24	1.10E+06	3.20E+06	4.83E+06	4.42E+07	5.45E+07	7.34E+07
Heterogeneous nuclear ribonucleoprotein M	HNRNPM	1.08E+08	1.74E+08	8.96E+07	1.06E+09	1.13E+09	8.54E+08
Ig gamma-1 chain C region;Ig gamma-3 chain C region	IGHG1;IGHG3	2.00E+07	1.05E+08	5.12E+07	3.37E+09	9.27E+08	5.14E+09
40S ribosomal protein SA	RPSA	1.09E+07	1.24E+07	2.95E+07	2.07E+08	3.23E+08	1.87E+08
Calpastatin	CAST	4.55E+07	1.27E+08	3.28E+07	3.36E+07	7.39E+06	6.36E+07
Calpain small subunit 1	CAPNS1	1.93E+07	1.25E+07	1.56E+07	2.14E+07	2.70E+07	1.08E+07
Heat shock 70 kDa protein 1B;1A	HSPA1B;HSPA1A	3.97E+07	7.62E+07	1.10E+08	4.08E+08	5.93E+08	4.08E+08
Matrin-3	MATR3	4.55E+06	1.52E+07	2.20E+06	1.94E+08	2.99E+08	1.72E+08
60S ribosomal protein L23a	RPL23A	6.78E+06	6.56E+06	1.52E+07	7.74E+06	3.06E+07	6.83E+07
60S ribosomal protein L14	RPL14	9.04E+06	9.95E+06	1.10E+07	4.05E+07	5.21E+07	4.41E+07
Ribosomal protein L15;60S ribosomal protein L15	RPL15	3.28E+06	4.03E+06	5.59E+06	2.32E+07	4.74E+07	2.29E+07
60S ribosomal protein L8	RPL8	7.61E+06	5.88E+06	7.50E+06	5.71E+07	7.19E+07	7.84E+07
Cofilin-1;Cofilin-2	CFL1;CFL2	6.76E+06	3.53E+07	4.77E+07	1.07E+07	9.62E+06	1.11E+07
Tubulin alpha-1C chain;alpha-1B chain;alpha-1A chain;alpha-3C/D chain	TUBA1C;TUBA1B;TUBA1A;TUBA3C	1.74E+08	4.51E+08	4.58E+08	4.80E+09	8.72E+09	6.27E+09
Heterogeneous nuclear ribonucleoprotein A1;A1-like 2	HNRNPA1;HNRNPA1L2	9.06E+07	1.22E+08	7.82E+07	1.89E+09	3.47E+09	3.14E+09
Heterogeneous nuclear ribonucleoprotein H	HNRNPH1	4.94E+07	8.34E+07	5.30E+07	1.15E+09	1.71E+09	1.20E+09
Small nuclear ribonucleoprotein-associated protein N;B	SNRPN;SNRPB	2.19E+06	3.73E+06	8.13E+06	2.04E+08	3.30E+08	1.80E+08
Heterogeneous nuclear ribonucleoprotein L	HNRNPL	3.04E+06	3.19E+06	9.24E+06	5.58E+08	8.13E+08	6.51E+08
Glyceraldehyde-3-phosphate dehydrogenase	GAPDH	3.32E+06	2.17E+07	3.15E+07	5.94E+07	3.13E+07	1.64E+07
Heat shock protein beta-1	HSPB1	4.26E+08	7.78E+08	5.80E+08	8.20E+08	8.68E+08	7.06E+08
Keratin, type I cytoskeletal 18	KRT18	5.11E+07	2.30E+08	1.10E+08	4.22E+08	1.04E+09	7.46E+08
Calpain-1 catalytic subunit	CAPN1	1.65E+08	2.10E+08	8.75E+07	1.36E+08	5.03E+07	1.48E+08
Tubulin beta chain	TUBB	3.02E+08	8.13E+08	4.33E+08	7.78E+09	1.36E+10	8.45E+09
40S ribosomal protein S17	RPS17	1.09E+06	2.18E+06	3.90E+06	7.55E+07	4.45E+07	5.43E+07
Glutathione S-transferase P	GSTP1	6.63E+06	4.00E+06	1.44E+07	0.00E+00	0.00E+00	0.00E+00
Thioredoxin	TXN	1.92E+06	3.00E+06	1.40E+07	0.00E+00	0.00E+00	0.00E+00
60 kDa heat shock protein, mitochondrial	HSPD1	1.26E+06	2.13E+07	8.62E+06	3.51E+06	1.33E+07	7.63E+06
78 kDa glucose-regulated protein	HSPA5	1.45E+06	1.07E+07	4.61E+06	0.00E+00	3.72E+06	0.00E+00
Heat shock cognate 71 kDa protein	HSPA8	1.21E+08	2.90E+08	4.74E+08	5.88E+08	7.67E+08	5.63E+08
Macrophage migration inhibitory factor	MIF	2.08E+07	3.49E+07	3.43E+07	6.01E+06	0.00E+00	0.00E+00
E3 ubiquitin-protein ligase TRIM21	TRIM21	2.16E+06	6.74E+06	6.68E+06	3.95E+07	1.04E+08	6.02E+07
Heterogeneous nuclear ribonucleoproteins A2/B1	HNRNPA2B1	1.91E+08	3.00E+08	1.03E+08	3.66E+09	5.83E+09	4.77E+09
Splicing factor, proline- and glutamine-rich	SFPQ	6.35E+06	9.49E+06	1.54E+07	1.65E+09	2.09E+09	1.72E+09
40S ribosomal protein S3	RPS3	5.92E+06	5.56E+06	3.65E+07	2.07E+08	2.78E+08	2.07E+08
40S ribosomal protein S12	RPS12	1.06E+07	2.05E+07	7.93E+06	1.11E+08	1.76E+08	1.26E+08

60S ribosomal protein L13	RPL13	1.57E+07	1.80E+07	7.26E+06	5.12E+07	4.38E+07	1.00E+08
60S ribosomal protein L4	RPL4	1.12E+07	1.19E+07	2.03E+07	1.47E+08	1.66E+08	2.17E+08
60S ribosomal protein L3	RPL3	8.65E+06	1.83E+07	1.41E+07	6.19E+07	9.28E+07	1.16E+08
60S ribosomal protein L5	RPL5	1.27E+06	1.41E+06	2.77E+06	3.27E+07	2.61E+07	1.87E+07
40S ribosomal protein S10	RPS10;RPS10-NUDT3	8.34E+06	1.05E+07	1.77E+07	1.17E+08	1.81E+08	1.38E+08
Heterogeneous nuclear ribonucleoprotein A3	HNRNPA3	6.15E+06	6.75E+06	4.82E+06	1.60E+09	1.95E+09	1.78E+09
Activated RNA polymerase II transcriptional coactivator p15	SUB1	1.40E+06	1.43E+07	1.42E+07	0.00E+00	0.00E+00	0.00E+00
Heterogeneous nuclear ribonucleoprotein H2	HNRNPH2	2.06E+07	4.21E+07	3.69E+07	4.44E+08	6.92E+08	5.09E+08
Actin, cytoplasmic 2;1	ACTG1;ACTB	4.42E+07	1.09E+08	2.51E+08	2.69E+08	4.85E+08	3.69E+08
40S ribosomal protein S20	RPS20	5.66E+06	1.19E+06	2.37E+07	4.96E+07	1.11E+08	1.10E+08
60S ribosomal protein L27	RPL27	3.25E+06	1.50E+07	2.67E+07	1.30E+08	9.27E+07	7.89E+07
Heterogeneous nuclear ribonucleoprotein K	HNRNPK	1.51E+06	1.30E+07	1.08E+07	5.92E+08	9.67E+08	7.32E+08
40S ribosomal protein S7	RPS7	6.80E+06	1.32E+07	1.12E+07	9.65E+07	1.37E+08	1.23E+08
40S ribosomal protein S8	RPS8	1.19E+07	8.62E+06	1.12E+07	9.19E+07	1.41E+08	1.36E+08
40S ribosomal protein S16	RPS16	1.37E+07	1.41E+07	3.47E+07	1.74E+08	2.19E+08	2.36E+08
40S ribosomal protein S14	RPS14	4.10E+06	8.18E+06	1.39E+07	1.34E+08	8.41E+07	7.96E+07
40S ribosomal protein S23	RPS23	5.56E+06	8.02E+06	9.34E+06	3.27E+07	4.27E+07	3.30E+07
40S ribosomal protein S18	RPS18	2.85E+06	9.03E+06	1.51E+07	2.59E+08	3.85E+08	2.94E+08
Small nuclear ribonucleoprotein Sm D3	SNRPD3	4.36E+06	6.80E+06	1.04E+07	1.78E+08	3.30E+08	1.88E+08
60S ribosomal protein L7a	RPL7A	1.22E+07	6.50E+06	1.67E+07	8.51E+07	1.11E+08	8.98E+07
40S ribosomal protein S25	RPS25	5.09E+06	7.24E+06	1.13E+07	1.51E+08	1.31E+08	1.31E+08
60S ribosomal protein L10a	RPL10A	3.78E+06	2.76E+06	6.61E+06	4.86E+07	2.16E+07	7.27E+07
60S ribosomal protein L11	RPL11	4.97E+06	1.18E+07	8.14E+06	5.95E+07	4.66E+07	3.84E+07
60S ribosomal protein L38	RPL38	3.42E+06	6.48E+06	1.20E+07	5.15E+07	6.00E+07	6.84E+07
40S ribosomal protein S21	RPS21	1.04E+07	1.13E+07	1.87E+07	6.69E+07	6.50E+07	9.37E+07
Nuclease-sensitive element-binding protein 1	YBX1	6.45E+06	1.74E+07	2.60E+07	1.05E+08	1.57E+08	1.53E+08
Actin, alpha skeletal muscle;cardiac muscle 1;gamma-enteric smooth muscle;aortic smooth muscle	ACTA1;ACTC1;ACTG2;ACTA2	1.45E+07	3.00E+07	6.35E+07	0.00E+00	6.73E+07	5.25E+07
Putative elongation factor 1-alpha-like 3;1-alpha 1;1-alpha;1-alpha 2	EEF1A1P5;EEF1A1;EEF1A2	2.60E+07	1.09E+08	1.45E+08	4.13E+08	4.12E+08	4.04E+08
Tubulin beta-4B chain	TUBB4B	8.47E+07	1.89E+08	6.15E+07	1.60E+09	2.37E+09	1.67E+09
60S ribosomal protein L6	RPL6	1.61E+07	1.02E+07	1.25E+07	1.22E+08	1.49E+08	1.46E+08
TAR DNA-binding protein 43	TARDBP	9.23E+06	1.51E+07	5.74E+06	9.74E+07	1.51E+08	9.97E+07
Src substrate cortactin	CTTN	7.83E+06	4.99E+06	3.43E+06	1.51E+08	4.40E+07	2.37E+08
Synapse-associated protein 1	SYAP1	1.08E+07	1.94E+07	4.97E+06	0.00E+00	0.00E+00	0.00E+00
RuvB-like 1	RUVBL1	2.87E+06	3.70E+06	2.49E+06	1.84E+07	4.98E+07	3.26E+07

Supplementary table 9: List of 90 protein hits identified in isotype control samples of KYSE30 cell extracts

			Isotype control			KpnB1 pull-down	
Protein name	Gene name	Intensity (1)	Intensity (2)	Intensity (3)	Intensity (1)	Intensity (2)	Intensity (3)
Clathrin heavy chain;Clathrin heavy chain 1	CLTC	2.50E+07	1.49E+07	1.70E+07	3.58E+09	2.85E+09	3.29E+09
60S ribosomal protein L17	RPL17	1.63E+07	1.53E+07	1.79E+07	1.52E+08	3.68E+08	1.37E+08
Heterogeneous nuclear ribonucleoprotein M	HNRNPM	1.36E+08	6.72E+07	1.14E+08	3.30E+09	3.44E+09	4.39E+09
40S ribosomal protein SA	RPSA	3.51E+07	4.91E+07	5.93E+07	2.26E+09	2.05E+09	2.56E+09
Calpastatin	CAST	1.81E+07	9.04E+06	2.15E+07	0.00E+00	0.00E+00	0.00E+00
ADP-ribosylation factor GTPase-activating protein 2	ARFGAP2	5.25E+06	1.87E+06	1.26E+07	0.00E+00	0.00E+00	0.00E+00
Heat shock 70 kDa protein 1B;Heat shock 70 kDa protein 1A	HSPA1B;HSPA1A	1.08E+08	4.60E+07	7.24E+07	2.27E+09	2.04E+09	2.16E+09
Matrin-3	MATR3	4.03E+06	1.30E+07	4.44E+06	3.18E+08	3.10E+08	4.60E+08
Fatty acid synthase	FASN	3.75E+06	1.79E+07	3.32E+06	1.14E+09	1.12E+09	1.13E+09
Polypyrimidine tract-binding protein 1	PTBP1	2.33E+07	1.35E+07	2.98E+07	6.22E+08	6.11E+08	7.76E+08
60S ribosomal protein L23a	RPL23A	1.76E+07	1.94E+07	1.86E+07	3.53E+08	3.96E+08	5.38E+08
Small nuclear ribonucleoprotein G;G-like protein 15	SNRPG;SNRPGP15	5.02E+06	5.15E+05	4.16E+06	9.25E+07	8.62E+07	9.04E+07
	HNRNPC	1.51E+07	1.81E+07	1.65E+07	4.39E+08	5.40E+08	5.75E+08
Eukaryotic translation initiation factor 3 subunitH	EIF3H	1.08E+06	1.77E+07	9.31E+06	5.41E+08	6.31E+08	8.61E+08
60S ribosomal protein L37a	RPL37A	8.64E+06	1.00E+07	1.10E+07	5.28E+07	6.40E+07	6.38E+07
60S ribosomal protein L24	RPL24	4.95E+06	2.86E+06	7.39E+06	2.88E+08	1.88E+08	3.43E+08
60S ribosomal protein L9	RPL9	3.86E+06	6.23E+06	3.33E+06	2.40E+08	2.83E+08	3.79E+08
Protein transport protein Sec31A	SEC31A	4.30E+06	1.42E+07	5.04E+06	1.91E+08	1.78E+08	1.90E+08
60S ribosomal protein L14	RPL14	5.21E+06	3.82E+06	8.33E+06	1.44E+08	1.99E+08	2.07E+08
Ribosomal protein L15	RPL15	2.32E+06	5.52E+05	2.97E+06	2.34E+08	2.11E+08	2.02E+08
CAD protein	CAD	3.59E+06	7.68E+06	2.47E+06	2.35E+08	1.99E+08	1.72E+08
Heterogeneous nuclear ribonucleoprotein A1;A1-like 2	HNRNPA1;HNRNPA1L2	1.63E+08	9.46E+07	1.59E+08	2.63E+09	2.58E+09	3.30E+09
Heterogeneous nuclear ribonucleoprotein H	HNRNPH1	6.62E+07	9.27E+07	8.71E+07	1.08E+09	1.00E+09	9.50E+08
Inosine-5-monophosphate dehydrogenase 2	IMPDH2	1.40E+06	3.58E+06	1.45E+06	1.96E+07	2.03E+07	1.44E+06
Eukaryotic translation initiation factor 3 subunit M	EIF3M	2.19E+06	2.12E+06	1.56E+06	2.54E+07	2.28E+07	2.85E+07
DAZ-associated protein 1	DAZAP1	1.59E+07	1.15E+07	2.49E+07	8.89E+07	8.23E+07	8.95E+07
40S ribosomal protein S15	RPS15	1.41E+07	1.52E+07	1.70E+07	2.37E+08	3.20E+08	3.00E+08
Heterogeneous nuclear ribonucleoprotein Q	SYNCRIP	7.95E+06	9.50E+06	9.46E+06	2.54E+08	3.83E+08	3.58E+08
Filamin-B	FLNB	1.68E+07	4.16E+07	3.31E+07	1.51E+09	1.72E+09	1.90E+09
Glyceraldehyde-3-phosphate dehydrogenase	GAPDH	6.76E+06	5.37E+06	1.54E+06	3.03E+07	6.26E+07	3.52E+07
Heat shock protein beta-1	HSPB1	2.22E+08	1.18E+08	2.50E+08	1.28E+09	1.32E+09	1.32E+09
60S acidic ribosomal protein P0	RPLP0	8.44E+06	1.13E+07	1.07E+07	9.10E+08	7.90E+08	8.50E+08
Keratin, type I cytoskeletal 18	KRT18	5.35E+07	7.98E+07	3.31E+07	2.19E+09	2.27E+09	2.25E+09
Nucleophosmin	NPM1	7.54E+07	6.16E+07	9.39E+07	2.23E+09	2.08E+09	2.49E+09
Calpain-1 catalytic subunit	CAPN1	6.90E+06	1.48E+07	3.37E+06	4.07E+07	2.80E+07	3.46E+07
Tubulin beta chain	TUBB	5.59E+08	3.42E+08	5.13E+08	2.94E+10	2.40E+10	2.98E+10
Heat shock protein HSP 90-beta	HSP90AB1	2.02E+06	4.03E+06	7.33E+06	4.14E+08	3.68E+08	4.49E+08
40S ribosomal protein S17	RPS17	9.63E+06	4.24E+06	2.18E+06	1.71E+08	2.59E+08	2.50E+08
60 kDa heat shock protein, mitochondrial	HSPD1	4.33E+07	2.20E+07	3.42E+07	1.18E+08	1.77E+08	1.97E+08
78 kDa glucose-regulated protein	HSPA5	1.17E+07	3.50E+06	2.80E+06	3.90E+07	1.48E+07	4.45E+07
Heat shock cognate 71 kDa protein	HSPA8	1.56E+08	9.34E+07	1.34E+08	3.64E+09	3.35E+09	4.08E+09
Ribose-phosphate pyrophosphokinase 2	PRPS2	2.34E+06	6.17E+06	3.79E+06	1.38E+08	8.40E+07	1.80E+08

Macrophage migration inhibitory factor	MIF	2.14E+07	1.06E+07	1.84E+07	0.00E+00	8.89E+06	9.23E+06
40S ribosomal protein S2	RPS2	1.91E+07	3.28E+07	2.85E+07	7.11E+08	8.09E+08	9.53E+08
Y-box-binding protein 3	YBX3	1.39E+07	9.79E+06	3.20E+06	2.20E+08	2.08E+08	3.44E+08
T-complex protein 1 subunit alpha	TCP1	1.80E+06	6.16E+06	5.67E+06	6.28E+07	9.12E+07	1.77E+08
Nucleolin	NCL	2.20E+07	3.33E+07	5.43E+06	1.87E+09	1.78E+09	2.07E+09
Filamin-A	FLNA	5.69E+06	9.27E+06	3.21E+06	1.83E+09	1.95E+09	2.13E+09
Heterogeneous nuclear ribonucleoproteins A2/B1	HNRNPA2B1	4.31E+08	2.96E+08	4.25E+08	5.31E+09	5.97E+09	6.87E+09
40S ribosomal protein S3	RPS3	2.31E+06	1.49E+06	4.21E+06	1.21E+09	1.12E+09	1.36E+09
40S ribosomal protein S12	RPS12	4.46E+07	6.10E+07	5.93E+07	4.76E+08	4.03E+08	5.08E+08
RNA-binding protein FUS	FUS	2.32E+07	8.36E+06	2.05E+07	3.70E+08	4.26E+08	6.28E+08
40S ribosomal protein S19	RPS19	1.16E+07	6.60E+06	1.01E+07	8.55E+08	6.18E+08	9.93E+08
60S ribosomal protein L3	RPL3	1.42E+07	2.07E+07	3.05E+07	5.39E+08	5.12E+08	7.18E+08
Heterogeneous nuclear ribonucleoprotein F	HNRNPF	1.87E+07	8.82E+06	1.06E+07	1.38E+08	2.75E+08	1.18E+08
Transitional endoplasmic reticulum ATPase	VCP	1.18E+07	3.05E+07	1.33E+07	1.38E+09	1.35E+09	1.65E+09
Heterogeneous nuclear ribonucleoprotein H2	HNRNPH2	2.09E+07	1.79E+07	2.01E+07	2.03E+08	8.00E+07	8.87E+07
Actin, cytoplasmic 2;1	ACTG1;ACTB	6.22E+07	3.22E+07	4.03E+07	1.36E+09	1.17E+09	1.41E+09
40S ribosomal protein S3a	RPS3A	3.37E+07	1.75E+07	3.63E+07	2.38E+08	2.44E+08	3.64E+08
60S ribosomal protein L27	RPL27	1.66E+06	1.06E+06	1.21E+07	5.27E+08	6.07E+08	7.11E+08
Heterogeneous nuclear ribonucleoprotein K	HNRNPK	1.79E+07	4.33E+07	2.76E+07	2.82E+09	2.98E+09	3.54E+09
40S ribosomal protein S7	RPS7	1.09E+07	4.99E+06	1.77E+07	4.17E+08	5.46E+08	3.64E+08
40S ribosomal protein S16	RPS16	1.80E+07	5.47E+06	9.08E+06	5.90E+08	7.06E+08	7.17E+08
40S ribosomal protein S23	RPS23	4.03E+06	2.29E+06	5.07E+06	1.01E+08	8.23E+07	1.08E+08
40S ribosomal protein S18	RPS18	4.11E+06	2.39E+06	1.37E+07	7.98E+08	6.67E+08	9.08E+08
60S ribosomal protein L23	RPL23	1.16E+07	1.13E+07	3.11E+06	2.35E+08	2.81E+08	2.52E+08
40S ribosomal protein S28	RPS28	1.74E+07	6.64E+06	2.46E+07	3.14E+08	5.71E+08	6.94E+08
60S ribosomal protein L10a	RPL10A	2.80E+06	4.62E+06	4.73E+06	1.44E+08	2.26E+08	2.36E+08
60S ribosomal protein L11	RPL11	2.83E+06	7.99E+06	3.68E+06	2.86E+08	1.46E+08	2.93E+08
60S ribosomal protein L8	RPL8	1.90E+06	1.21E+06	4.08E+06	1.29E+08	2.56E+08	3.04E+08
60S ribosomal protein L38	RPL38	6.28E+06	2.64E+06	8.24E+06	1.11E+08	1.32E+08	2.02E+08
40S ribosomal protein S21	RPS21	2.19E+07	1.79E+07	3.43E+07	5.84E+08	6.54E+08	8.16E+08
Actin, alpha skeletal muscle;cardiac muscle 1;gamma-enteric smooth muscle;aortic smooth muscle	ACTA1;ACTC1;ACTG2;ACTA2	1.82E+07	7.99E+06	1.16E+07	2.18E+08	1.70E+08	2.19E+08
Putative elongation factor 1-alpha-like 3;1-alpha 1;1-alpha;1-alpha 2	EEF1A1P5;EEF1A1;EEF1A2	4.75E+07	3.38E+07	7.37E+07	6.57E+08	1.46E+09	1.54E+09
Tubulin alpha-1B chain;alpha-1C chain;alpha-1A chain;alpha-3C/D chain;alpha-8 chain	TUBA1B;TUBA1C;TUBA1A;TUBA3C;TUBA8	5.89E+08	3.52E+08	3.90E+08	2.55E+10	2.01E+10	2.46E+10
Tubulin beta-4B chain	TUBB4B	9.34E+07	9.07E+07	1.03E+08	3.79E+09	3.03E+09	3.91E+09
Heterogeneous nuclear ribonucleoprotein U	HNRNPU	5.30E+06	1.78E+07	2.64E+06	1.70E+09	1.78E+09	2.10E+09
ATP-dependent RNA helicase A	DHX9	2.58E+07	1.55E+07	2.82E+07	3.29E+08	4.52E+08	3.52E+08
Ras GTPase-activating protein-binding protein 1	G3BP1	1.07E+07	8.64E+06	9.25E+06	2.78E+08	4.15E+08	3.73E+08
Eukaryotic translation initiation factor 3 subunit I	EIF3I	9.57E+06	7.68E+06	1.74E+07	9.43E+08	8.33E+08	9.90E+08
Tubulin beta-3 chain	TUBB3	9.44E+06	5.65E+06	7.56E+06	3.73E+08	3.11E+08	3.86E+08
Src substrate cortactin	CTTN	1.25E+07	1.57E+07	1.44E+07	3.58E+08	4.49E+08	5.25E+08
Poly(rC)-binding protein 2	PCBP2	5.81E+06	2.84E+06	5.45E+06	1.34E+08	1.39E+08	1.50E+08
Splicing factor 3B subunit 3	SF3B3	5.16E+06	4.65E+06	5.59E+06	4.13E+08	3.84E+08	3.41E+08
ATP-dependent RNA helicase DDX1	DDX1	2.52E+06	7.44E+06	4.64E+06	8.41E+08	7.06E+08	7.21E+08
Protein-arginine deiminase type-3	PADI3	2.49E+06	5.29E+06	3.11E+06	6.62E+07	7.76E+07	7.97E+07
Pre-mRNA-processing factor 19	PRPF19	2.18E+07	1.47E+07	1.88E+07	1.32E+08	9.93E+07	1.93E+08
RuvB-like 2	RUVBL2	6.32E+06	1.13E+07	6.59E+06	4.43E+08	2.66E+08	3.54E+08
tRNA-splicing ligase RtcB homolog	RTCB	7.68E+06	1.12E+07	1.01E+06	8.17E+08	9.49E+08	1.01E+09

Supplementary table 10A: Results from PANTHER enrichment analysis for 38 common Kpnβ1 binding proteins

GO biological process	# in Homo Sapiens reference list	#	expected	Fold enrichment	p value	False discovery rate (FDR)
RNA metabolic process	1635	24	3.18	7.55	4.53E-18	3.55E-14
RNA processing	868	18	1.69	10.65	5.02E-16	1.57E-12
nucleic acid metabolic process	2276	26	4.42	5.88	2.76E-18	4.32E-14
gene expression	2314	25	4.5	5.56	7.22E-17	3.77E-13
mRNA metabolic process	607	16	1.18	13.56	5.57E-16	1.46E-12
mRNA processing	451	14	0.88	15.91	3.90E-15	6.80E-12
cellular nitrogen compound metabolic process	3573	29	6.94	4.18	1.53E-16	5.98E-13
nucleobase-containing compound metabolic process	2825	26	5.49	4.74	7.96E-16	1.78E-12
heterocycle metabolic process	2999	26	5.83	4.46	3.76E-15	7.37E-12
cellular aromatic compound metabolic process	3050	26	5.93	4.38	5.82E-15	9.12E-12
GO cellular component						
ribonucleoprotein complex	714	22	1.39	15.83	6.47E-22	1.32E-18
nuclear protein-containing complex	1276	22	2.48	8.87	1.20E-16	8.15E-14
nuclear lumen	4494	34	8.73	3.89	3.39E-17	3.46E-14
nucleoplasm	4130	32	8.02	3.99	7.05E-16	3.60E-13
spliceosomal complex	197	10	0.38	26.32	1.50E-13	3.41E-11
organelle lumen	5614	34	10.91	3.12	4.21E-14	1.43E-11
nucleus	7682	36	14.92	2.41	6.09E-12	1.24E-09
protein-containing complex	5771	35	11.21	3.12	6.68E-15	2.73E-12
U1 snRNP	19	5	0.04	> 100	8.75E-10	1.63E-07
spliceosomal snRNP complex	64	6	0.12	50	4.30E-09	7.32E-07
GO molecular function						
RNA binding	1666	30	3.24	9.26	8.32E-25	4.14E-21
nucleic acid binding	4007	35	7.78	4.50	3.43E-20	8.54E-17
mRNA binding	326	14	0.63	22.22	1.32E-15	1.64E-12
heterocyclic compound binding	5983	36	11.62	3.10	1.24E-15	2.06E-12
organic cyclic compound binding	6054	36	11.76	3.06	1.86E-15	1.85E-12
protein binding	14448	38	28.07	1.35	8.89E-07	7.37E-04
mRNA 3'-UTR binding	102	5	0.2	25.00	1.95E-06	1.38E-03
RNA helicase activity	77	4	0.15	26.67	1.81E-05	1.13E-02
ATP-dependent activity, acting on RNA	79	4	0.15	26.67	1.99E-05	1.10E-02
pre-mRNA branch point binding	2	1	0	> 100	2.19E-05	1.09E-02
mRNA 3'-UTR AU-rich region binding	26	3	0.05	60	2.38E-05	1.08E-02

Supplementary table 10B: Gene names in each GO category identified in PANTHER enrichment analysis for 38 common Kpnβ1 binding proteins

GO biological process	Gene names
RNA metabolic process	SNRPD3, HNRNPAB, SMARCC2, EWSR1, SNRPN, DDX17, SF1, SNRNP70, CPSF6, CNOT1, RPA1, SF3B2, FUBP3, HNRNPUL1, CSTF1, DDX5, SNRPD2, HNRNPDL, FUS, NUDT21, HNRNPR, HNRNPA0, DDX3X, SNRPA
RNA processing	SNRPD3, SNRPN, DDX17, SF1, SNRNP70, CPSF6, SF3B2, HNRNPUL1, CSTF1, DDX5, SNRPD2, HNRNPDL, FUS, NUDT21, HNRNPR, HNRNPA0, DDX3X, SNRPA
nucleic acid metabolic process	SNRPD3, RPA1, HNRNPAB, SMARCC2, EWSR1, SNRPN, DDX17, SF1, SNRNP70, CPSF6, CNOT1, SF3B2, FUBP3, HNRNPUL1, CSTF1, DDX5, SNRPD2, HNRNPDL, FUS, NUDT21, HNRNPR, CARM1, ACTL6A, HNRNPA0, DDX3X, SNRPA
gene expression	SNRPD3, NUP93, SMARCC2, EWSR1, SNRPN, RPL18A, DDX17, SF1, SNRNP70, CPSF6, SF3B2, RPS13, FUBP3, HNRNPUL1, CSTF1, DDX5, SNRPD2, HNRNPDL, FUS, NUDT21, HNRNPR, HNRNPA0, DDX3X, SNRPA, RPL31
mRNA metabolic process	SNRPD3, HNRNPAB, SNRPN, DDX17, SF1, SNRNP70, CPSF6, CNOT1, SF3B2, CSTF1, DDX5, SNRPD2, NUDT21, HNRNPR, HNRNPA0, SNRPA
mRNA processing	SNRPD3, SNRPN, DDX17, SF1, SNRNP70, CPSF6, SF3B2, CSTF1, DDX5, SNRPD2, NUDT21, HNRNPR, HNRNPA0, SNRPA
cellular nitrogen compound metabolic process	SNRPD3, RPA1, HNRNPAB, SMARCC2, EWSR1, SNRPN, RPL18A, DDX17, SF1, SNRNP70, CPSF6, CNOT1, SF3B2, RPS13, FUBP3, HNRNPUL1, CSTF1, DDX5, SNRPD2, HNRNPDL, FUS, NUDT21, HNRNPR, CARM1, ACTL6A, HNRNPA0, DDX3X, SNRPA
nucleobase-containing compound metabolic process	SNRPD3, RPA1, HNRNPAB, SMARCC2, EWSR1, SNRPN, DDX17, SF1, SNRNP70, CPSF6, CNOT1, SF3B2, FUBP3, HNRNPUL1, CSTF1, DDX5, SNRPD2, HNRNPDL, FUS, NUDT21, HNRNPR, CARM1, ACTL6A, HNRNPA0, DDX3X, SNRPA
heterocycle metabolic process	SNRPD3, RPA1, HNRNPAB, SMARCC2, EWSR1, SNRPN, DDX17, SF1, SNRNP70, CPSF6, CNOT1, SF3B2, FUBP3, HNRNPUL1, CSTF1, DDX5, SNRPD2, HNRNPDL, FUS, NUDT21, HNRNPR, CARM1, ACTL6A, HNRNPA0, DDX3X, SNRPA
cellular aromatic compound metabolic process	SNRPD3, RPA1, HNRNPAB, SMARCC2, EWSR1, SNRPN, DDX17, SF1, SNRNP70, CPSF6, CNOT1, SF3B2, FUBP3, HNRNPUL1, CSTF1, DDX5, SNRPD2, HNRNPDL, FUS, NUDT21, HNRNPR, CARM1, ACTL6A, HNRNPA0, DDX3X, SNRPA

GO cellular component	Gene names
ribonucleoprotein complex	SNRPD3, HNRNPAB, SNRPN, RPL18A, DDX17, SF1, SNRNP70, CPSF6, SF3B2, DDX6, RPS13, FUBP3, HNRNPUL1, DDX5, SNRPD2, HNRNPDL, ELAVL1, HNRNPR, HNRNPA0, SNRPA, RPL31
nuclear protein-containing complex	SNRPD3, RPA1, HNRNPAB, SMARCC2, NUP93, SNRPN, SF1, SNRNP70, CPSF6, SF3B2, CSTF1, DDX5, SNRPD2, HNRNPDL, NUDT21, HNRNPR, SEC13, ACTL6A, KPNB1, SNRPA
nuclear lumen	SNRPD3, RPA1, HNRNPAB, NUP93, SMARCC2, EWSR1, SNRPN, DDX17, SF1, PSPC1, SNRNP70, CPSF6, RPA1, SF3B2, RPS13, FUBP3, HNRNPUL1, CSTF1, DDX5, SNRPD2, HNRNPDL, FUS, ELAVL1, NUDT21, HNRNPR, SEC13, CARM1, ACTL6A, HNRNPA0, DDX3X, KPNB1, TUBG1, SNRPA
nucleoplasm	SNRPD3, RPA1, HNRNPAB, SMARCC2, EWSR1, SNRPN, DDX17, SF1, PSPC1, SNRNP70, CPSF6, SF3B2, RPS13, FUBP3, HNRNPUL1, CSTF1, DDX5, SNRPD2, HNRNPDL, FUS, ELAVL1, NUDT21, HNRNPR, SEC13, CARM1, ACTL6A, HNRNPA0, DDX3X, KPNB1, SNRPA
spliceosomal complex	SNRPD3, SNRPN, SF1, SNRNP70, SF3B2, DDX5, SNRPD2, HNRNPDL, HNRNPR, SNRPA
organelle lumen	SNRPD3, RPA1, HNRNPAB, NUP93, SMARCC2, EWSR1, SNRPN, DDX17, SF1, PSPC1, SNRNP70, CPSF6, SF3B2, RPS13, FUBP3, HNRNPUL1, CSTF1, DDX5, SNRPD2, HNRNPDL, FUS, ELAVL1, NUDT21, HNRNPR, SEC13, CARM1, ACTL6A, HNRNPA0, DDX3X, KPNB1, TUBG1, SNRPA
nucleus	SNRPD3, RPA1, HNRNPAB, NUP93, SMARCC2, EWSR1, SNRPN, DDX17, SF1, PSPC1, SNRNP70, CPSF6, CNOT1, SF3B2, DDX6, RPS13, FUBP3, HNRNPUL1, CSTF1, DDX5, SNRPD2, HNRNPDL, FUS, ELAVL1, NUDT21, HNRNPR, SEC13, CARM1, ACTL6A, HNRNPA0, DDX3X, KPNB1, TUBG1, SNRPA
protein-containing complex	SNRPD3, RPA1, HNRNPAB, NUP93, SMARCC2, SNRPN, RPL18A, DDX17, SF1, SNRNP70, CPSF6, SEC23A, CNOT1, SF3B2, DDX6, RPS13, FUBP3, HNRNPUL1, CSTF1, DDX5,

	SNRPD2, HNRNPDL, ELAVL1, NUDT21, HNRNPR, SEC13, ACTL6A, HNRNPA0, DDX3X, KPNB1, TUBG1, SNRPA, RPL31
U1 snRNP	SNRPD3, SNRPN, SNRNP70, SNRPD2, SNRPA
spliceosomal snRNP complex	SNRPD3, SNRPN, SNRNP70, SF3B2, SNRPD2, SNRPA

GO molecular function	Gene names
RNA binding	SNRPD3, HNRNPAB, EWSR1, SNRPN, RPL18A, DDX17, SF1, PSPC1, SNRNP70, CPSF6, CNOT1, SF3B2, DDX6, RPS13, FUBP3, HNRNPUL1, CSTF1, DDX5, SF1, SNRPD2, HNRNPDL, FUS, ELAVL1, NUDT21, HNRNPR, HNRNPA0, DDX3X, KPNB1, SNRPA, RPL31
nucleic acid binding	SNRPD3, RPA1, HNRNPAB, SMARCC2, EWSR1, SNRPN, RPL18A, DDX17, SF1, PSPC1, SNRNP70, CPSF6, CNOT1, RPA1, SF3B2, DDX6, RPS13, FUBP3, HNRNPUL1, CSTF1, DDX5, SF1, SNRPD2, HNRNPDL, FUS, ELAVL1, NUDT21, HNRNPR, CARM1, ACTL6A, HNRNPA0, DDX3X, KPNB1, SNRPA, RPL31
mRNA binding	HNRNPAB, SF1, SNRNP70, CPSF6, DDX6, RPS13, FUBP3, DDX5, FUS, ELAVL1, NUDT21, HNRNPR, HNRNPA0, DDX3X
hetetrocyclic compound binding	SNRPD3, RPA1, HNRNPAB, SMARCC2, EWSR1, SNRPN, RPL18A, DDX17, SF1, PSPC1, SNRNP70, CPSF6, CNOT1, RPA1, SF3B2, DDX6, RPS13, FUBP3, HNRNPUL1, CSTF1, DDX5, SF1, SNRPD2, HNRNPDL, FUS, ELAVL1, NUDT21, HNRNPR, CARM1, ACTL6A, HNRNPA0, DDX3X, KPNB1, TUBG1, SNRPA, RPL31
organic cyclic compound binding	SNRPD3, RPA1, HNRNPAB, SMARCC2, EWSR1, SNRPN, RPL18A, DDX17, SF1, PSPC1, SNRNP70, CPSF6, CNOT1, RPA1, SF3B2, DDX6, RPS13, FUBP3, HNRNPUL1, CSTF1, DDX5, SF1, SNRPD2, HNRNPDL, FUS, ELAVL1, NUDT21, HNRNPR, CARM1, ACTL6A, HNRNPA0, DDX3X, KPNB1, TUBG1, SNRPA, RPL31
protein binding	SNRPD3, RPA1, HNRNPAB, NUP93, SMARCC2, EWSR1, SNRPN, TFG, RPL18A, DDX17, SF1, PSPC1, SNRNP70, CPSF6, SEC23A, CNOT1, SF3B2, DDX6, RPS13, FUBP3, HNRNPUL1, CSTF1, DDX5, SNRPD2, HNRNPDL, FUS, ELAVL1, NUDT21, HNRNPR, SEC13, CARM1, ACTL6A, HNRNPA0, DDX3X, KPNB1, TUBG1, SNRPA, RPL31
mRNA 3'-UTR binding	DDX5, FUS, ELAVL1, NUDT21, HNRNPA0
RNA helicase activity	DDX17, DDX6, DDX5, DDX3X
ATP-dependent activity, acting on RNA	DDX17, DDX6, DDX5, DDX3X
pre-mRNA branch point binding	SF1
mRNA 3'UTR AU-rich region binding	ELAVL1, NUDT21, HNRNPA0

Supplementary table 11A: Results from PANTHER enrichment analysis for 18 proteins identified to bind Kpnβ1 in cancer cells only

GO biological process	# in Homo Sapiens reference list	#	expected	Fold enrichment	p value	False discovery rate (FDR)
cytoplasmic translation	124	7	0.11	64.57	1.07E-11	1.68E-07
gene expression	2314	13	2.02	6.43	2.33E-09	1.83E-05
translation	379	7	0.33	21.13	2.04E-08	1.07E-04
peptide biosynthetic process	408	7	0.36	19.62	3.36E-08	1.32E-04
amide biosynthetic process	527	7	0.46	15.19	1.87E-07	5.88E-04
cellular nitrogen compound metabolic process	3573	13	3.12	4.16	4.70E-07	1.05E-03
peptide metabolic process	537	7	0.47	14.91	2.13E-07	5.55E-04
ribonucleoprotein complex biogenesis	449	6	0.39	15.29	1.66E-06	3.26E-03
cellular amide metabolic process	802	7	0.7	9.98	3.05E-06	4.78E-03
RNA processing	868	7	0.76	9.22	5.13E-06	7.31E-03
GO cellular component						
cytosolic ribosome	105	7	0.09	76.26	3.51E-12	7.17E-09
ribosomal subunit	189	7	0.17	42.36	1.84E-10	1.88E-07
ribosome	228	7	0.2	35.12	6.56E-10	4.47E-07
ribonucleoprotein complex	714	9	0.62	14.42	2.81E-09	1.15E-06
nuclear lumen	4494	16	3.93	4.07	2.62E-09	1.34E-06
organelle lumen	5614	16	4.91	3.26	8.03E-08	2.73E-05
cytosolic large ribosomal subunit	59	4	0.05	77.55	2.35E-07	5.33E-05
nucleus	7682	17	6.72	2.53	6.21E-07	1.27E-04
nucleoplasm	4130	13	3.61	3.6	2.65E-06	4.92E-04
large ribosomal subunit	115	4	0.1	39.79	3.04E-06	5.17E-04
GO molecular function						
RNA binding	1666	16	1.46	10.99	4.75E-16	2.36E-12
structural constituent of ribosome	168	7	0.15	47.66	8.31E-11	2.07E-07
nucleic acid binding	4007	16	3.5	4.57	4.44E-10	7.36E-07
structural molecule activity	796	9	0.7	12.93	7.18E-09	8.93E-06
heterocyclic compound binding	5983	16	5.23	3.06	2.12E-07	2.11E-04
organic cyclic compound binding	6054	16	5.29	3.02	2.54E-07	2.10E-04
mRNA binding	326	5	0.29	17.54	7.48E-06	5.32E-03
nuclear export signal receptor activity	11	2	0.01	> 100	5.59E-05	3.48E-02

Supplementary table 11B: Gene names in each GO category identified in PANTHER enrichment analysis for 18 proteins identified to bind Kpnβ1 in cancer cells only

GO biological process	Gene names
cytoplasmic translation	RPL10, RPS6, RPL13A, RPL7, RPS11, RPL18, RPS4X,
gene expression	CPSF7, SRSF1, RPL10, RPS6, RPL13A, RPL7, RPS11, HNRNPH3, RPL18, FIP1L1, NUP214, RPS4X, SRSF3,
translation	RPL10, RPS6, RPL13A, RPL7, RPS11, RPL18, RPS4X,
peptide biosynthetic process	RPL10, RPS6, RPL13A, RPL7, RPS11, RPL18, RPS4X,
amide biosynthetic process	RPL10, RPS6, RPL13A, RPL7, RPS11, RPL18, RPS4X,
cellular nitrogen compound metabolic process	CPSF7, SRSF1, RPL10, RPS6, RPL13A, RPL7, RPS11, HNRNPH3, RPL18, FIP1L1, RAN, RPS4X, SRSF3,
peptide metabolic process	RPL10, RPS6, RPL13A, RPL7, RPS11, RPL18, RPS4X,
ribonucleoprotein complex biogenesis	CPSF7, SRSF1, RPL10, RPS6, RPL7, RAN
cellular amide metabolic process	RPL10, RPS6, RPL13A, RPL7, RPS11, RPL18, RPS4X,
RNA processing	CPSF7, SRSF1, RPS6, RPL7, HNRNPH3, FIP1L1, SRSF3
**GO cellular component**	**Gene names**
cytosolic ribosome	RPL10, RPS6, RPL13A, RPL7, RPS11, RPL18, RPS4X
ribosomal subunit	RPL10, RPS6, RPL13A, RPL7, RPS11, RPL18, RPS4X
ribosome	RPL10, RPS6, RPL13A, RPL7, RPS11, RPL18, RPS4X
ribonucleoprotein complex	SRSF1, RPL10, RPS6, RPL13A, RPL7, RPS11, HNRNPH3, RPL18, RPS4X
nuclear lumen	CPSF7, SRSF1, RPS6, RPL13A, RPL7, FUBP1, RPS11, HNRNPH3, RPL18, FIP1L1, NUP214, CCAR1, RAN, RPS4X, HNRNPUL2, SRSF3
organelle lumen	CPSF7, SRSF1, RPS6, RPL13A, RPL7, FUBP1, RPS11, HNRNPH3, RPL18, FIP1L1, NUP214, CCAR1, RAN, RPS4X, HNRNPUL2, SRSF3
cytosolic large ribosomal subunit	RPL10, RPL13A, RPL7, RPL18
nucleus	CPSF7, SRSF1, RPL10, RPS6, RPL13A, RPL7, FUBP1, RPS11, HNRNPH3, RPL18, FIP1L1, NUP214, CCAR1, RAN, RPS4X, HNRNPUL2, SRSF3
nucleoplasm	CPSF7, SRSF1, RPS6, FUBP1, RPS11, HNRNPH3, FIP1L1, NUP214, CCAR1, RAN, RPS4X, HNRNPUL2, SRSF3
large ribosomal subunit	RPL10, RPL13A, RPL7, RPL18
**GO molecular function**	**Gene names**
RNA binding	CPSF7, SRSF1, RPL10, RPS6, RPL13A, RPL7, FUBP1, RPS11, HNRNPH3, RPL18, FIP1L1, CCAR1, RAN, RPS4X, HNRNPUL2, SRSF3
structural constituent of ribosome	RPL10, RPS6, RPL13A, RPL7, RPS11, RPL18, RPS4X
nucleic acid binding	CPSF7, SRSF1, RPL10, RPS6, RPL13A, RPL7, FUBP1, RPS11, HNRNPH3, RPL18, FIP1L1, CCAR1, RAN, RPS4X, HNRNPUL2, SRSF3
structural molecule activity	RPL10, RPS6, RPL13A, MYL6, RPL7, RPS11, RPL18, NUP214, RPS4X
heterocyclic compound binding	CPSF7, SRSF1, RPL10, RPS6, RPL13A, RPL7, FUBP1, RPS11, HNRNPH3, RPL18, FIP1L1, CCAR1, RAN, RPS4X, HNRNPUL2, SRSF3
organic cyclic compound binding	CPSF7, SRSF1, RPL10, RPS6, RPL13A, RPL7, FUBP1, RPS11, HNRNPH3, RPL18, FIP1L1, CCAR1, RAN, RPS4X, HNRNPUL2, SRSF3
mRNA binding	CPSF7, SRSF1, RPL13A, RPL7, FUBP1
nuclear export signal receptor activity	NUP214, RAN
